# Supplementary material for: Carbohydrate-active enzymes from Akkermansia muciniphila break down mucin O-glycans to completion
Source: Nat Microbiol. 2025 Jan 31;10(2):585–98. doi: 10.1038/s41564-024-01911-7 (PMC11790493; doi:10.1038/s41564-024-01911-7)

nature microbiology

Supplementary information

https://doi.org/10.1038/s41564-024-01911-7

Carbohydrate-active enzymes from *Akkermansia muciniphila* break down mucin *O*-glycans to completion

In the format provided by the authors and unedited

Supplementary Information

Carbohydrate-active enzymes from *Akkermansia muciniphila* breakdown mucin O-glycans to completion

Cassie R. Bakshani[1], Taiwo O. Ojuri[1], Bo Pilgaard[2], Jesper Holck[2], Ross McInnes[1], Radoslaw P. Kozak[3], Maria Zakhour[4], Sara Çakaj[1], Manon Kerouedan[1], Emily Newton[1], David N. Bolam[4], and Lucy I. Crouch[1*]

1 Institute of Microbiology and Infection, Department of Microbes, Infection and Microbiomes, College of Health and Medicine, University of Birmingham, Birmingham, B15 2TT.
2 Protein Chemistry and Enzyme Technology Section, DTU Bioengineering, Department of Biotechnology and Biomedicine, Technical University of Denmark, 2800 Kgs, Lyngby, Denmark.
3 Ludger Ltd, Culham Campus, Abingdon, OX14 3EB, UK.
4 Biosciences Institute, Medical School, Newcastle University, Newcastle upon Tyne, NE2 4HH, UK.
*Address correspondence to l.i.crouch@bham.ac.uk

Contents

Supplementary Discussion
RNA-seq
Pangenome analysis
Further description of enzyme specificities and analysis of structural models
- α-galactosidases
  - GH27
  - GH97
  - GH36
- α-GalNAc'ases
  - GH31
- α-GlcNAc'ases
- α-sialidases
- α-fucosidases
- β-galactosidases
- β-HexNAc'ases

Activity of AM CAZymes against human milk oligosaccharides
Interaction with GAGs
Growth of AM on high-mannose N-glycoproteins
Starch-degrading activities
The extraordinary import system of AM
CAZymes where no activities could be identified.
Supplementary Results

Supplementary Discussion

RNA-seq

The transcriptional response of AM varied greatly when grown with the addition of mucin compared to glucose (Figure 1). A total of 201 genes (9.3%) were differentially expressed with 76 significantly upregulated (Log2 Fold Change > 1.5 & FDR < 0.05) and 125 significantly downregulated (Log2 Fold Change < -1.5 & FDR < 0.05). Of the 67 enzymes in this study, 22 were significantly upregulated and 0 were significantly downregulated.

Pangenome analysis

One way to assess the importance of the AM CAZymes to the species in terms of mucin degradation is to review how prevalent and conserved the enzymes are over multiple *A. muciniphila* isolates (Supplementary Figure 2). In this analysis we also included *Akkermansia biwaensis* WON2089, twelve *Akkermansia massiliensis* strains, and five *Candidatus Akkermansia timonensis* strains. The latter two species have recently been reclassified as their own species but were previously cluster II and III of *A. muciniphila*[1]. The isolates included in this analysis were from a range of human faecal sources, including different collection efforts, geographical location, adults, and children.

The striking aspect of this analysis was how highly most of these enzymes were conserved between *A. muciniphila* strains (>90 %). There were a few examples of enzymes not being present in many strains (e.g. Amuc_0875^{GH16} and Amuc_0146^{GH29}), but generally enzymes were present. As another generalisation, the other species assessed did have close homologues of the CAZymes in *A. muciniphila* BAA-835, with *A. massiliensis* having higher identity than *A. biwaensis* and *Candidatus A. timonensis*.
Additional enzymes were also identified during this analysis:

- A GH2 in some *A. muciniphila* strains.
- A GH2 in *A. biwaensis* and *Candidatus A. timonensis*.
- A GH16 in *A. biwaensis* and *Candidatus A. timonensis*.
- A GH27 in *A. massiliensis.*
- A GH27 in *A. biwaensis* and *Candidatus A. timonensis*.
- A GH29 in many of the *A. muciniphila* strains
- A GH29 in most *A. massiliensis.*
- A GH29 in *A. biwaensis* and *Candidatus A. timonensis*.
- A GH36 in *A. biwaensis* and *Candidatus A. timonensis*.
- A GH36 in *A. massiliensis, A. biwaensis* and *Candidatus A. timonensis*.
- A GH84 in most of all strains and species assessed.
- A GH95 in *A. massiliensis.*
- A GH95 in *A. biwaensis* and *Candidatus A. timonensis*.

Further description of enzyme specificities and analysis of structural models

<u>α-galactosidases</u>

GH27: The characterised activities from GH27 family members so far include α-galactosidases, α-GalNAc'ases, β-L-arabinopyranosidases, and isomaltodextrinases. Bacterial GH27 family members with activity against α-Gal have been characterised in the context of plant polysaccharides, pectic galactan and galactomannan, for example, and there are no structures of these enzymes[2, 3]. Therefore, Amuc_1187^{GH27} is the first example of an α-galactosidase GH27 family member of prokaryotic origin with specificity for animal-type substrates rather than those of plant. The structural and mutational studies of two GH27 enzymes from humans has previously been insightful in terms of understanding their differing specificities for α-galactose or α-GalNAc[4]. In humans, two GH27 enzymes, one α-galactosidase and one α-GalNAc'ase (48 % sequence identity), are involved in the recycling of glycolipids and glycoproteins. Deficiencies in these enzymes in humans is linked to several lysosomal storage diseases due to the accumulation of glycoconjugates. In contrast to

Amuc_1187^{GH27}, the human enzymes can accommodate the fucose in the blood group structures to hydrolyse either α-Gal or α-GalNAc[5]. Between these two human enzymes, eleven out of thirteen amino acids coordinating the sugar in the -1 subsite were identical. Those that differed were key to the accommodation of either an α-Gal or α-GalNAc. In the α-GalNAc'ase, there were relatively small residues (serine and alanine) coordinating at the N-acetyl group of the GalNAc, but in the α-galactosidase these residues were larger (glutamate and leucine) thus prohibiting the binding of GalNAc (Supplementary Figure 11). Mutation of the key residues in both enzymes to that of the other, allowed the swapping of specificities[4]. Amuc_1187^{GH27} has approximately 30 % sequence identity to the human enzymes, but in terms of the active site, the residues coordinating the -1 sugar in the two human enzymes are generally conserved in Amuc_1187^{GH27} and it has a serine and asparagine at the critical locations (S320 and N321), aligning with the observed activity of α-galactosidase (Supplementary Figure 11).

GH97: Amuc_1420^{GH97} was able to hydrolyse galactose from PGMIII from samples with or without sialidase and fucosidase pre-treatment, comparable to that of the other AM α-galactosidases (Figure 1 and Supplementary Figure 14). It was also able to hydrolyse α-galactose capping substrates (not blood groups) and had a preference for the trisaccharide globotriose. Intrigued by this contrast in activities, we analysed an Alphafold model of Amuc_1420^{GH97} alongside available GH97 structures. The only GH97 α-galactosidase crystal structure with a ligand is that of BT1871 from *Bacteroides thetaiotaomicron*, which has been crystallised with a lactose that also had an α-linked galactose to the Glc through a 1,1-linkage[6]. This ligand was overlaid into the Alphafold model for Amuc_1420^{GH97} and suggests there would be significant interactions with the sugar in the +2 subsite through a tryptophan (W461) (Supplementary Figure 13). This likely explains why poorer activity is detected against disaccharide host-type substrates compared to trisaccharides and complex substrates.

GH36: There are three putative GH36 enzymes encoded in the AM genome, and these have relatively low sequence identity between them (Supplementary Table 2). The family members characterised so far display exo-acting activities towards α-Gal or α-GalNAc, with attention predominantly on the former in terms of raffinose-type oligosaccharides and galactomannan. Amuc_0855^{GH36} was able to liberate galactose from Galili antigen, globotriose, and PGMIII with or without sialidase and fucosidase treatment (Extended Data 6). Galili antigen is expressed by most mammals and some other animals, but not humans, apes, and Old World monkeys[7]. Therefore, gut microbes would only have access to this structure through animal products in the diet of humans. On the phylogenetic tree, this enzyme clusters with α-galactosidases characterised to be active against both galactomannan and raffinose-type oligosaccharides (Supplementary Figure 12). Amuc_0855^{GH36} also had trace activity against these substrates, but no growth was observed on these substrates for AM, so this is unlikely to be physiologically relevant (Extended Data 1).

The whole cell assays showed no activity against the defined substrates with α-galactose at the non-reducing end, suggesting that none of the AM CAZymes acting on these epitopes are present on the outside of the cell (Supplementary Figures 3 and 4).

<u>α-GalNAc'ases</u>
GH31: A model of Amuc_1008^{GH31} was compared to the structure of the *E. faecalis* enzyme, which is the only structure from this subfamily. An overlay indicates that the structure of Amuc_1008^{GH31} has extra C-terminal domains compared to *Ef*GH31 (Supplementary Figure 10). One of these extra domains is classified as a family 32 carbohydrate binding domain (CBM32), which have been to show a variety of different sugars and glycans. The GH31 from *Clostridium perfringens* with the same activity as Amuc_1008^{GH31} was also has a CBM32, which has been characterised to bind GalNAc[8]. Tn antigen that was crystallised in the active site of *Ef*GH31 overlays well into the active site of Amuc_1008^{GH31} (Supplementary Figure 10).

The residues interacting with the Tn-antigen in the *Ef*GH31 crystal structure are all conserved in Amuc_1008^{GH31}.

α-GlcNAc'ases

There are currently five characterised GH89 family members all with specificity for this α-linked GlcNAc[9]. The first enzyme identified to have specificity for stomach epitope was one from *C. perfringens*[10] and was subsequently crystallised with this disaccharide in the active site (4A4A; Supplementary Figure 15)[11]. Other members of the GH89 family are active against heparan sulfate and there is a crystal structure of this from *H. sapien* (4XWH)[12]. Mutations of this GH89 gene leads to Sanfilippo syndrome or mucopolysaccharoidosis III, where recycling of heparan sulfate is impaired, and there is currently no cure for this or treatments to slow disease progression. Detailed characterisations of glycobiology (or absence of) leading to disease may highlight potential ideas for treatments in the future.

Models of the two GH89 enzymes from AM and these two crystal structures were superimposed and compared (Supplementary Figure 15). The active site of Amuc_1220^{GH89} has a similar conformation to the pocket from *C. perfringens*, whereas the substrate pocket of Amuc_0060^{GH89} has a similar conformation to the pocket from *H. sapien*. This implies that the substrates for Amuc_1220^{GH89} and Amuc_0060^{GH89} are GlcNAcα1,4-Gal stomach epitope and heparan sulfate or other GlcNAc-containing glycosaminoglycans, respectively. The data obtained in this report show that Amuc_1220^{GH89} is removing GlcNAc from PGMII, but we did not observe any activity for Amuc_0060^{GH89} against GAGs incubated alone or in combination with other AM CAZymes (See GAGs section below).

Amuc_1220^{GH89} is predicted to be localised to the periplasm (Supplementary Table 1) and whole cell assays using GlcNAcα1,4-Gal suggest that this epitope is not broken down on the outside of the cell (Supplementary Figure 3 and 4).

α-sialidases

The two GH33 family members (Amuc_0625^{GH33} and Amuc_1835^{GH33}) are broad-acting sialidases in terms of linkage, whereas the Amuc_1547^{GH181} is α2,3-specific[13]. In this work, we explored whether these enzymes act upon ganglioside structures, which have structural similarities to O-glycans. Gangliosides are important components of eukaryotic plasma membranes and are linked to several genetic diseases and pathogen interactions[14]. Gangliosides (GM3 and GD3; Figure 1) are also found in the membranes of milk fat globules in breast milk, so it is likely that gut microbes in the infant gut would encounter these[15]. Therefore, gangliosides would be found in the large intestine at all life stages from both host and dietary sources. We found that the two GH33 enzymes from AM can act on the relatively complex GD1a and GT1b ganglioside structures, with one or two sialic acids being removed. The inability to remove one of the sialic acids is likely due to this being the branching sialic acid and therefore relatively difficult to access. However, if we removed the capping β1,3-linked galactose with β-galactosidases from AM, then it was possible for Amuc_1835^{GH33} to remove all sialic acids (Extended Data 7). Importantly, the removal of this galactose produces Sda antigen, which is present in 91 % of humans and is expressed in mucosal surfaces and other tissues. Therefore, is it valuable to understand that sialidases from AM can act on Sda antigen and gangliosides. Amuc_1835^{GH33} was recently highlighted as important for growth on mucin using a Tn-seq mutant library[16], and Amuc_0625^{GH33} and Amuc_1547^{GH181} were highly conserved (>90 % sequence identity) across all *Akkermansia* species assessed (Supplementary Figure 2).

α-fucosidases

Between them, the GH29 and GH95 enzymes from AM can degrade a wide variety of fucose-rich substrates (Extended Data 8 and Supplementary Figure 16)[13, 17]. These enzymes cannot access the fucose in blood group A and B (BGA and B) structures, so these glycan epitopes are first partially broken down by other CAZymes encoded in the AM genome to produce blood

group H structures. Amuc_1120^{GH95} is a broad-acting α1,2-fucosidase, whereas Amuc_0010^{GH29} had specificity to α1,2-linked fucose on lactose/LacNAc (β1,4), but not Lacto-N-biose (β1,3). This means that Amuc_0010^{GH29} exhibits specificity towards type II blood group structures (not type I) and the lacto-N-*neo*tetraose HMO series (not the lacto-N-tetraose). In contrast, Amuc_0846^{GH29} and Amuc_0392^{GH29} have comparable activities with activities against α1,3 and 4-linked fucose, but Amuc_0392^{GH29} did exhibit some slightly broader activity by also being able to liberate galactose from some sulfated Lewis structures and sialylfucosyllacto-N-tetraose. For Amuc_0186^{GH95}, we were only able to find activity against 2'-fucosyllactose and for Amuc_0146^{GH29} we could find no activities, including with pNP-monosaccharides.

Amuc_0146^{GH29} and Amuc_1120^{GH95} were recently highlighted as important for growth on mucin using a TnSeq mutant library[16] and Amuc_0392^{GH29} is highly conserved across different *Akkermansia* species (Supplementary Figure 2). Furthermore, five out of nine of the sialidases and fucosidases were highlighted by proteomics when AM was grown on human milk (Supplementary Table 1)[18].

β-galactosidases

The five GH2 enzymes show low sequence identity, with the highest being 39 % between Amuc_0824 and Amuc_1666 (Supplementary Table 3). The two GH35 enzymes share 37 % identity and the two GH43_24 enzymes have 87 % identity. Amuc_0539^{GH2} was found to be specific to β1,4-linked Gal with either a Gal or GalNAc in the +1 position, with some activity against lactose (Figure 3 and Supplementary Figures 18-21). Conversely, Amuc_0771^{GH35} and Amuc_1666^{GH2} could not hydrolyse these substrates and instead required a Glc/GlcNAc in the +1 position for β1,4-linked Gal substrates. Amuc_1666^{GH2} was also able to act on TriLacNAc and Lacto-N-neotetraose, but only Galβ1,3Glc out of those substrates with β1,3-linked Gal. Amuc_0771^{GH35} was much broader with the capacity to also hydrolyse β1,3-linked Gal with Glc, GalNAc, and Gal in the +1 position. This is also the only enzyme that could remove both Gal sugars from the branching lacto-N-neohexaose. These branching structures, formed through a GlcNAcβ1,6Gal linkage, are important due to their prevalence in humans (99 % of population) and are referred to as "I antigens" (no branching is referred to as "i antigen"). Mucins across most tissues will have this branching[14]. Complementary to the screening carried out in this report, Amuc_1666^{GH2} has previously been shown to have a preferences for LacNAc[19] and Amuc_0771^{GH35} has also previously been shown to have activity against mucin core 1 and core 2 structures, which both have β1,3 linkages[20].

Amuc_0824^{GH2} is highly specific towards β1,3-linked Gal with no preference observed for what occupies the +1 positions and no activity against β1,4-linked Gal substrates. In line with our findings, Amuc_0824^{GH2} has previously been shown to have preferences for Galacto-N-biose[19]. Amuc_1686^{GH35} showed very similar specificity to Amuc_0824^{GH2} except that it could also accommodate β1,6-linked Gal with Gal/GalNAc in the +1 (no β1,6-linked substrates with a Glc-type sugar were available to test) and has previously been shown to have a preference for Galβ1,3GalNAc[21]. Amuc_0290^{GH2} can accommodate β1,4 and β1,6-linked Gal substrates with less preference for β1,3-linked Gal substrates. Finally, Amuc_1667^{GH2} was inactive against most substrates, with only some activity towards the ganglioside core structure GA1. Concerning biantennary complex N-glycans, all three enzymes that can accommodate other Galβ1,4GlcNAc structures, could remove Gal from N-glycan that had been released from the protein and de-sialylated. This is unlikely to be physiologically relevant, however, as AM will not grow on this glycoprotein substrate despite similarities to O-glycans (Extended Data 1).

Regarding larger more complex substrates, Amuc_0771^{GH35} was able to release the largest concentration of galactose from BSM (Extended Data 5). Amuc_0824^{GH2} was also able to release galactose, which complements the understanding that this substrate constitutes core 1 structures, but Amuc_0771^{GH35} can release a higher concentration of galactose indicating that it can accommodate the mucin polypeptide more easily than Amuc_0824^{GH2}.

Of these enzymes, Amuc_0824^{GH2} is predicted to localise to the cell surface, while the rest are predicted to reside in the periplasm (Supplementary Table 1). Notably, the mucin-grown whole cells showed rapid degradation of Lacto-N-biose, one of the substrates for this enzyme, and this was reproducible over two different experiments (Supplementary Figure 3 and 4). This was the fastest degradation of a substrate, with complete hydrolysis recorded in 30 minutes. Lactose and Galβ1,4GalNAc are not substrates for this enzymes and the whole cell assays did not show degradation of these substrates (Figure 3). Amuc_0824^{GH2} cannot be directly linked to this activity, but techniques that have been developed for the genetic manipulation of AM may lend themselves to these types of experiments in the future.

Of the fourteen enzymes identified as having >90 % sequence identity conservation across the different *Akkermansia* species we assessed, all the GH2 and GH35 family members, apart from Amuc_0771^{GH35} and Amuc_1667^{GH2}, were in this group (Supplementary Figure 2) and Amuc_0290^{GH2}, Amuc_0539^{GH2}, Amuc_1666^{GH2} and Amuc_1667^{GH2} were highlighted as important for growth on mucin[16]. This emphasises the important role these β-galactosidases play in nutrient acquisition for AM (Supplementary Tabel 1). No activities for the GH43_24 enzymes were observed against the substrates tested here, but Amuc_0698^{GH43} was active against pNP-Gal. Interestingly, Amuc_0697^{GH43} was also highlighted as important for growth on mucin[16], conversely however, Amuc_0697^{GH43} was not well conserved between *A. muciniphila* strains (Supplementary Figure 2).

One of the striking aspects of CAZymes from AM is the length of the putative enzymes, which structural modelling predicts to be a high number of accessory modules accompanying the catalytic module. This is exemplified by the β-galactosidases (Supplementary Figure 23). How these accessory modules contribute to function is still yet to be determined. The active sites of the different GH2 and GH35 enzyme models were compared to relevant existing structures to provide insight into the different specificities being observed. Amuc_0539^{GH2} has specificity to β1,4-linked Gal with either a Gal or GalNAc in the +1 position, not Glc/GlcNAc. Galactose and glucose type sugars have the hydroxyl in the axial and equatorial positions at C4, respectively. The difference means that β1,4-linked sugars have very different overall conformations, and this is reflected in some of the specificities observed for the β-galactosidases. Models of different disaccharides are included to contextualise these possible conformational differences (Supplementary Figure 24). Current structures do not include any examples with substrates with Gal/GalNAc in the +1 subsite, but LacNAc and lactose examples are available. When we overlay LacNAc from *Streptococcus pneumoniae* GH2 (4CUC) into the active site of Amuc_0539^{GH2} a tryptophan is highlighted as being very close to the +1 GlcNAc. This suggests that this amino acid may contribute significantly to excluding substrates with Glc-type sugars in the +1 subsite. Similarly, Amuc_0290^{GH2}, which has a much broader activity, has W686 in the same region, but rotated approximately 180 ° along its plane. This may act to contribute to binding glycans rather than having a selectivity role. Apart from these observations, the active sites of these two enzymes are predicted to be generally quite open.

In contrast to the GH2 and GH35 enzymes, the GH43 proteins only constitute a catalytic module and they are very similar in sequence. The only difference is a short section of sequence that corresponds to an extended loop in Amuc_0697^{GH43} that protrudes towards the active site (Supplementary Figure 21).

β-HexNAc'ases

The identity between the GH20 sequences from AM is generally low ~20-30 % with the exceptions of 72 % between Amuc_2018^{GH20} and Amuc_2019^{GH20}. These enzyme sequences were put into phylogenetic trees alongside other characterised members of their families (Supplementary Figures 25). These showed that the enzyme sequences from the AM genome clustered with previously characterised enzymes with β-HexNAc'ase activities. The first step

in this analysis was to screen a panel of defined oligosaccharides (Supplementary Figures 27-29). The enzyme with the broadest activity was Amuc_0868^{GH20} that could degrade most of the substrates provided, apart from GlcNAcβ1,3Man and β1,2-linked GlcNAc on biantennary N-glycans, to which it was only partially active. This enzyme was previously shown to prefer GlcNAc over GalNAc[22]. Amuc_2019^{GH20} showed a similar pattern but exhibited only partial activity against the P antigen. Again, Amuc_2136^{GH20} showed a similar pattern to Amuc_0868^{GH20}, but with no activity against the β1,4-linked GalNAc on GA2. Amuc_2136^{GH20} has also previously been shown to prefer GlcNAc over GalNAc[23]. Amuc_0369^{GH20} was also relatively broad-acting, but with a different pattern; it was able to access most of the host-type glycans tested and demonstrated relatively less activity against chitooligosaccharides. Amuc_2018^{GH20} showed a similar pattern to these three broad-acting GH20 enzymes, but with negligible activity against chitooligosaccharides and no activity against β1,4-linked GalNAc and P antigen. Amuc_2018^{GH20} has previously been shown to prefer GlcNAc over GalNAc and have some activity against N-glycan structures[22]. Amuc_0052^{GH84} showed specificity towards GlcNAc over GalNAc substrates and prefers smaller substrates (disaccharides). From the substrate screen, Amuc_1669^{GH20} is selective for host glycans with a preference for GlcNAcβ1,3Gal substrates. It can also hydrolyse GalNAc in the context of globoside P antigen, but not ganglioside GA2. Amuc_2148^{GH20} shows very similar pattern of activity to Amuc_1669^{GH20} but is even more specific as it will not act on P antigen. Amuc_1924^{GH20} has a preference for GalNAc in the -1 subsite but was unable to accommodate P antigen and this also may be due to overall glycan conformation. Amuc_1032^{GH20}, acted only upon GlcNAcβ1,3Gal in the context of TriLacNAc and lacto-N-triose, but not GlcNAcβ1,3Gal alone, indicating that this enzyme prefers substrates that are at least three sugars long (i.e. a sugar occupying the +2 subsite). Finally, Amuc_0803^{GH123} had strict specificity towards the GalNAc substrates, including GA2. Exact specificities could not be determined for Amuc_0397^{GH20} and Amuc_1815^{GH20}. Both only had trace activity against GalNAcβ1,3Gal, but it may be that they are active on longer versions of this substrate. Specificities could also not be found for Amuc_2109^{GH3}, with only trace activity against chitooligosaccharides and GaNcAcβ1,3Gal.

In terms of the redundancy seen for the β-galactosidases and β-HexNAc'ases, the expression and localisation of these different enzymes in AM may require this redundancy in specificity for such a complicated substrate to maximise AMs access to the sugars in mucin. The conservation of most of the β-galactosidases and β-HexNAc'ases over species and strains supports this hypothesis.

Activity of AM CAZymes against human milk oligosaccharides

The growth of AM on human milk has previously been demonstrated and proteomics highlighted a high proportion of the AM CAZymes [18](Supplementary Table 1). The structures of human milk oligosaccharides have many similarities to mucin O-glycans, so we performed sequential reactions on two commercially available products. We first tested the AM CAZymes that remove the different α-linked capping monosaccharides (Supplementary Figures 30). Interestingly, we found no release of sialic acid, despite one of the substrates being labelled as such. In terms of fucosidases, most of the AM enzymes could act on one of the products. Discrepancies were observed between the activities of Amuc_0010^{GH29} and Amuc_1120^{GH95} against HMOs, with the latter releasing more fucose and, therefore, these HMOs have predominantly lacto-N-tetraose structures. This observation corresponds to the understanding of type II structures being present on red blood cells and type I structures are associated with glycan decorations on secretions[14]. Notably, Amuc_0010^{GH29} clusters on a phylogenetic tree with 'Mfuc5' from a soil metagenome and has comparable activities[24]. For the AM CAZymes with specificity for removing α-linked GlcNAc, GalNAc, or galactose, no obvious activity was observed.

The panel of β-galactosidases were then tested against the HMOs which had also been pre-treated with two fucosidases (Supplementary Figure 31). The addition of Amuc_0290^{GH2}, which had the broadest specificity in the substrate screen, had the most activity (shifting all

the bands up the most). Much less galactose could be released without the addition of fucosidases (Supplementary Figure 20). Finally, the panel of β-HexNAc'ases was then tested against the HMOs that had been sequentially treated with fucosidases and galactosidases. The specificities observed for the defined substrate screen facilitates the probing of more complicated structures in more detail and exemplifies how these enzymes may be useful in other contexts. For example, Amuc_0803^{GH123} showed no obvious activity towards the HMO substrates, implying that no β-linked GalNAc is present. Furthermore, Amuc_1032^{GH20} is providing the same pattern as the broad-acting β-HexNAc'ases, so the glycans in this assay were at least 3 sugars long. Finally, Amuc_0052^{GH84} did not breakdown the glycans as much as the broad-acting β-HexNAc'ases, emphasizing its preference for smaller substrates.

Interaction with GAGs
The PL38 enzymes characterised to date have specificities for glucuronan and alginate[25, 26], but screens of these substrates did not reveal any activity (Extended Data 10). Characterised GH105 enzymes have been found to be active against the unsaturated products of PLs [27]. We found no activities for GH105 against any of the other substrates used in this work. Therefore, we tested host GAGs that are known substrates for some PL families and are commonly available to the microbiota in the large intestine. Amuc_0778^{PL38} was found to be active against hyaluronic acid (HA), chondroitin sulfate A (CSA), chondroitin sulfate C (CSC) and dermatan sulfate (DS) (Extended Data 10 and Supplementary Figures 36-38). Amuc_0863^{GH105} had little or no activity when it was incubated alone with these substrates but was able to act on the products of the PL38 from all four substrates.

The Michaelis-Menten kinetic analysis of Amuc_0778^{PL38} against the different GAGs revealed the highest catalytic efficiency (k_{cat}/K_M) towards HA, followed closely by CSA and four- and eight-fold lower towards CSC and DS, respectively (Supplementary Table 6). An attempt to resolve the kinetic parameters of Amuc_0863^{GH105} was performed by preparing substrate using the Amuc_0778^{PL38} and relevant GAGs. Amuc_0863^{GH105} was then added and the loss of the double bond of the unsaturated uronic acid was monitored at 235 nm. While linear decreasing initial rates were observed for the lower substrate concentrations, the higher concentration reactions were not reliable because of the absorbance ceiling of the spectrophotometer. Attempts to downscale or dilute the reactions did not provide data for which a reliable kinetic model could be fitted (Supplementary Figure 35). However, by isolating the highest observed initial rate for each substrate, a clear selectivity of Amuc_0863^{GH105} was observed towards CSA, followed by the three other GAGs at 36-40% relative activity (Supplementary Table 5). These results indicates that this enzyme prefers an unsaturated GlcA in the -1 subsite and a 4SGalNAc in the +1 subsite.

The products of Amuc_0778^{PL38} were then characterised in more detail using LC-MS/MS with UV detection (Extended Data 10). Time-course reactions were performed to assess the product formations. For HA, Amuc_0778^{PL38} produced dimers, tetramers and hexamers. For CSA, the sulfated disaccharide and double sulfated tetrasaccharide were produced at equal rates initially, but as the disaccharide continued to increase in concentration, the tetrasaccharide decreased, indicating degradation to disaccharides also. A different pattern was observed for CSC, where the activity was slower and the tetrasaccharide was the dominant product initially, indicating that the differing sulfation pattern in this substrate reduces activity. The activity was even slower for DS and the double sulfated tetrasaccharide was the dominant product until much later in the time-course. The only difference between CSA and DS is the presence of GlcA and IdoA, respectively, therefore based on these data, Amuc_0778^{PL38} appears to prefer GlcA over IdoA containing substrates.

Finally, we included the sulfatases with the Amuc_0778^{PL38} (with and without Amuc_0863^{GH105}) in assays with CSC and HS and no obvious activity could be seen by TLC (Supplementary Figure 37). The two GH89 enzymes were also tested against GAGs both with and without

Amuc_0778PL38 and Amuc_0863GH105, but no activity was observed (Supplementary Figure 35).

Why have CAZymes if not to use them for nutrient acquisition? Our current hypothesis is that these enzymes give AM a colonisation advantage. GAGs are an important part of the glycocalyx and would be constant presence in the lumen of the colon. Another prominent member of the human gut microbiota, *Bacteroides thetaiotaomicron*, prioritises GAGs as a nutrient source, which demonstrates its significance in the colonic environment[28]. In AM, this enzyme may be one way that AM can burrow into the mucosal layer. Interestingly, a hyaluronidase from *Streptococcus agalactiae* has been shown to dampen down the immune system and increase invasion in the context of the female reproductive system and pre-term labour[29]. This system may be comparable to the role of the GAG-active CAZymes in AM.

Growth of AM on high-mannose N-glycoproteins

When testing the growth of AM against a variety of substrates to support the biochemical work, we included glycoproteins that are decorated with mammalian complex N-glycans, as the sialidases, galactosidases, and GlcNAc'ases were observed to breakdown these substrates. The detected enzyme activities were unsurprising, as mammalian complex N-glycans have very similar glycan structures to O-glycans. For completeness, a glycoprotein with high-mannose N-glycans was also included. Unexpectedly, AM was observed to grow on the high-mannose N-glycoprotein and not the two glycoproteins with complex N-glycans (Extended Data 1). Furthermore, we also observed growth on *Saccharomyces cerevisiae* mannan (*Sc*mannan), which is an N-glycan core structure with large antennae composed of α-linked mannose and a recognised nutrient source for other gut microbes[30].

Whole cell assays using cells that had been grown on high-mannose N-glycoprotein revealed a smear on the TLC that appeared overnight (Supplementary Figure 38). This smear did not run as fast as the free high-mannose N-glycan control (suggesting a higher molecular weight), but when the sample was treated with PNGaseL, its TLC migration pattern was the same. This strongly suggests that AM is using the protein as a nutrient source and the by-product of this is high-mannose N-glycan that is still attached to a small peptide. The molecular mechanism underpinning why AM can degrade this substrate (and not other proteins or glycoproteins included in this work) is beyond the scope of this study, but it is likely attributable to a particular glycopeptidase or peptidase. However, this observation indicates a possible important cross feeding relationship AM may have with other human gut microbes. N-glycans are prevalent decorations on secreted proteins and high-mannose N-glycans are universal to eukaryotes, so microbes in the human gut would encounter this substrate from the host (secreted proteins and sloughed off dead cells) and any type of diet (animal- or plant-derived).

The whole cell assays for *Sc*mannan did not show any detectable glycobiology by TLC. All the CAZymes associated with the hydrolysis of α-linkages were tested against a panel of α-mannose glycans during the process of deciphering this growth, but there were no positive results (Supplementary Figure 9). SDS-PAGE with Coomassie staining of *Sc*mannan also showed no obvious protein in the sample (Supplementary Figure 38). To rule out very small free peptides being used as a nutrient source, the *Sc*mannan was dialysed to try and remove this, but growth was comparable to the original substrate (Extended Data 1). It may be that there is peptide attached to the *Sc*mannan that AM can access.

Starch-degrading activities

Encoded in the AM genome are two GH families that are currently characterised to have sole specificity towards starch-type polysaccharides – one GH77 and three GH13 enzymes. These enzymes were tested against several different substrates composed of α-linked glucose, however activity was only found for Amuc_1621GH77, primarily against amylose and produced oligosaccharides of one to five glucose units (Supplementary Figure 39). There was no activity against dextran or amylopectin, but potentially trace activity against pullulan. There was no

growth on these substrates (Extended Data 1). Interestingly, two of these enzymes were highlighted as important for growth on mucin using a TnSeq library[16].

Concerning GAG and starch utilisation, why the AM genome would encode enzymes specific for these substrates, when it does not use them as a nutrient source directly, remains unresolved at present. One possibility we explored is that the large mucin structures being taken up by AM also commonly contain GAG and starch contaminants. The PL38, GH105, sulfatases, GH13 and GH77 enzymes would be present internally to break these down, either for use as a nutrient source or to expel them. We attempted to test the idea that GAGs areused as a nutrient source through growths with different proportions of GAG-to-mucin, but the decrease in growth with increasing GAG concentration suggests that this theory is incorrect (Extended Data 1).

The extraordinary import system of AM
An intriguing physiological phenomenon of AM is its unusual substrate import mechanisms. In a paper describing the first genetic manipulation of AM, Davey *et al.* highlighted a possible import system that implicated pili in the import of mucin[16]. They found that mucin was accumulating in intracellular compartments, dubbed "mucinosomes". Nine CAZymes were associated with this likely import system (Mul1A) (Supplementary Table 1). Interestingly, these CAZymes were also either very highly conserved across *Akkermansia* species, predicted to be localised to the outside of the cell, and/or highlighted as important for growth on mucin. It is likely that some of these different observations are connected. Complementing the observation of mucinosomes, was the types of enzyme activity detected on the surface of AM in this report. Prominent activities included GH16 and sialidase activities, and fucosidase activities were also observed by Shuoker *et al.*[13], but other glycobiology was much lower. This suggests that most of the degradation of mucin is taking place inside the cell (Figure 6). Finally, growth of AM on human milk has also been observed [18], so there must also be a way to import human milk oligosaccharides and structurally similar GH16-derived O-glycan fragments produced on the surface of the cell. Future work on characterising this import system will be paradigm shifting in the microbiology field.

CAZymes where no activities could be identified.
In this report we have acknowledged several puzzles where activity could not be elucidated (Supplementary Table 1). Below is a discussion on these.

GH31: The second GH31, Amuc_1870^{GH31} is from subfamily 1, which has 84 characterised members and all bacterial members exhibit α-glucosidase activity. This enzyme did have activity against pNP-α-Glc, but no activity against substrates tested could be found, including those composed of α-glucose: maltose, dextran, starch, and laminarin (Supplementary Figures 7-9). A model of Amuc_1870^{GH31} was superimposed on to crystal structures of GH31 enzyme-substrate complexes with α-linked glucose disaccharides: isomaltose, nigerose, and kojibiose (Supplementary Figure 10). Only the isomaltose overlay indicated that this substrate could be accommodated, so we then tried isomaltose and pullulan (maltotriose linked via α1,6-linkages), but also found no activity. Interestingly, however, the closest characterised homologues prefered isomaltose over maltose[31, 32]. Finally, we also tested this enzyme against glucosylceramide (core of gangliosides and globosides) and trehalose, which is an unusual disaccharide produced by some organisms and is two glucose monosaccharides linked via α1,1 bond. There was no activity against either of these substrates and for the glucosylceramide this was confirmed by HPAEC (Supplementary Figure 10). AM did not grow on either maltose, starch, amylose, amylopectin, or pullulan (Extended Data 1). This enzyme is one of the unsolved puzzles (Supplementary Table 1).

GH57: Amuc_1868^{GH57} is one of the unsolved puzzles (Supplementary Table 1). We found activity on pNP-α-Glc, but none against the wide variety of carbohydrate substrates tested in this report. Interestingly, the protein sequence for Amuc_1868^{GH57} is one of the most highly

conserved across all the *Akkermansia* species considered here (>90 % identity over five species) and is always present in different strains. Adding further intrigue is that the TnSeq data previously reported by Davey *et al.* highlighted this enzyme as important for growth on mucin (Supplementary Table 1)[16]. The GH57 enzyme family has been further categorised in to four groups and the sequence of this enzyme has been previously compared to other GH57 enzymes based on sequence motifs[33]. Intriguingly, this enzyme is classified as an amylase-like protein as it is lacking a catalytic nucleophile.

GH63: Amuc_1260^{GH63} is also an unsolved puzzle (Supplementary Table 1) with activity not being identified on any substrate, including pNP-sugars. It is possible that these enzymes are involved in another function besides mucin breakdown. AM has 50 putative glycosytransferase enzymes and it is probable that at least some of these will contribute to synthesising capsular and/or exopolysaccharides. The composition of these carbohydrate structures from microorganisms is a relatively understudied area of glycobiology and the types of structures produced by different microbes are likely highly variable. Therefore, some of the 'puzzle' enzymes discussed in this report may target these structures, possibly to recycle the component sugars and facilitate cell growth and division. For example, there was no activity observed for Amuc_1868^{GH57} but it is highly conserved across *Akkermansia* species and was important for growth on mucin[16].

Amuc_1216^{GH177} and Amuc_0623^{GHnc}: These enzymes have both been associated with the breakdown of sialic acid[13, 34]. We could not find activity against any sialylated substrates or pNP-sugars in this work.

1. Ndongo, S., Armstrong, N., Raoult, D. & Fournier, P.E. Reclassification of eight Akkermansia muciniphila strains and description of Akkermansia massiliensis sp. nov. and Candidatus Akkermansia timonensis, isolated from human feces. *Sci Rep* **12**, 21747 (2022).
2. Luis, A.S. et al. Dietary pectic glycans are degraded by coordinated enzyme pathways in human colonic Bacteroides. *Nat Microbiol* **3**, 210-219 (2018).
3. Jindou, S. et al. alpha-Galactosidase Aga27A, an enzymatic component of the Clostridium josui cellulosome. *J Bacteriol* **184**, 600-604 (2002).
4. Tomasic, I.B., Metcalf, M.C., Guce, A.I., Clark, N.E. & Garman, S.C. Interconversion of the specificities of human lysosomal enzymes associated with Fabry and Schindler diseases. *J Biol Chem* **285**, 21560-21566 (2010).
5. Olsson, M.L. & Clausen, H. Modifying the red cell surface: towards an ABO-universal blood supply. *Br J Haematol* **140**, 3-12 (2008).
6. Okuyama, M. et al. Efficient synthesis of alpha-galactosyl oligosaccharides using a mutant Bacteroides thetaiotaomicron retaining alpha-galactosidase (BtGH97b). *FEBS J* **284**, 766-783 (2017).
7. Galili, U., Clark, M.R., Shohet, S.B., Buehler, J. & Macher, B.A. Evolutionary relationship between the natural anti-Gal antibody and the Gal alpha 1----3Gal epitope in primates. *Proc Natl Acad Sci U S A* **84**, 1369-1373 (1987).
8. Grondin, J.M. et al. Diverse modes of galacto-specific carbohydrate recognition by a family 31 glycoside hydrolase from Clostridium perfringens. *PLoS One* **12**, e0171606 (2017).
9. Lombard, V., Golaconda Ramulu, H., Drula, E., Coutinho, P.M. & Henrissat, B. The carbohydrate-active enzymes database (CAZy) in 2013. *Nucleic Acids Res* **42**, D490-495 (2014).
10. Fujita, M. et al. Glycoside hydrolase family 89 alpha-N-acetylglucosaminidase from Clostridium perfringens specifically acts on GlcNAc alpha1,4Gal beta1R at the non-reducing terminus of O-glycans in gastric mucin. *J Biol Chem* **286**, 6479-6489 (2011).
11. Ficko-Blean, E. & Boraston, A.B. Structural analysis of a bacterial exo-alpha-D-N-acetylglucosaminidase in complex with an unusual disaccharide found in class III mucin. *Glycobiology* **22**, 590-595 (2012).
12. Birrane, G. et al. Structural characterization of the alpha-N-acetylglucosaminidase, a key enzyme in the pathogenesis of Sanfilippo syndrome B. *J Struct Biol* **205**, 65-71 (2019).
13. Shuoker, B. et al. Sialidases and fucosidases of Akkermansia muciniphila are crucial for growth on mucin and nutrient sharing with mucus-associated gut bacteria. *Nat Commun* **14**, 1833 (2023).
14. Jajosky, R.P. et al. ABO blood group antigens and differential glycan expression: Perspective on the evolution of common human enzyme deficiencies. *iScience* **26**, 105798 (2023).
15. Rueda, R., Maldonado, J., Narbona, E. & Gil, A. Neonatal dietary gangliosides. *Early Hum Dev* **53 Suppl**, S135-147 (1998).
16. Davey, L.E. et al. A genetic system for Akkermansia muciniphila reveals a role for mucin foraging in gut colonization and host sterol biosynthesis gene expression. *Nat Microbiol* **8**, 1450-1467 (2023).
17. Padilla, L. et al. Mechanism of 2'-fucosyllactose degradation by human-associated Akkermansia. *J Bacteriol* **206**, e0033423 (2024).
18. Kostopoulos, I. et al. Akkermansia muciniphila uses human milk oligosaccharides to thrive in the early life conditions in vitro. *Sci Rep* **10**, 14330 (2020).
19. Kosciow, K. & Deppenmeier, U. Characterization of three novel beta-galactosidases from Akkermansia muciniphila involved in mucin degradation. *Int J Biol Macromol* **149**, 331-340 (2020).
20. Guo, B.S. et al. Cloning, purification and biochemical characterisation of a GH35 beta-1,3/beta-1,6-galactosidase from the mucin-degrading gut bacterium Akkermansia muciniphila. *Glycoconj J* **35**, 255-263 (2018).

21. Kosciow, K. & Deppenmeier, U. Characterization of a phospholipid-regulated beta-galactosidase from Akkermansia muciniphila involved in mucin degradation. *Microbiologyopen* **8**, e00796 (2019).
22. Wang, M. et al. Cloning, purification and biochemical characterization of two beta-N-acetylhexosaminidases from the mucin-degrading gut bacterium Akkermansia muciniphila. *Carbohydr Res* **457**, 1-7 (2018).
23. Chen, X., Li, M., Wang, Y., Tang, R. & Zhang, M. Biochemical characteristics and crystallographic evidence for substrate-assisted catalysis of a beta-N-acetylhexosaminidase in Akkermansia muciniphila. *Biochem Biophys Res Commun* **517**, 29-35 (2019).
24. Perna, V.N., Barrett, K., Meyer, A.S. & Zeuner, B. Substrate specificity and transglycosylation capacity of alpha-L-fucosidases across GH29 assessed by bioinformatics-assisted selection of functional diversity. *Glycobiology* **33**, 396-410 (2023).
25. Pilgaard, B. et al. Discovery of a Novel Glucuronan Lyase System in Trichoderma parareesei. *Appl Environ Microbiol* **88**, e0181921 (2022).
26. Ronne, M.E. et al. Three alginate lyases provide a new gut Bacteroides ovatus isolate with the ability to grow on alginate. *Appl Environ Microbiol* **89**, e0118523 (2023).
27. Itoh, T., Ochiai, A., Mikami, B., Hashimoto, W. & Murata, K. A novel glycoside hydrolase family 105: the structure of family 105 unsaturated rhamnogalacturonyl hydrolase complexed with a disaccharide in comparison with family 88 enzyme complexed with the disaccharide. *J Mol Biol* **360**, 573-585 (2006).
28. Martens, E.C., Chiang, H.C. & Gordon, J.I. Mucosal glycan foraging enhances fitness and transmission of a saccharolytic human gut bacterial symbiont. *Cell Host Microbe* **4**, 447-457 (2008).
29. Coleman, M. et al. Hyaluronidase Impairs Neutrophil Function and Promotes Group B Streptococcus Invasion and Preterm Labor in Nonhuman Primates. *mBio* **12** (2021).
30. Cuskin, F. et al. Human gut Bacteroidetes can utilize yeast mannan through a selfish mechanism. *Nature* **517**, 165-169 (2015).
31. Tan, K. et al. Novel alpha-glucosidase from human gut microbiome: substrate specificities and their switch. *FASEB J* **24**, 3939-3949 (2010).
32. Chaudet, M.M., Allen, J.L. & Rose, D.R. Expression and purification of two Family GH31 alpha-glucosidases from Bacteroides thetaiotaomicron. *Protein Expr Purif* **86**, 135-141 (2012).
33. Janecek, S. & Blesak, K. Sequence-structural features and evolutionary relationships of family GH57 alpha-amylases and their putative alpha-amylase-like homologues. *Protein J* **30**, 429-435 (2011).
34. Ishikura, H., Arakawa, S., Nakajima, T., Tsuchida, N. & Ishikawa, I. Cloning of the Tannerella forsythensis (Bacteroides forsythus) siaHI gene and purification of the sialidase enzyme. *J Med Microbiol* **52**, 1101-1107 (2003).

Supplementary Results

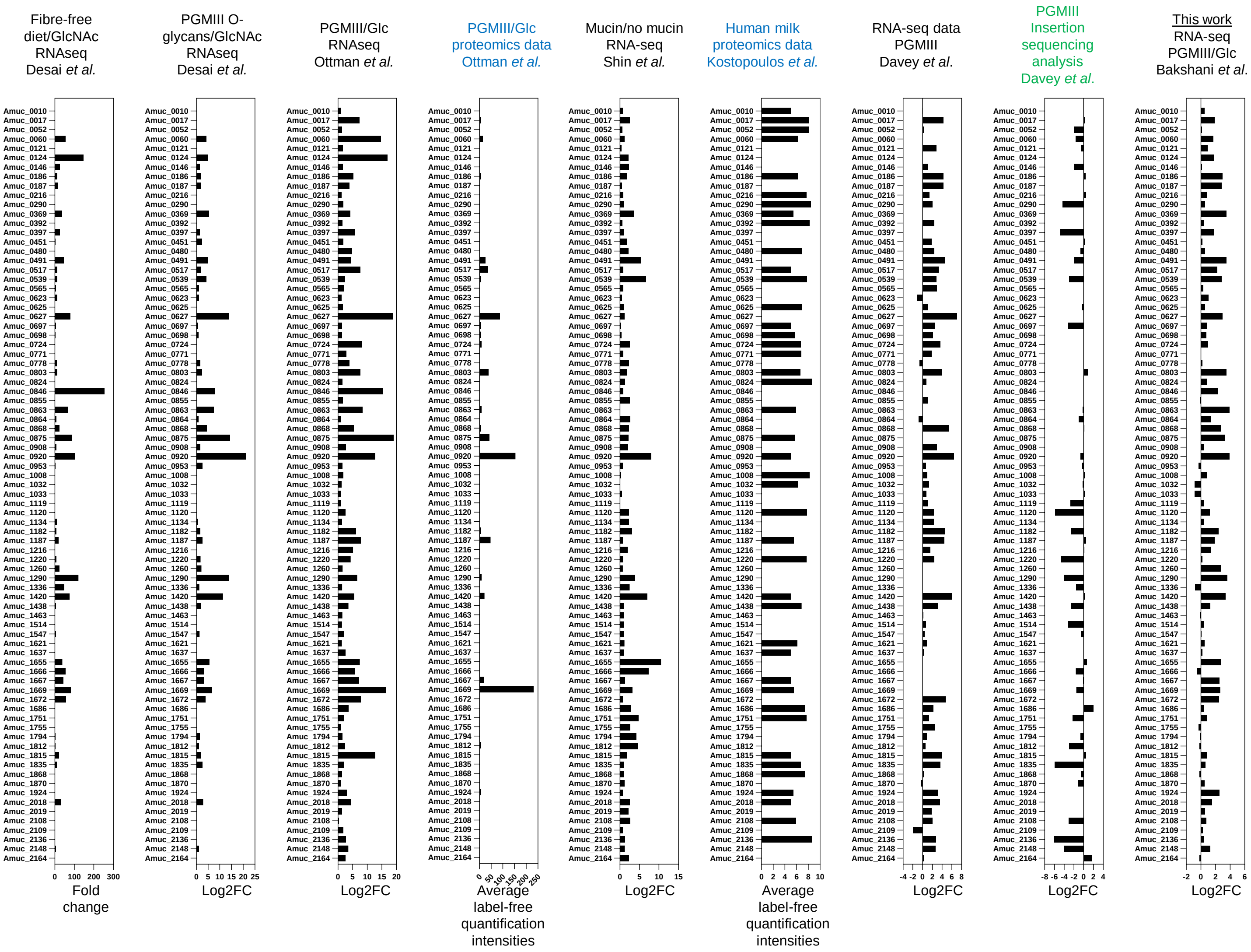

Supplementary Figure 1│Transcriptomic, proteomic and Tnseq data of AM from this work and previous publications. The bar charts show the data from different labs and techniques that assess gene upregulation, protein expression, or the reduction in growth when a gene is disrupted. Black, blue, and green indicate RNAseq, proteomics, and INSeq data, respectively.

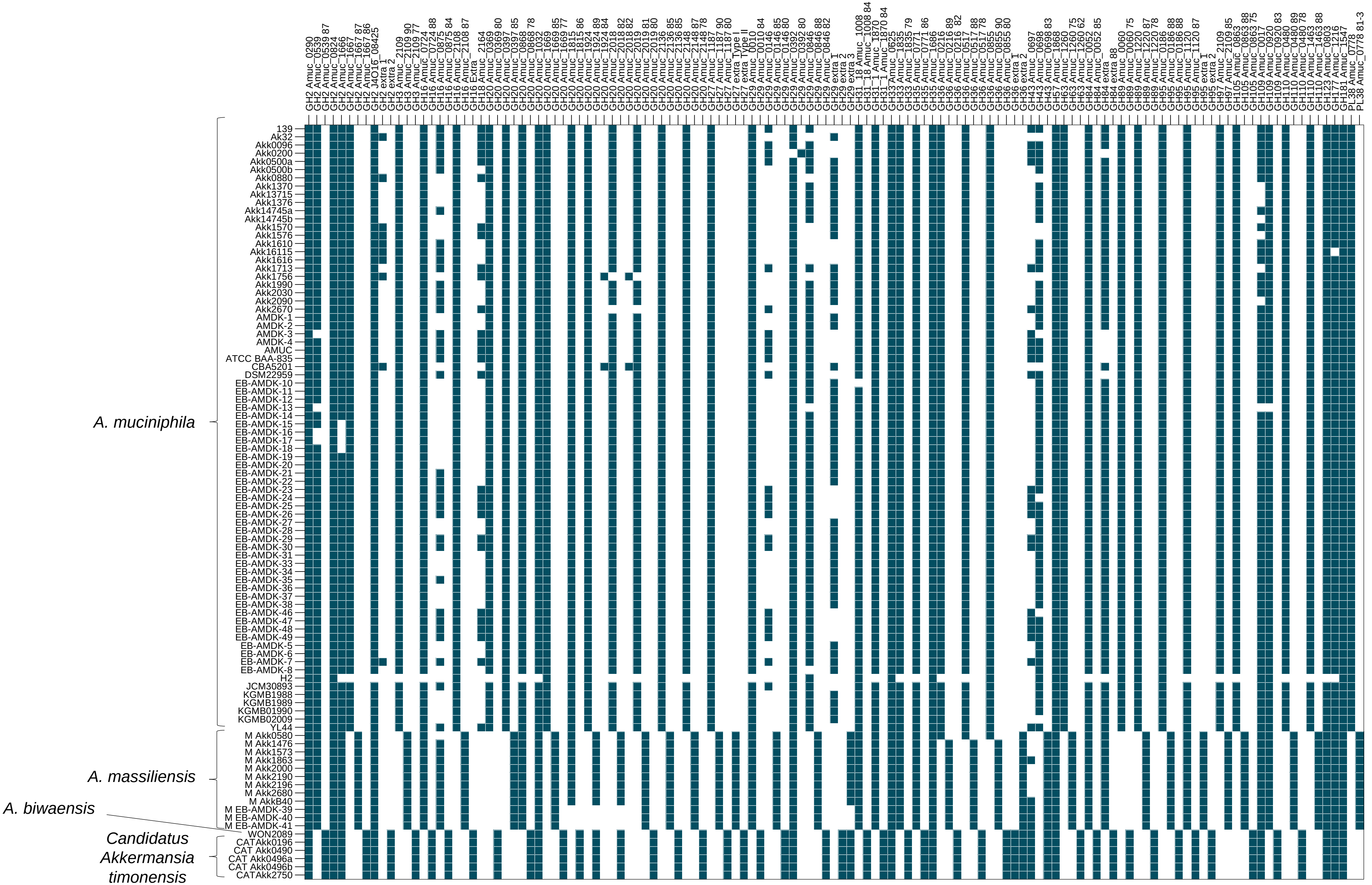

Supplementary Figure 2│ Presence of homologues of the putative CAZymes from ATCC BAA-835 across other *A. muciniphila* strains and *Akkermansia* species. The heat map shows the presence of homologues to the CAZymes in ATCC BAA-835 (dark teal). When the homologue is <90 % identical, <80 % identical, and <70 % identical then these are placed in a separate columns. For instance, the homologues for Amuc_0290^{GH2} are all >90 % across strains and species, whereas the homologues for Amuc_1667^{GH2} all have lower percentage identity across the species but >90 % conservation within strains. The y axis is split up according to species and the x axis is in numerical GH order.

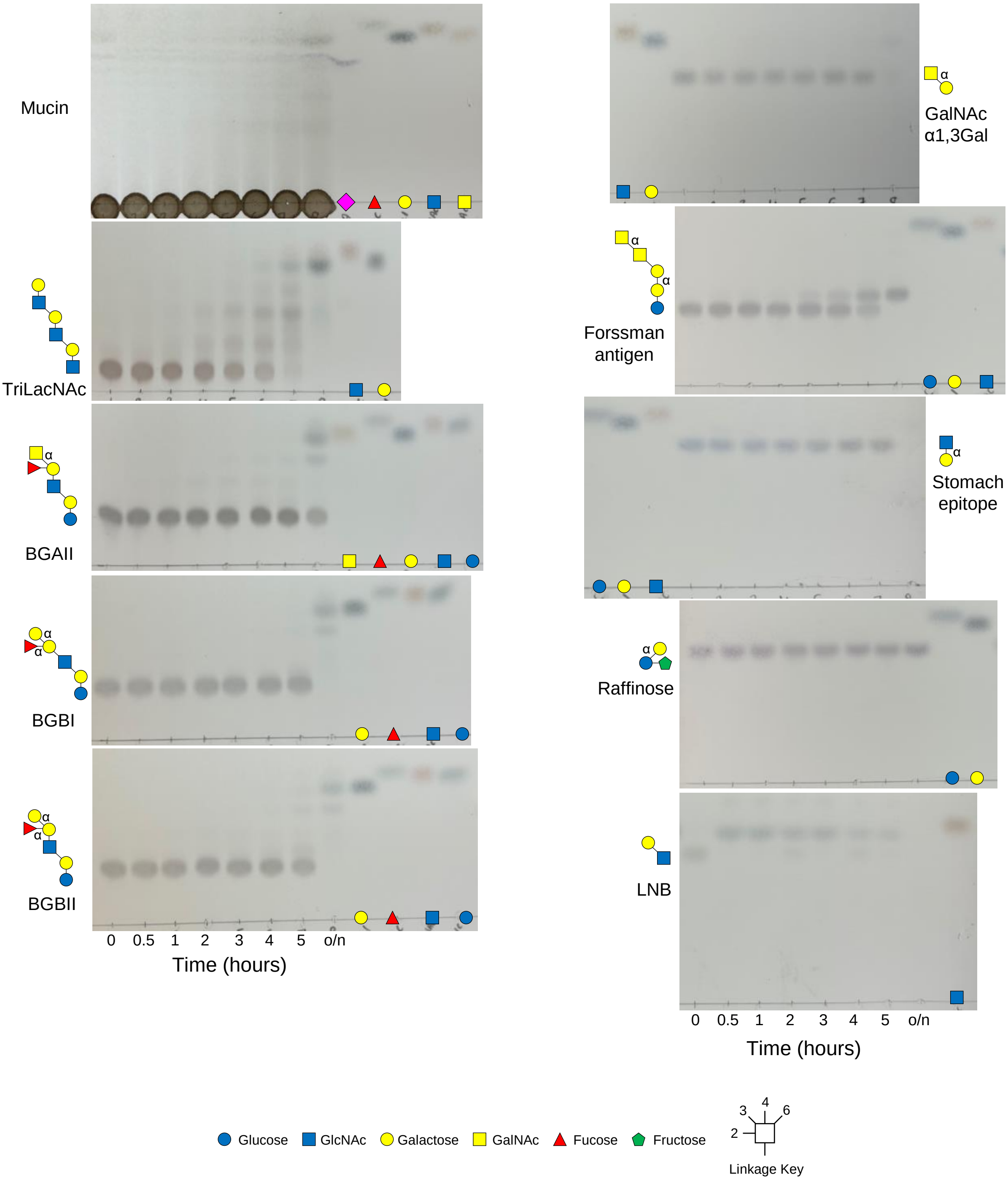

Supplementary Figure 3│Whole cell assay #1. Thin layer chromatography results of whole cell assays of PGM III-grown cells against different substrates. The reaction was carried out at 37 °C, a sample removed at different time points, and boiled to stop enzyme activities. Monosaccharide standards are shown in the right lanes.

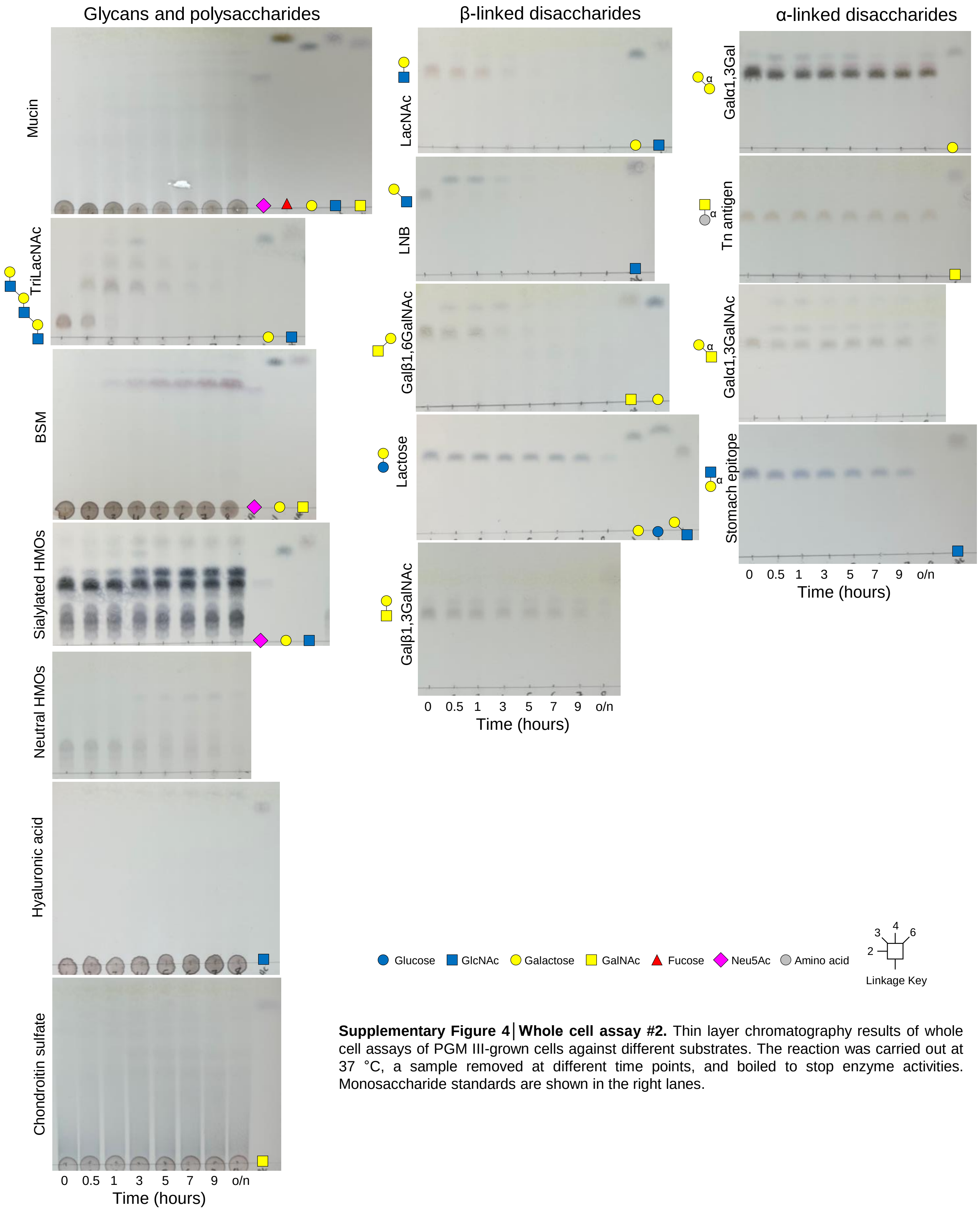

Supplementary Figure 4│Whole cell assay #2. Thin layer chromatography results of whole cell assays of PGM III-grown cells against different substrates. The reaction was carried out at 37 °C, a sample removed at different time points, and boiled to stop enzyme activities. Monosaccharide standards are shown in the right lanes.

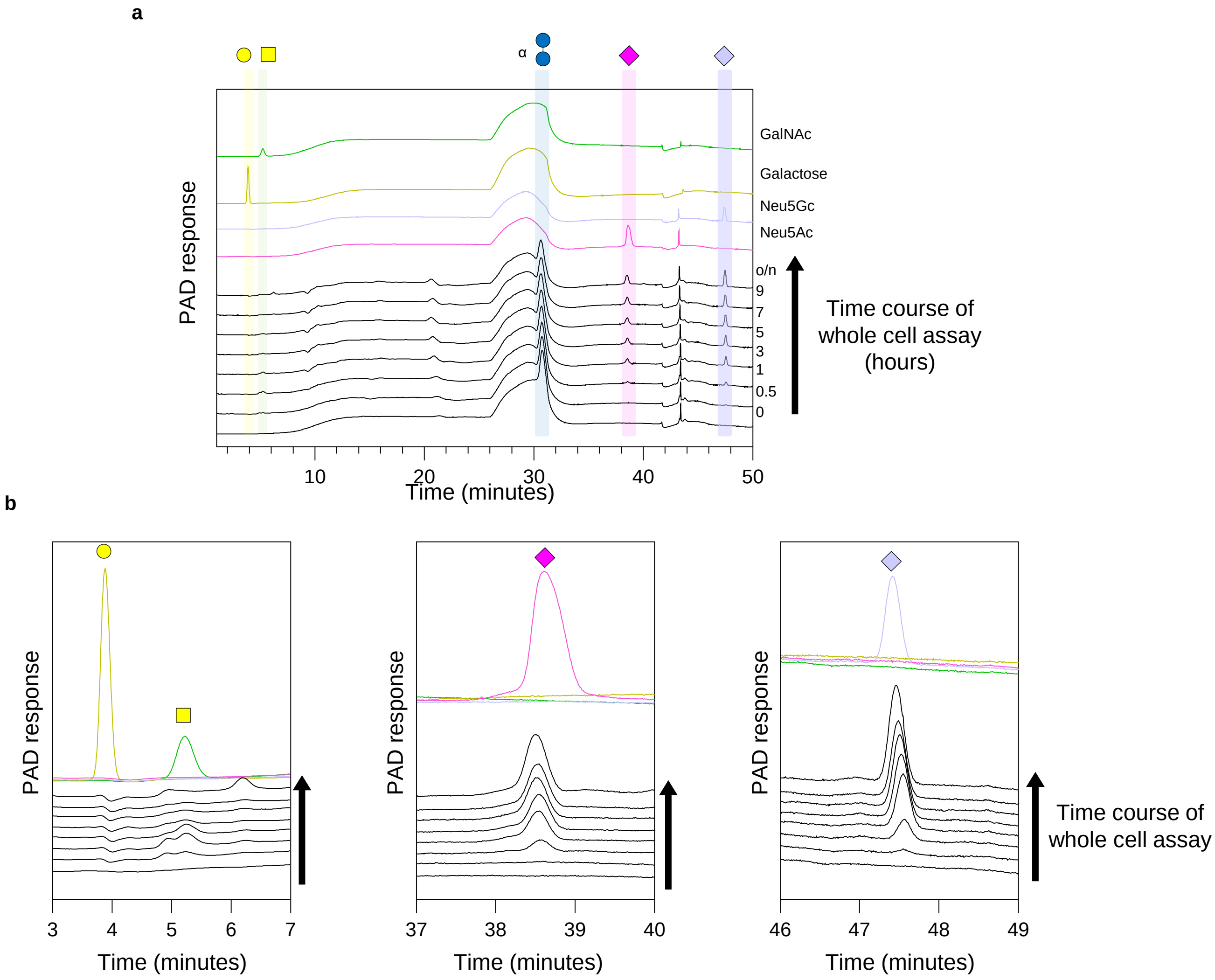

Supplementary Figure 5│HPAEC-PAD of BSM whole cell assays time points. Samples from Supplementary Figure 9 of PGM III-grown whole cell assays against BSM were analysed by HPAEC-PAD. **a,** The full chromatograms are shown alongside standards of the different expected monosaccharides (colourful lines). **b,** Certain sections of the chromatograms are shown in more detail and stacked to show the increase in sialic acids produced over time.

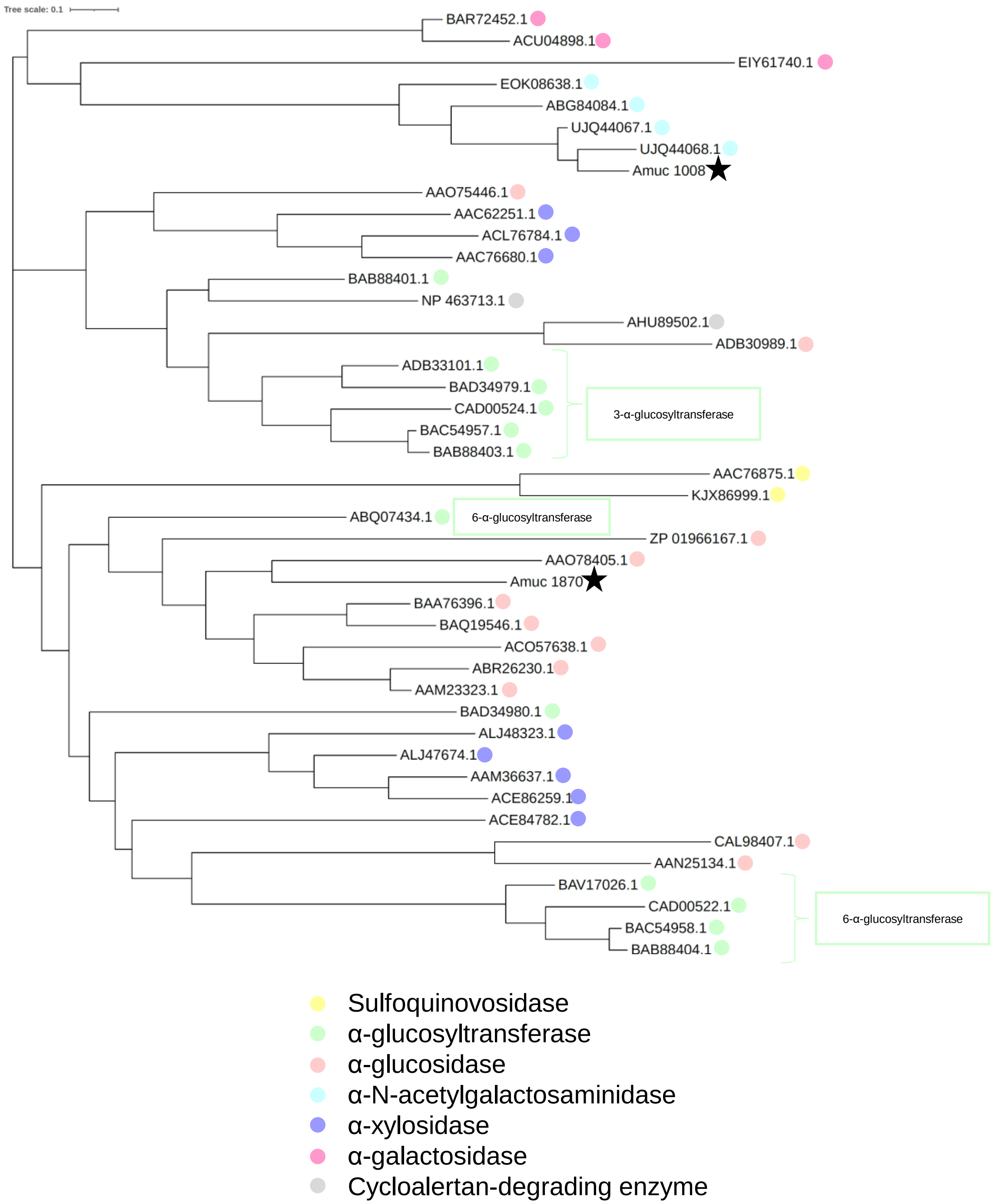

Supplementary Figure 6│Phylogenetic tree of characterised GH31 family members with those from *A. muciniphila* ATCC BAA-835. The sequences of the GH31 family members with reported activities (CAZy database) and the ones from *A. muciniphila* ATCC BAA-835 were compared as described in the methods. The different specificities are indicated by different colours and the *A. muciniphila* ATCC BAA-835 enzymes are highlighted by black stars. Some more specific information about activity is supplied where possible. The enzymes are represented by their accession numbers of locus tags. Different specificities cluster in this analysis and the observed activity of the two *A. muciniphila* ATCC BAA-835 enzymes correlates to where they cluster on the tree.

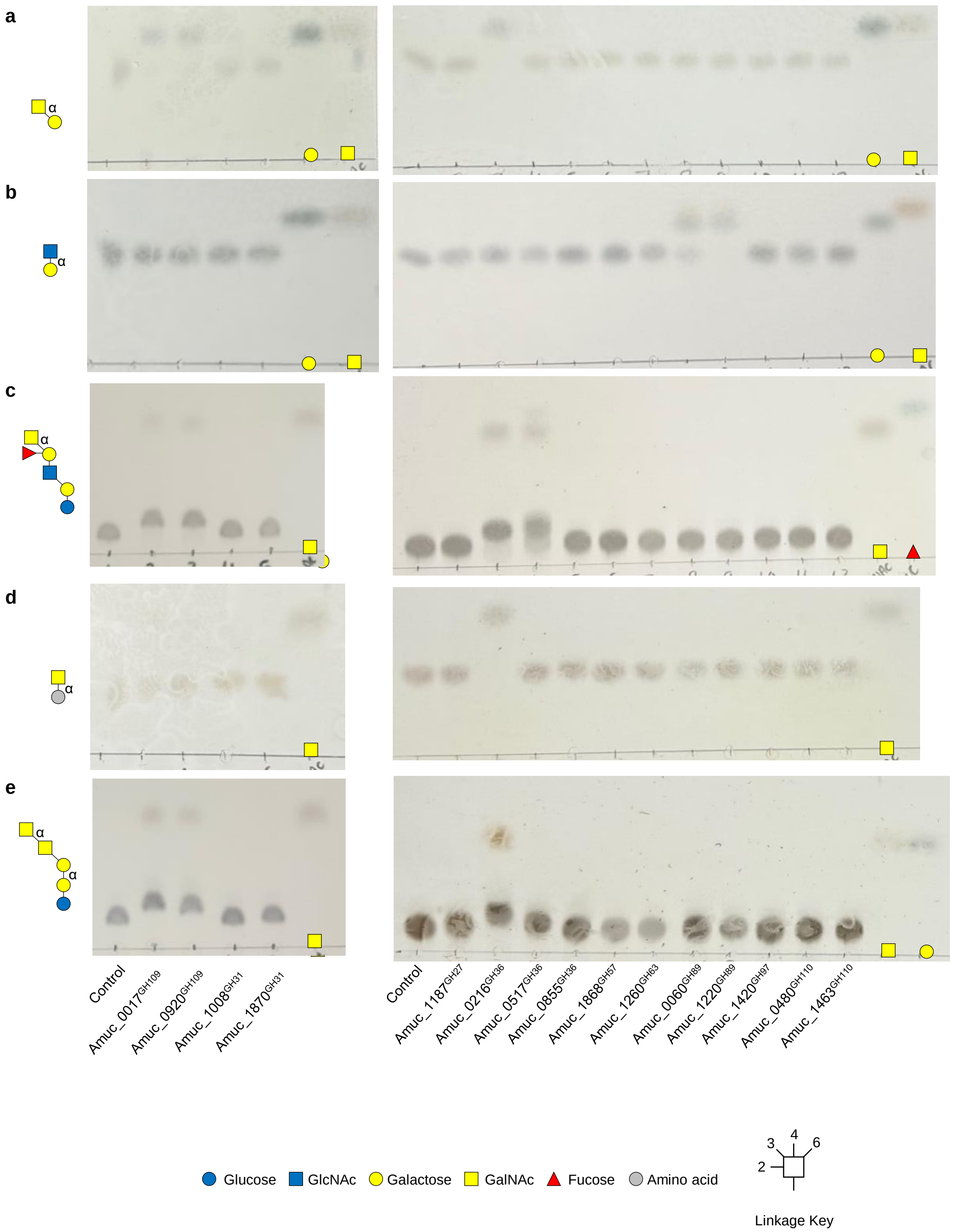

Supplementary Figure 7│Activity of GH enzymes from *A. muciniphila* BAA-835 against defined oligosaccharides capped with α-GalNAc or α-GlcNAc. a, GalNAcα1,3Gal. **b**, GlcNAcα1,4Gal. **c**, Blood group A II. **d**, Tn antigen. **e**, Forssman antigen. Standards have also been included on the TLCs on the right. Enzyme assays were carried out at a final substrate concentration of 1 mM, pH 7, 37 °C, overnight, and with 1 μM enzyme.

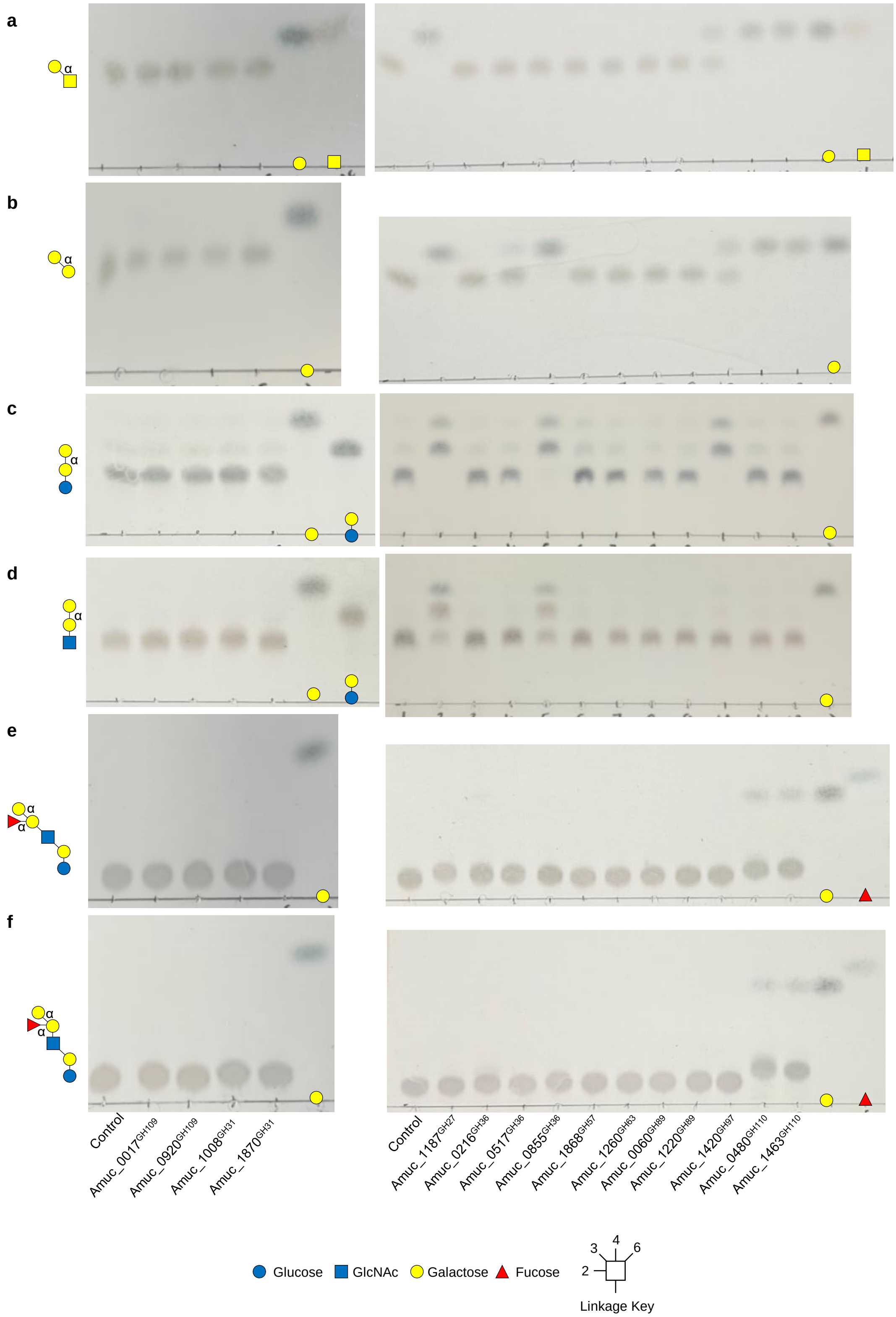

Supplementary Figure 8│Activity of GH enzymes from *A. muciniphila* BAA-835 against defined oligosaccharides capped with α-GalNAc or α-GlcNAc. a, Galα1,3GalNAc. **b**, Galα1,3Gal. **c**, globotriose. **d**, P1 antigen. **e**, Blood group B I. **f**, Blood group B II. Standards have also been included on the TLCs on the right. Enzyme assays were carried out at a final substrate concentration of 1 mM, pH 7, 37 °C, overnight, and with 1 μM enzyme.

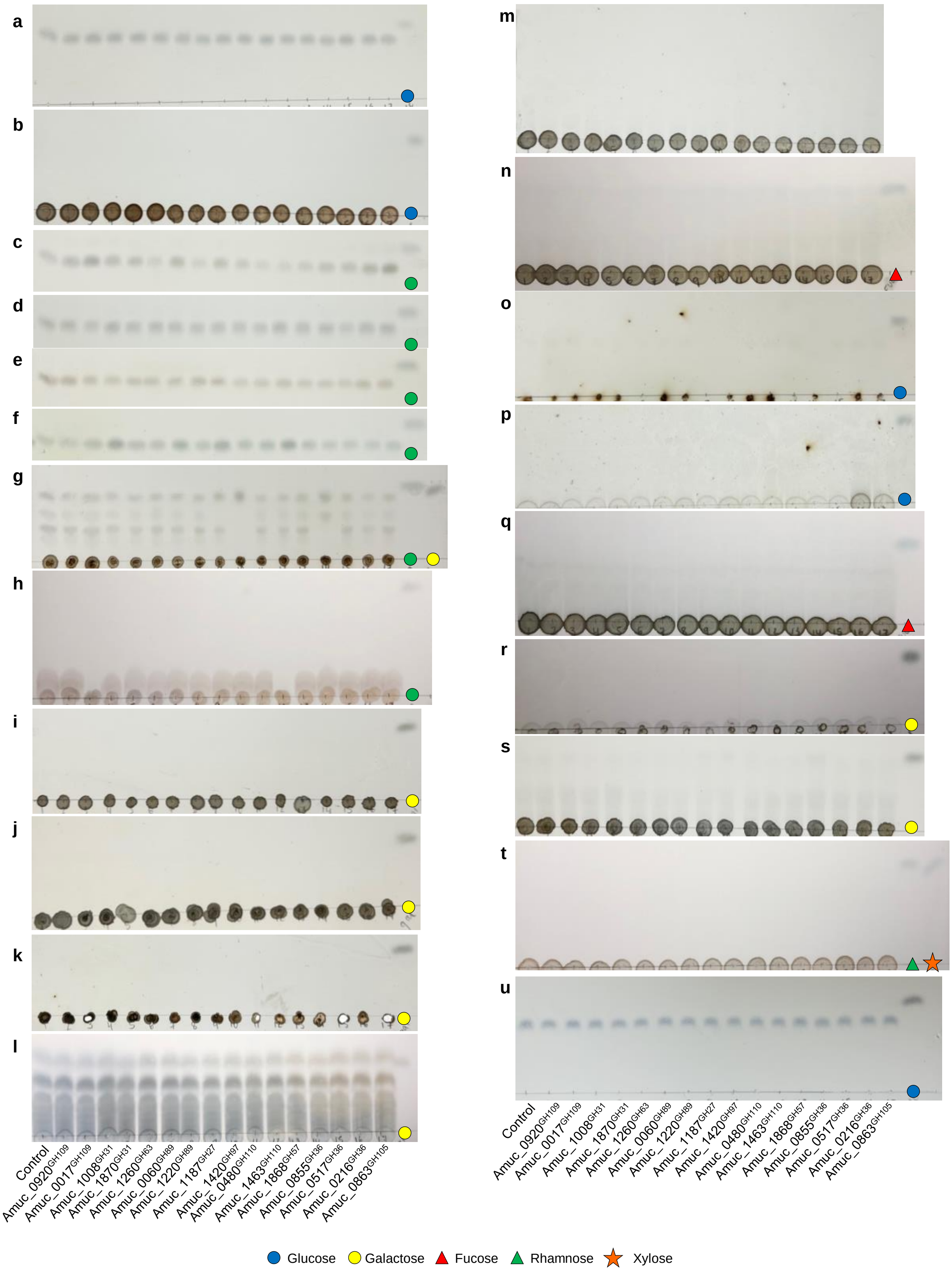

Supplementary Figure 9│Activity of GH enzymes from *A. muciniphila* BAA-835 against a wide variety of substrates. a, maltose. **b**, dextran. **c**, α1,2-mannobiose. **d**, α1,3-mannobiose **e**, α1,4-mannobiose **f**, α1,6-mannobiose. **g,** galactomannan. **h,** RNaseB (high-mannose N-glycans). **i,** L-carrageenan. **j,** K-carrageenan. **k,** I-carrageenan. **l,** porphyran (L). **m,** ulvan. **n,** fucan. **o,** starch. **p,** laminarin. **q,** fucoidan. **r,** agarose. **s,** porphyran (W). **t,** ulvan (entero). **u,** isomaltose. Standards have also been included on the TLCs. Enzyme assays were carried out at a final substrate concentration of 1 mM, pH 7, 37 °C, overnight, and with 1 μM enzyme.

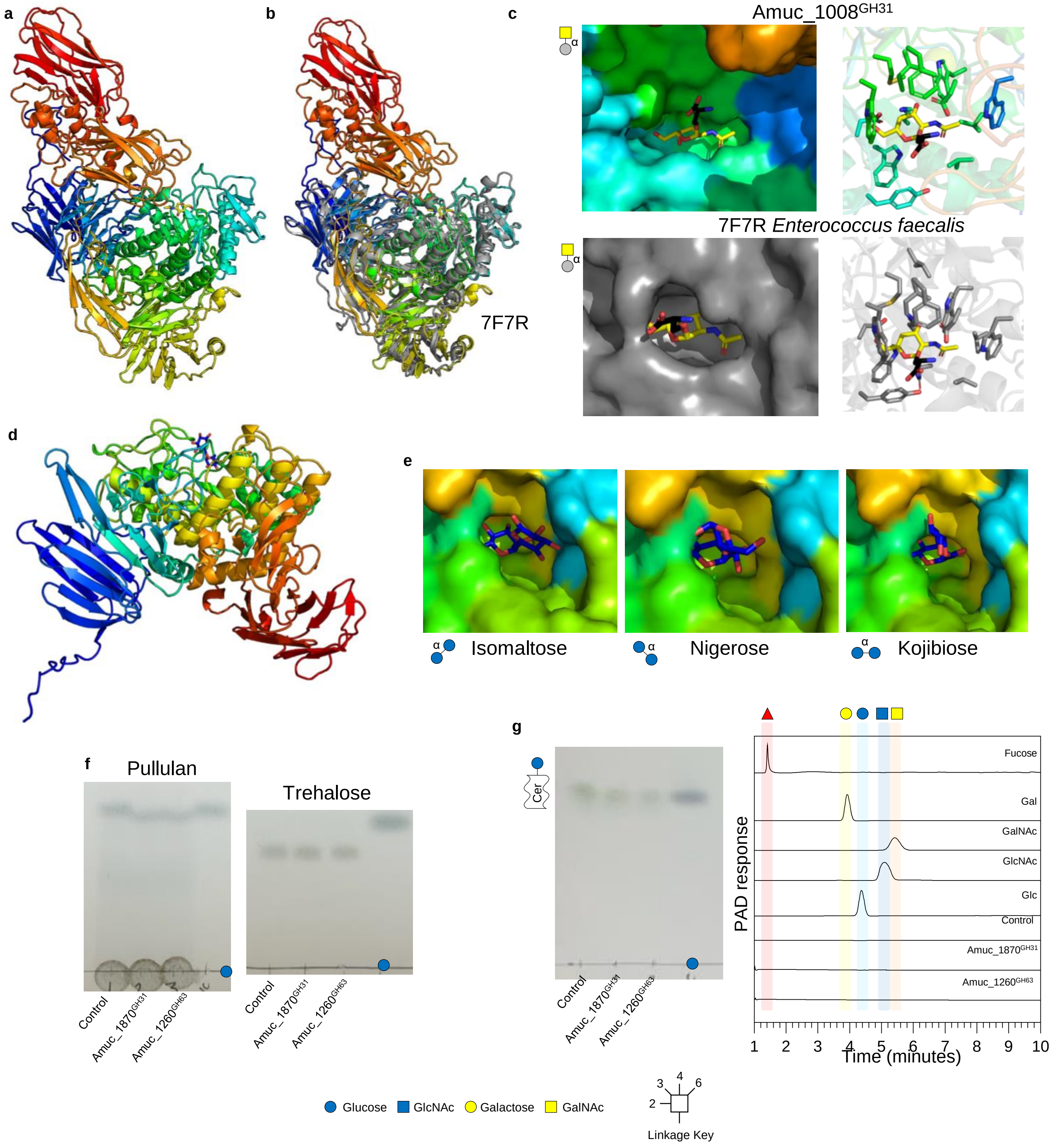

Supplementary Figure 10│GH31 family members from *A. muciniphila* ATCC BAA-835. a. Amuc_1008^{GH31} cartoon model coloured rainbow blue-red from the N- to C-terminus, respectively. GalNAc (yellow) from 7F7Q was overlaid into the active site. **b.** The cartoon of Amuc_1008^{GH31} (rainbow) overlaid with the *E. faecalis* GH31 (grey) with the same specificity showing that they overlay well in terms of secondary structures, but the *A. muciniphila* enzyme has additional modules. **c.** Top: Amuc_1008^{GH31} active site surface overlaid with Tn-antigen from 7F7R *Enterococcus faecalis* GH31_18. Bottom: active site of 7F7R for comparison. Left and right panels show the surface of the enzymes and residues interacting with the sugar in the -1 subsite as sticks, respectively. **d.** Amuc_1187^{GH31} cartoon model coloured rainbow blue-red from the N- to C-terminus, respectively. Isomaltose (blue) from 3MKK was overlaid into the active site. **e.** Amuc_1187^{GH31} active site surface overlaid with α-linked glucose disaccharides from different crystal structures. From left to right: Isomaltose from 3MKK, Nigerose from 7WJC, and Kojibiose from 7WJF. Only the isomaltose was not making obvious steric clashes with the predicted protein structure. **f.** Activity against pullulan and trehalose. Isomaltose reactions are in Supplementary Figure 16. **g.** Activity against glucosylceramide. Left panel is the TLC and right panel are the assays run on the HPAEC alongside standards. Standards have also been included on the TLCs. Enzyme assays were carried out at a final substrate concentration of 1 mM, pH 7, 37 °C, overnight, and with 1 μM enzyme.

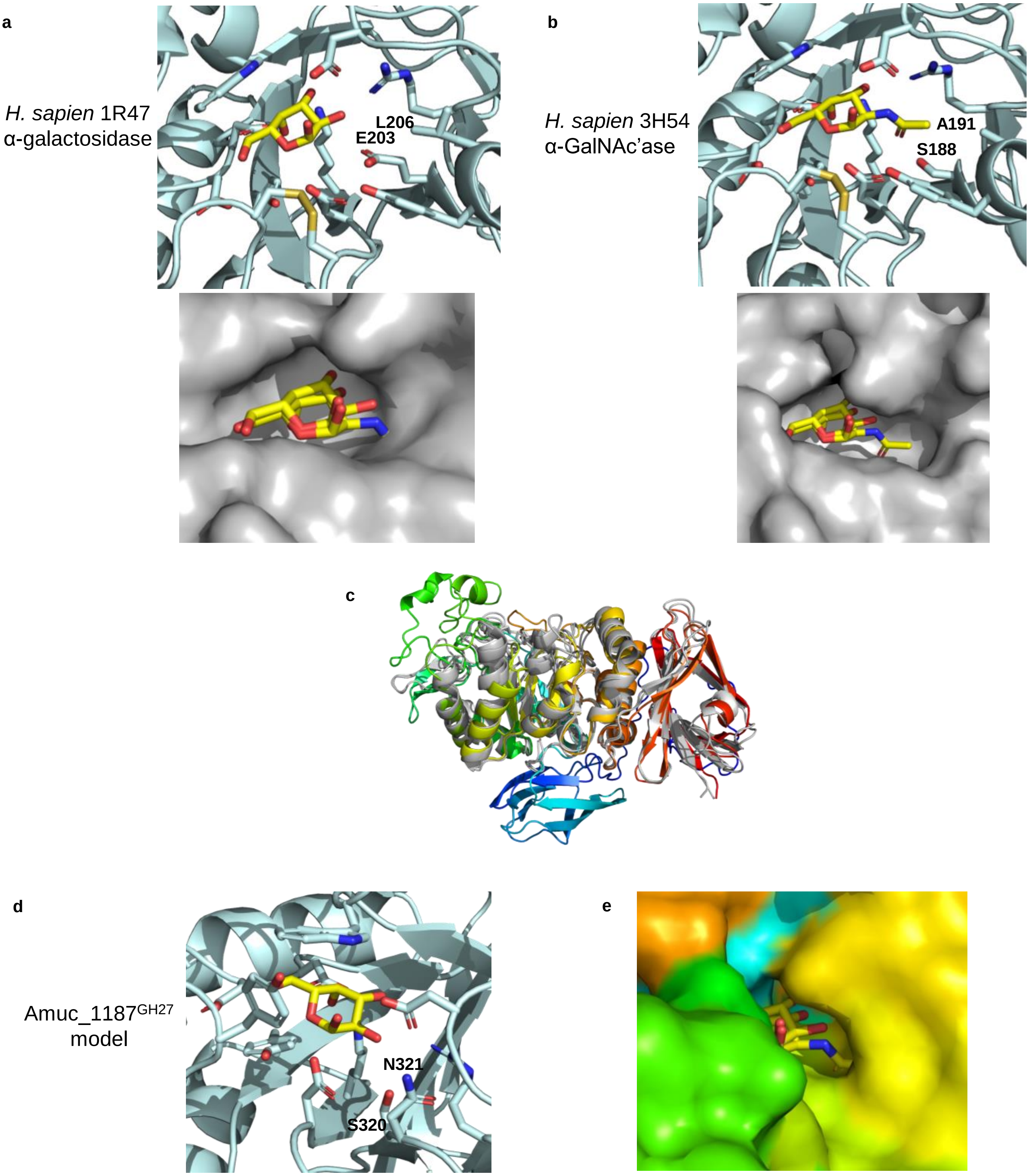

Supplementary Figure 11│Models of the GH27 family member from *A. muciniphila* ATCC BAA-835. a. Human GH27 α-galactosidase with galactose in the -1 subsite and **b.** Human GH27 α-GalNAc'ase with GalNAc in the -1 subsite. The residues coordinating these -1 sugars differ only in the residues that have been labelled. The larger residues coordinate the galactose in 1R47 and the smaller residues allow the accommodation of GalNAc in 3H54. **c.** An overlay of Amuc_1187^{GH27} (rainbow, N- to C-termini are blue-red, respectively) with the two *H. sapien* enzymes (grey) show that the overall fold is largely similar. **d.** A model of Amuc_1187^{GH27} with the ligand from 1R47 and the two equivalent residues. These are relatively large residues, so the enzyme has specificity for galactose rather than GalNAc. **e.** A surface representation of the model of Amuc_1187^{GH27} to show the pocket for galactose more clearly.

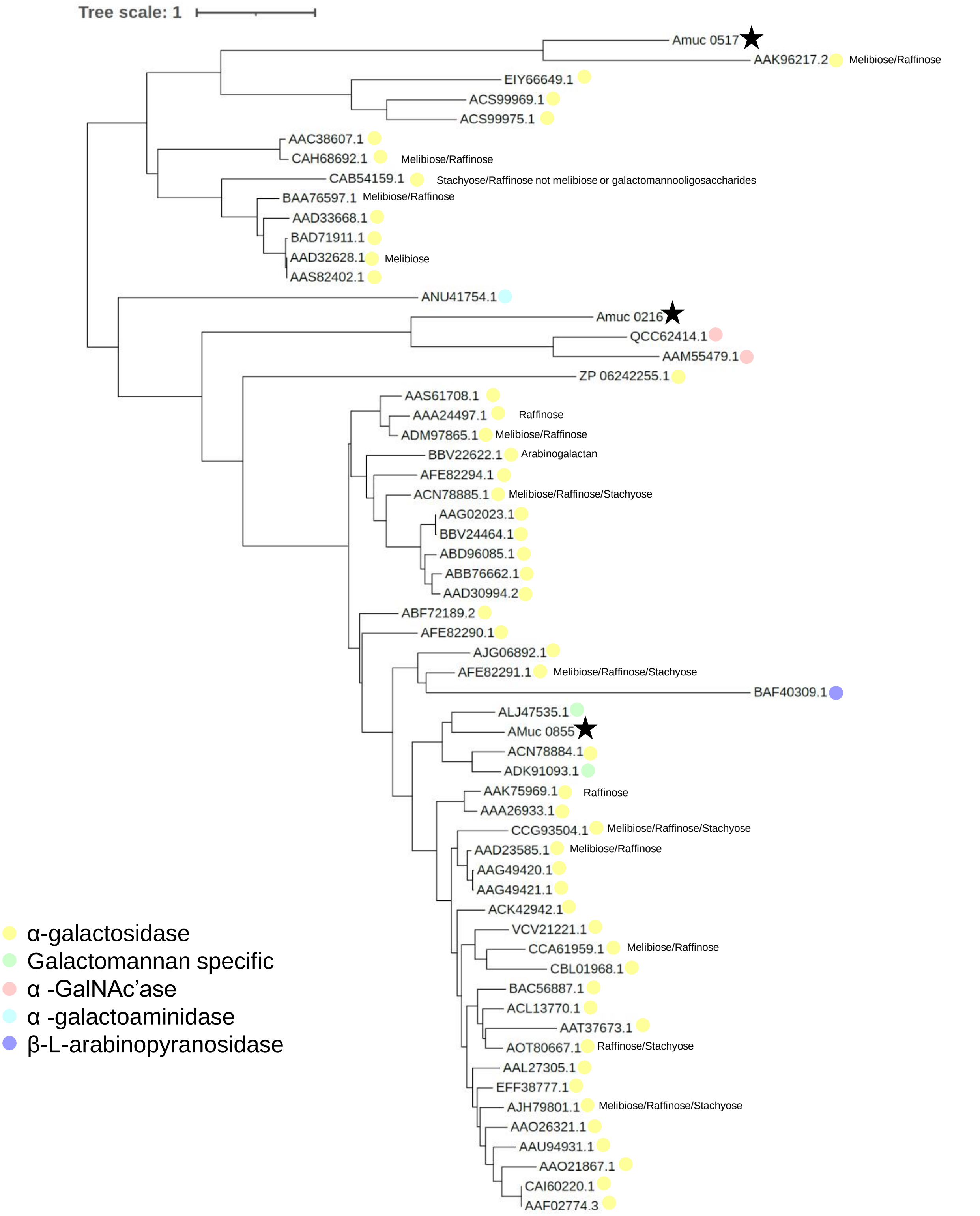

Supplementary Figure 12│Phylogenetic tree of characterised GH36 family members with those from *A. muciniphila* ATCC BAA-835. The sequences of the GH36 family members with reported activities (CAZy database) and the ones from *A. muciniphila* ATCC BAA-835 were compared as described in the methods. The different specificities are indicated by different colours and the *A. muciniphila* ATCC BAA-835 enzymes are highlighted by black stars. The enzymes are represented by their accession numbers of locus tags.

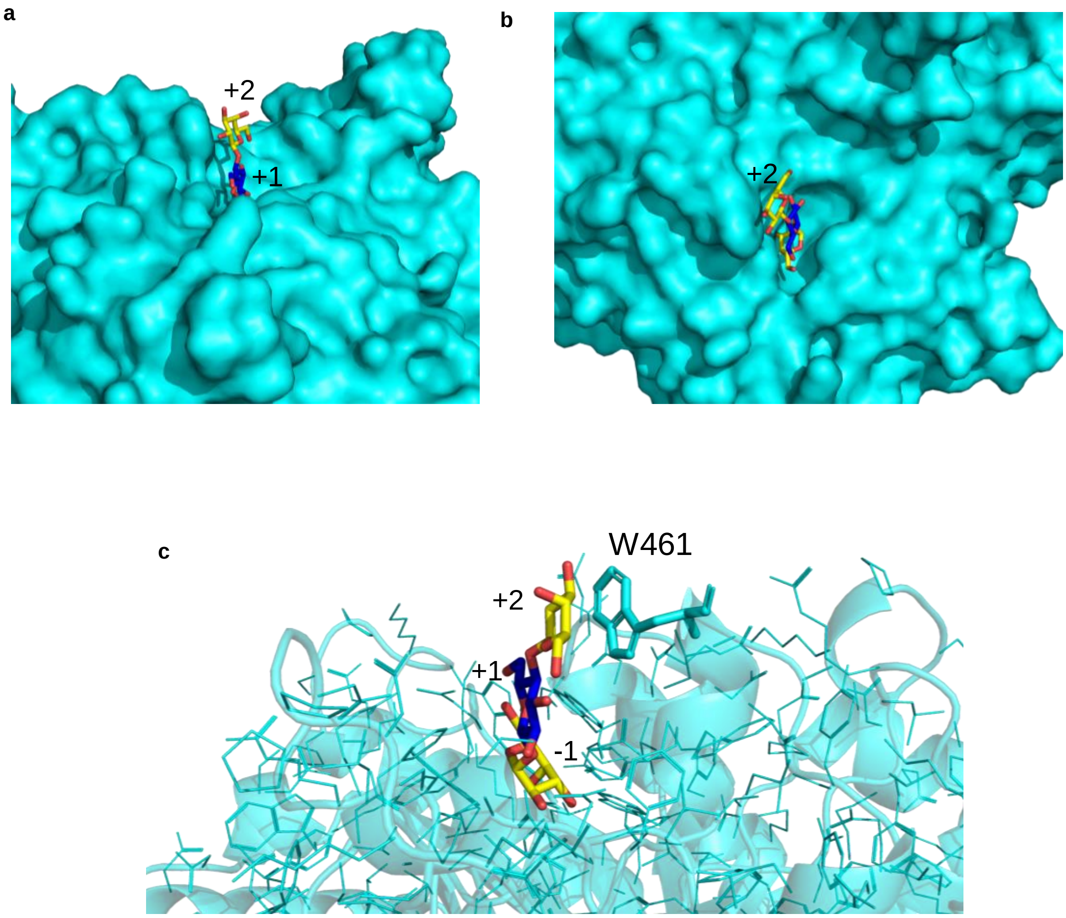

Supplementary Figure 13│The structural model of the Amuc_1420^{GH97}. a, The active site of the model is viewed in surface mode from the side. Overlaid into the active site is a trisaccharide (lactose that also has an α-linked galactose to the Glc through a 1,1-linkage) from the *Bacteroides thetaiotaomicron* BT1871 structure. b, same structure looking down into the active site. c, a close up of the active site, showing the secondary structure as cartoon, the amino acid side chains as lines, apart from one tryptophan that may interact with the sugar in the +2 subsite.

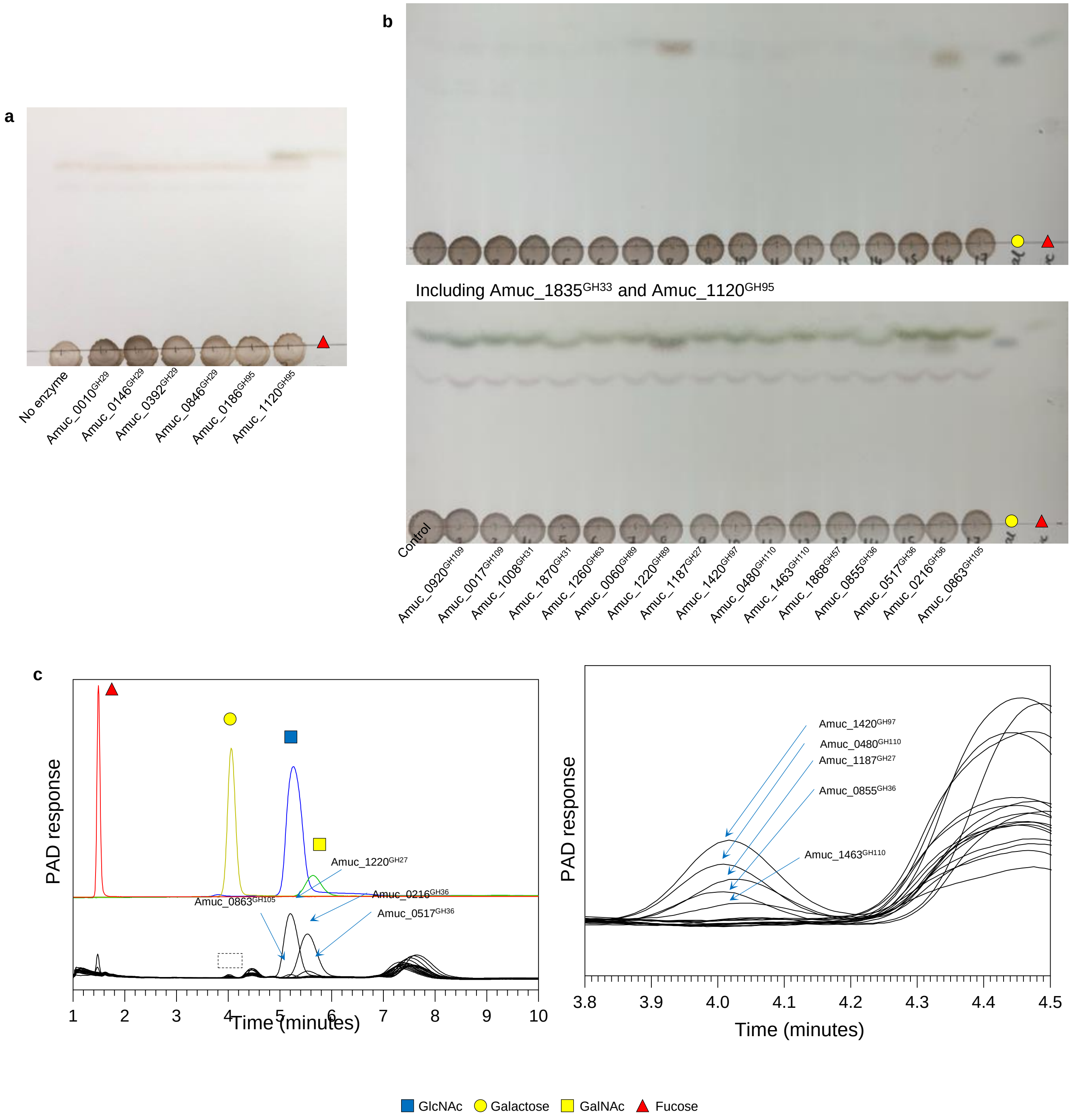

Supplementary Figure 14│Degradation of PGM III with fucosidases and alpha-linkage CAZymes from *A. muciniphila* BAA-835 and analysis by thin layer chromatography. Enzyme assays were carried out pH 7, 37 °C, overnight, and with 1 µM enzymes. **a,** The activity of GH29 and GH95 family members against PGM III. **b**, The activity of GH enzymes associated with the hydrolysis of alpha-linkage removal. Top panel is without enzyme pre-treatment included and the HPAEC-PAD for these samples is presented in ‘c’. The bottom panel are the same assays with Amuc_1835^{GH33} and Amuc_1120^{GH95} included and the HPAEC-PAD for these samples is presented in Figure 2.

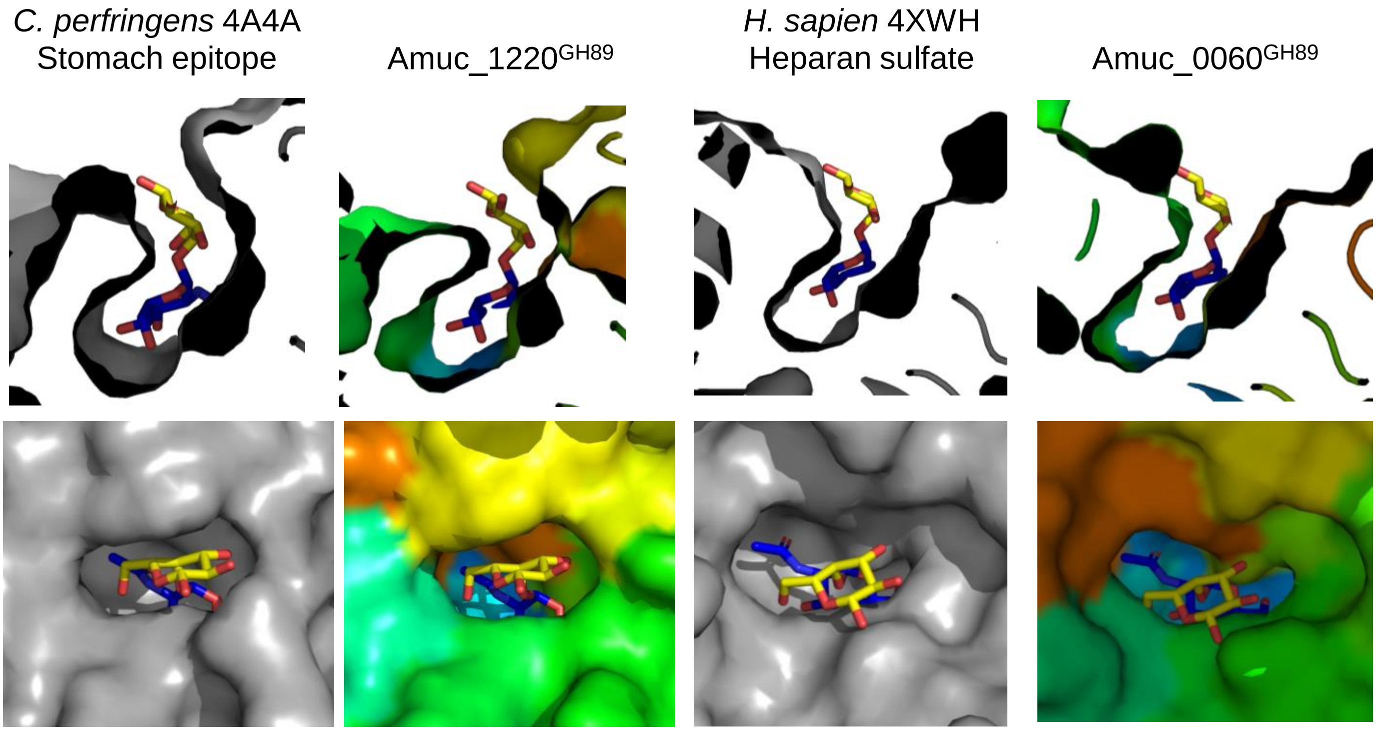

Supplementary Figure 15│Structural insights into GH89 enzymes from *A. muciniphila* ATCC BAA-835. Two crystal structures (grey) with different substrate preferences from the GH89 family were compared to models of the two GH89 enzymes analysed in this work (rainbow). The *C. perfringens* 4A4A structure was crystallised with GlcNAcα1,4Gal in its active site and this was overlaid into the structure from *H. sapien* 4XWH and the two models. GlcNAc and Gal are blue and yellow, respectively. The top panels are cross sections through the active site to show how this disaccharide sits in there and the bottom panels are looking down at the entrance of the active site. The active site of the *C. perfringens* 4A4A structure packs tightly around the sugars in the -1 and +1 subsites, which has a considerable curve in its structure. The conformation of the Amuc_1220^{GH89} active site pocket is predicted to be very similar to this and accommodates this disaccharide well. The *H. sapien* 4XWH and Amuc_0060^{GH89}model do not look like they can accommodate the angle of this substrate very well.

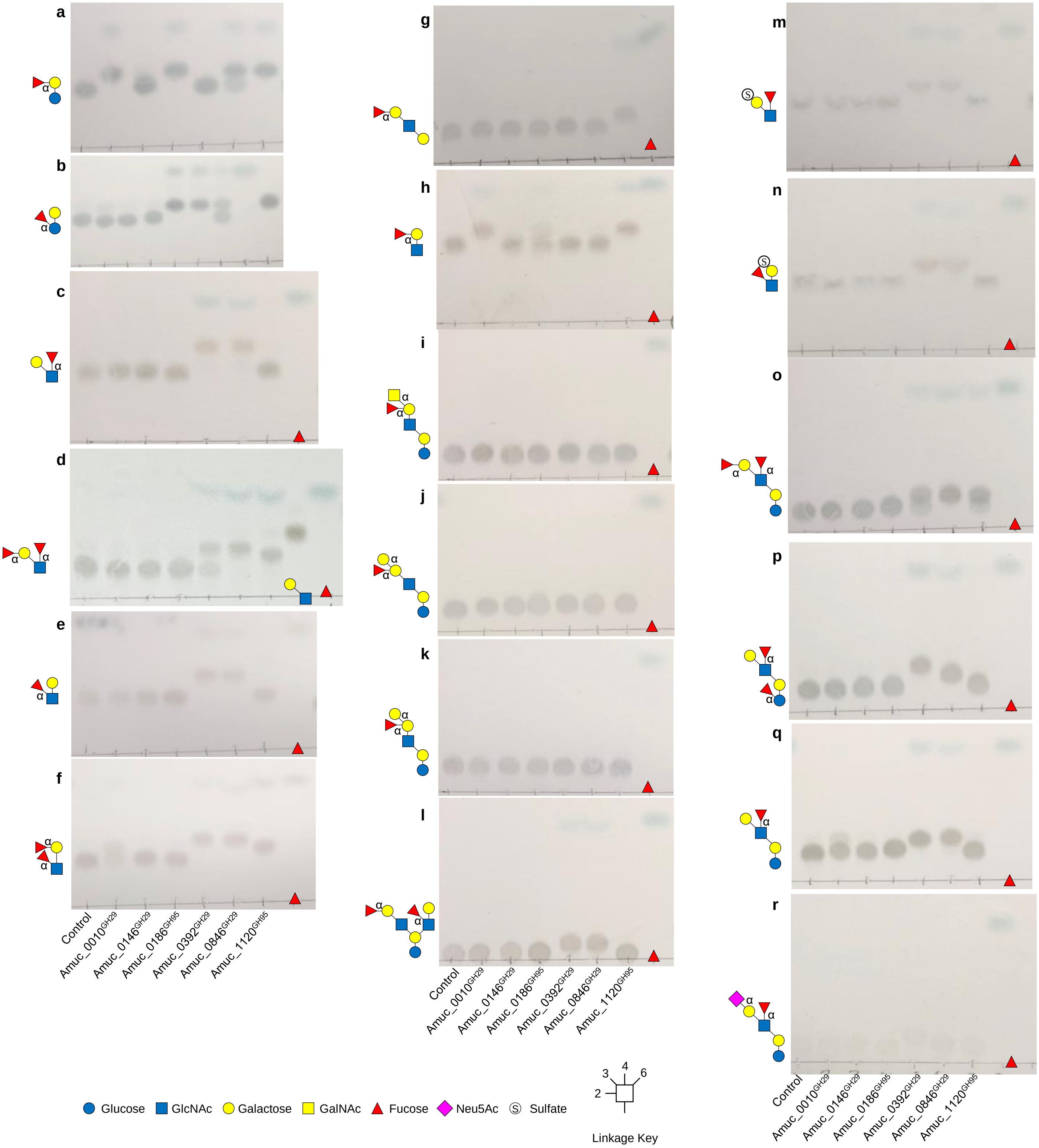

Supplementary Figure 16│Activity of the GH29 and GH95 family members from *A. muciniphila* BAA-835 against defined oligosaccharides, Part 1. a, 2’-fucosyllactose. **b**, 3-fucosyllactose. **c**, Lewis A. **d**, Lewis B. **e**, Lewis X. **f**, Lewis Y. **g**, Blood group H I. **h**, Blood group H II. **i**, Blood group A II. **j**, Blood group B I. **k**, Blood group B II. **l**, difucosyllacto-N-hexaose. **m**, 3’-sulphated Lewis A. **n**, 3’-sulphated Lewis X. **o**, Lacto-N-difucohexaose I. **p**, Lacto-N-difucohexaose I. **q**, Lacto-N-fucopentaose II. **r**, Sialylfucosyllacto-N-tetraose. Standards have also been included on the TLCs. Enzyme assays were carried out at a final substrate concentration of 1 mM, pH 7, 37 °C, overnight, and with 1 μM enzyme.

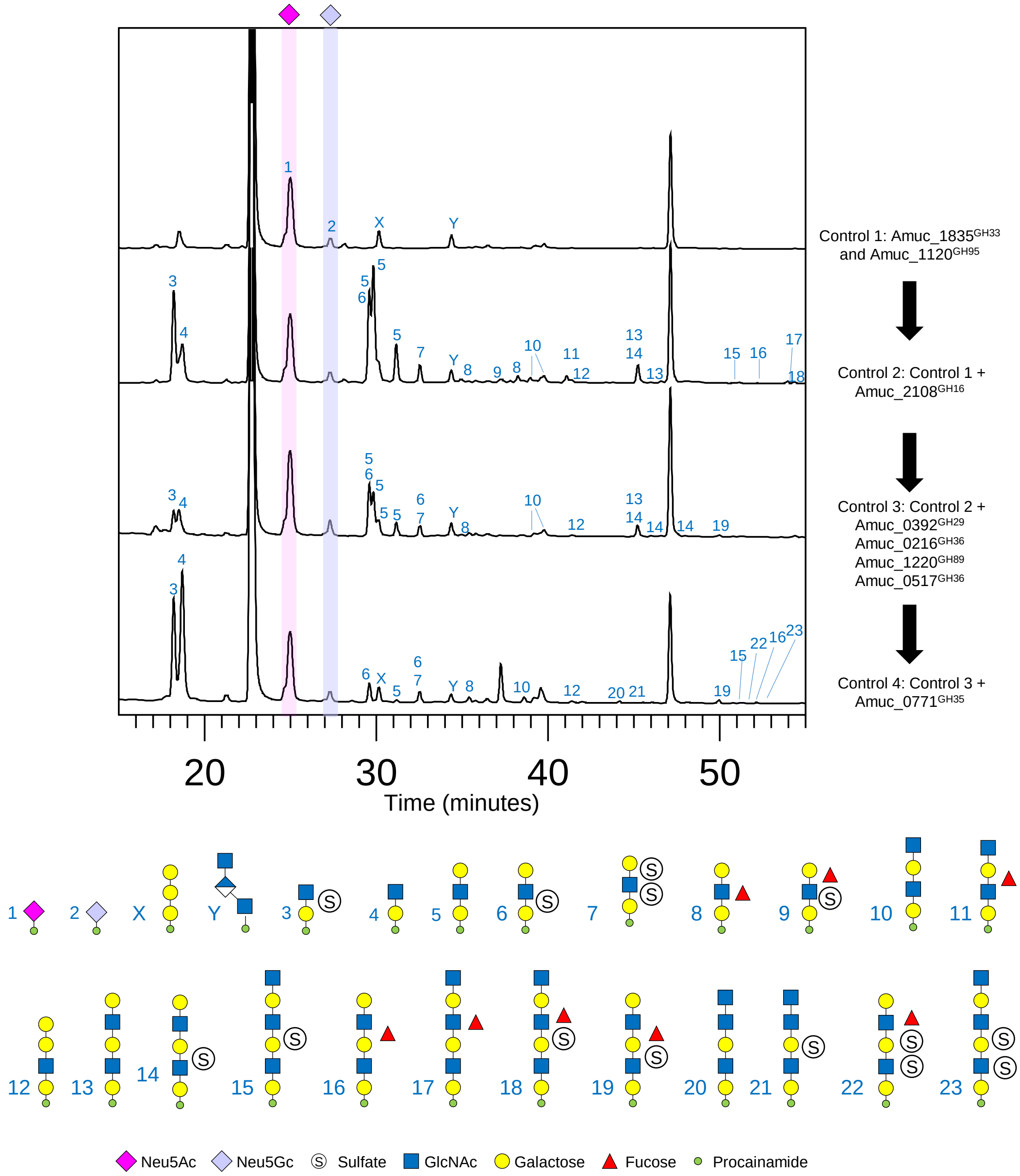

Supplementary Figure 17│Sequential degradation of porcine gastric mucin by CAZymes from AM. Mucin was incubated with different enzymes sequentially and the reaction was stopped in between each step. The glycan products were labelled with procainamide at the reducing end and analysed by LC-FLD-ESI-MS. The results show a release of sialic acid in 1 (other monosaccharides are not resolved under these conditions) and then a release of O-glycan fragments with the addition of a GH16 endo-O-glycanase in 2. 3: The addition of a Amuc_0392^{GH29}, Amuc_1220^{GH89}, Amuc_0517^{GH36} and Amuc_0216^{GH36}. 4: The addition of a broad-acting galactosidase (Amuc_0771^{GH35}).

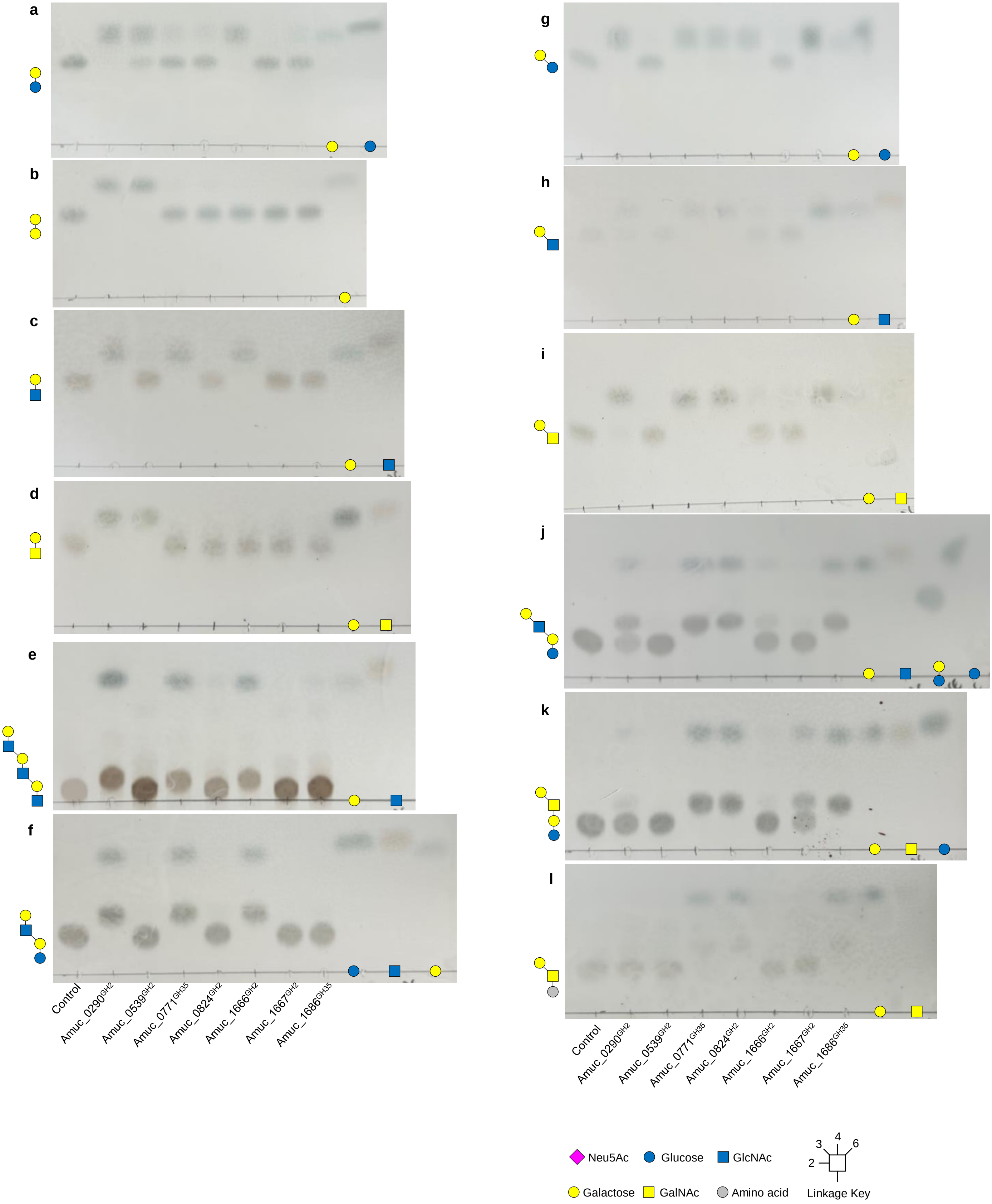

Supplementary Figure 18│Activity of GH2 and GH35 enzymes from *A. muciniphila* BAA-835 against a wide variety of substrates. a, lactose. **b**, Galβ1,4Gal. **c**, LacNAc. **d**, Galβ1,4GalNAc. **e**, TriLacNAc. **f,** Lacto-N-*neo*tetraose. **g,** Galβ1,3Glc. **h,** LNB. **i,** Galβ1,3GalNAc. **j,** Lacto-N-tetrasoe. **k,** GA1. **l,** Tn antigen. Standards have also been included on the TLCs. Enzyme assays were carried out at a final substrate concentration of 1 mM, pH 7, 37 °C, overnight, and with 1 μM enzyme.

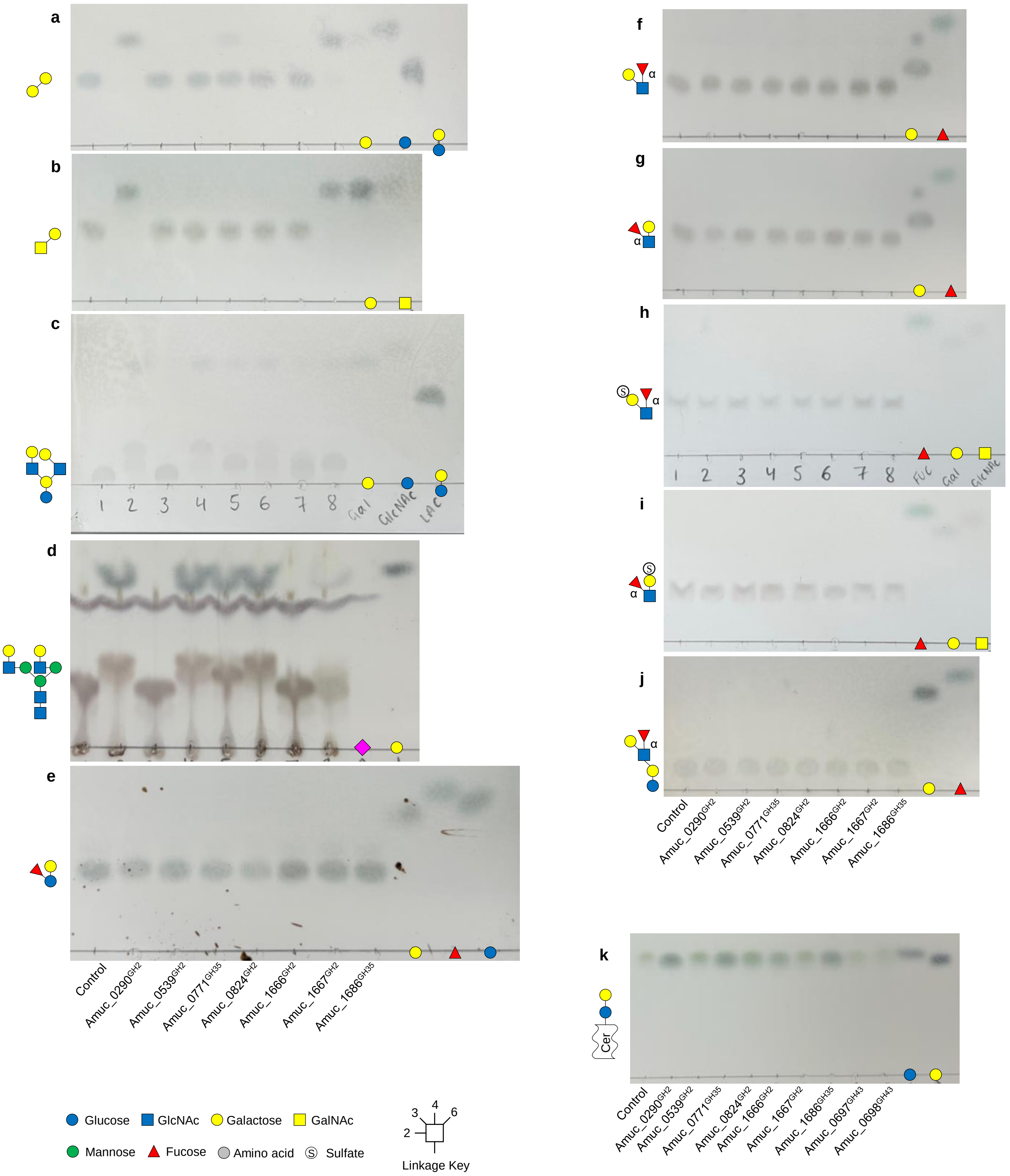

Supplementary Figure 19│Activity of GH2 and GH35 enzymes from *A. muciniphila* BAA-835 against a wide variety of substrates. a, Galβ1,6Gal. **b**, Galβ1,6GalNAc. **c**, Lacto-N-*neo*hexaose. **d**, biantennary complex N-glycans. **e**, 2'FL. **f**, Lewis A. **g,** Lewis X. **h,** 3S Lewis A. **i,** 3S Lewis X. **j,** LNFP II. **k,** Lactosylceramide. Standards have also been included on the TLCs. Enzyme assays were carried out at a final substrate concentration of 1 mM (or 10mg/ml for glycoproteins), pH 7, 37 °C, overnight, and with 1 μM enzyme.

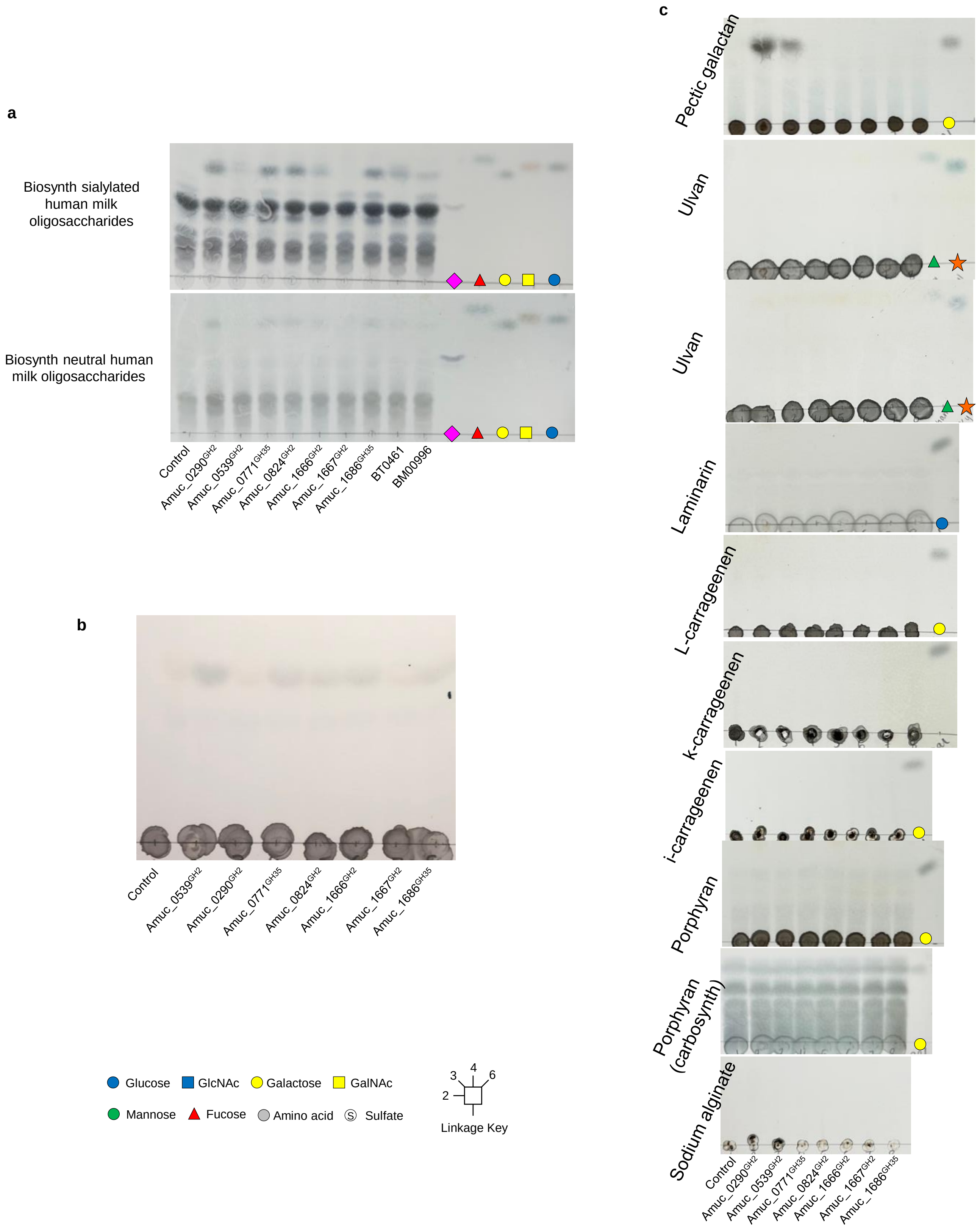

Supplementary Figure 20│Activity of GH2 and GH35 enzymes from *A. muciniphila* BAA-835 against a wide variety of substrates. a, Human milk oligosaccharides. **b**, PGM III. **c**, Range of plant polysaccharides**,** Lactosylceramide. Standards have also been included on the TLCs.

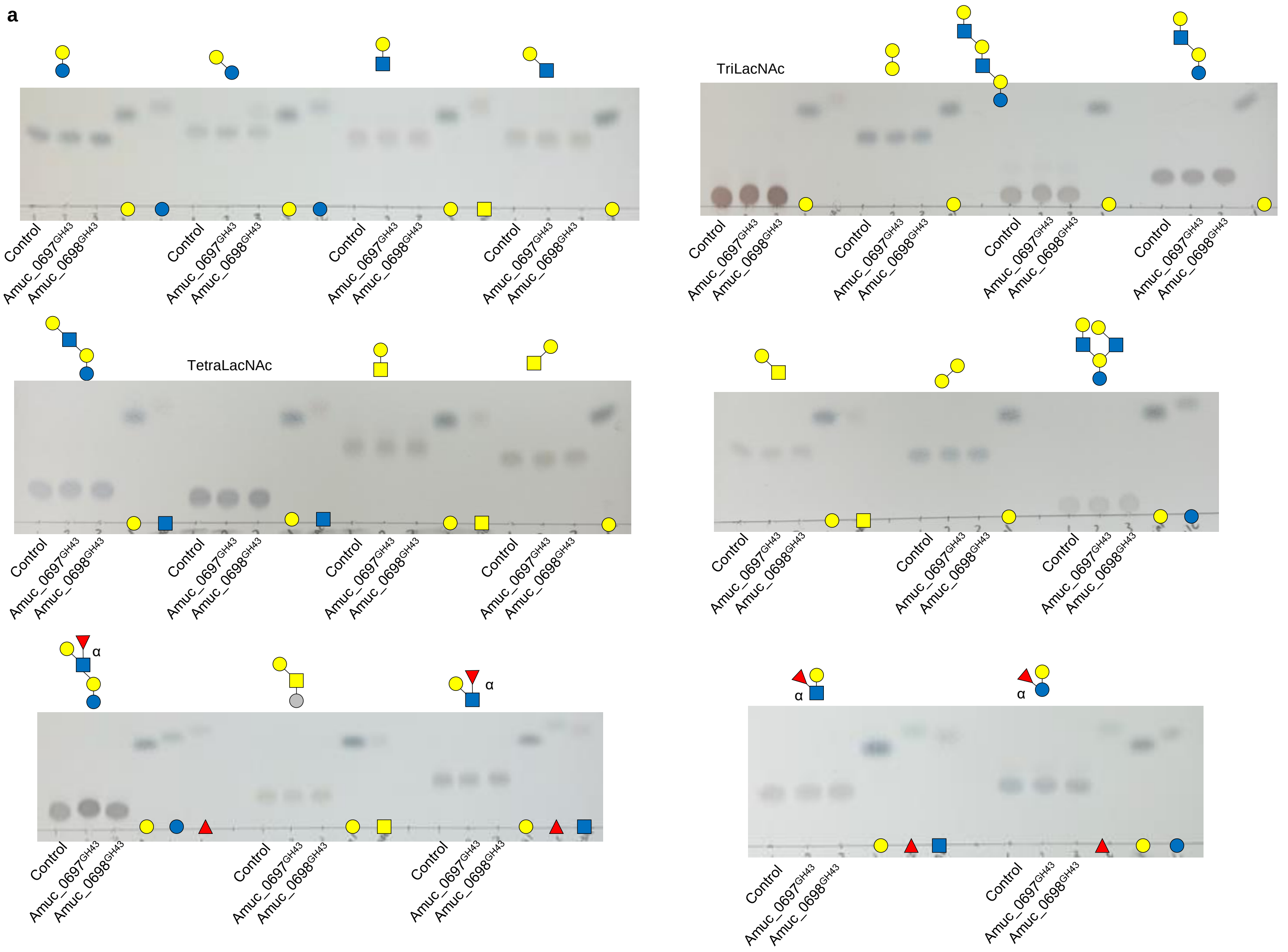

b

```
Amuc_0697      ---MINTMKSLLALCLFYSAASVSTAETPIKHTSFRPGEIWTDNNGAHINAHGGGILYDK 57
AMuc_0698      MKILTAILSANLACCLF----SVAAAETPIKHTSFRPGEIWTDNNGAHINAHGGGILYDK 56
                  :   :.: ** ***    **::***********************************

Amuc_0697      GTYYWYGEHKVEGDAGNYAQVGVHCYSSKDLYNWKDEGIALKVVEGDHSHPIARGCILER 117
AMuc_0698      GTYYWYGEHKVEGDAGNYAQVGVHCYSSKDLYNWKDEGIALKVVEGDHSHPIAKGCILER 116
               *****************************************************:******

Amuc_0697      PKVVYNKKTGKYVMWFHLELKGKGYGSAYAGVATAAKPTGPFKFLKAGRVNPGKWPLNMD 177
AMuc_0698      PKVVYNKKTGKYVMWFHLELKGKGYGSAYAGVATAAKPTGPFKFLKAGRVNPGKWPLNMD 176
               ************************************************************

Amuc_0697      KKDQTMETFKGVPGLTSSALPEGVRSEDMFKKDFEKGQMSRDMTIFVDDDGKAYHIYSSE 237
AMuc_0698      KKDQTTEFTKEMPDYV-----------RIIRRDYPGGQMSRDMTIFVDDNGKAYHIYSSE 225
               ***** *  * :*. .            ::::*:  *************:**********

Amuc_0697      ENSTTHIAELTPDYTGHTGKFVRAFPGRFMEAPAIFKHKGKYYFIASGCTGWAPNAARSA 297
AMuc_0698      GNITLHIAELTPDYTGHTGKYVRAFINRYMEAPAICKHKGKYYLIASGCTGWAPNAARSA 285
                * * ***************:**** .*:****** *******:****************

Amuc_0697      VAKNIAGPWMELKNPCVGPKAGITFGGQSTFILPVQGKPGKFIFMADIWRPQNAIDGRYM 357
AMuc_0698      VAKNIAGPWMELKNPCVGPKAGITFGGQSTFILPVQGKPGKFIFMADIWRPKNAIDGRYL 345
               ***************************************************:*******:

Amuc_0697      WLPIQFDGDQIKLEWKDEWTLKDM         381
AMuc_0698      WLPMKWEGDQIILEWKDEWTLDDL         369
               ***::::**** *********.*:
```

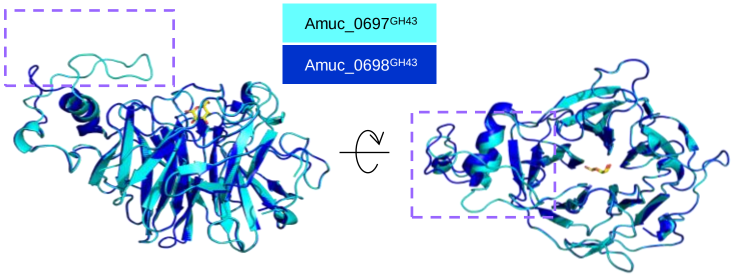

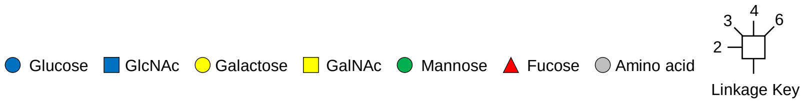

Supplementary Figure 21│The GH43 enzymes of *A. muciniphila* BAA-835. a, Testing activity against a variety of defined substrates. Standards have also been included on the TLCs on the right. Enzyme assays were carried out at a final substrate concentration of 1 mM, pH 7, 37 °C, overnight, and with 1 μM enzyme. **b,** Sequence alignment (left) showing that the sequences are conserved apart from a section in the middle (purple dashed box). Right: Structural models of the two GH43 enzymes superimposed with a galactose in the active site (from 6EUI, which is BT3683 from *Bacteroides thetaiotaomicron*). The only difference between the models is one of the loops on the side of the active site and corresponds to the sequence difference seen with the alignment purple dashed box).

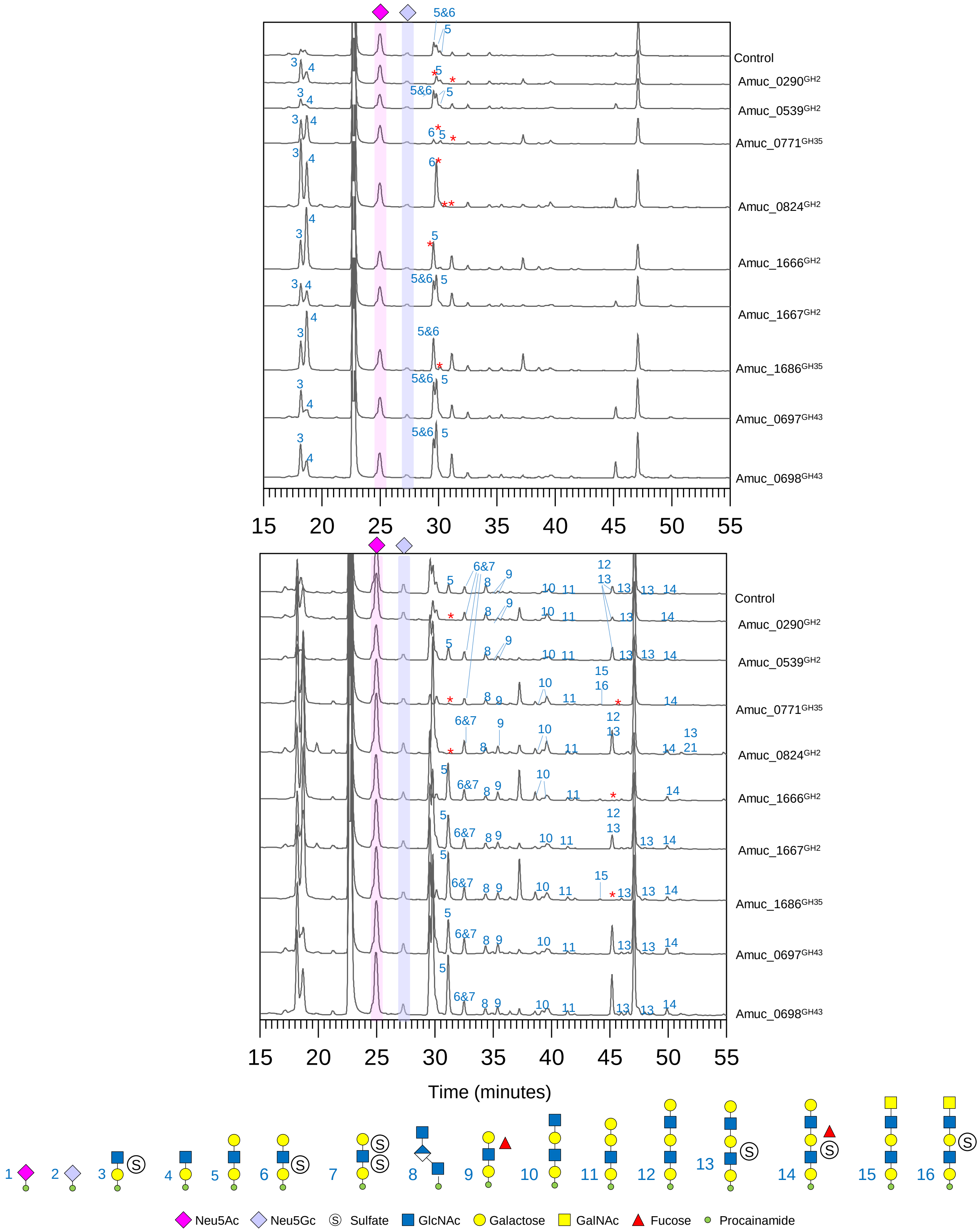

Supplementary Figure 22│Activity of β-galactosidases against O-glycans from PGMIII analysed by LC-FLD-ESI-MS. PGMIII was pretreated sequentially with 1) sialidase and fucosidase, 2) GH16 endo-O-glycanase, and then 3) another fucosidase and three of the CAZymes that remove α-linked capping monosaccharides. The reaction was stopped in between each step and the putative β-galactosidases were added individually. The resulting O-glycans were labelled with procainamide at the reducing end and analysed by LC-FLD-ESI-MS. The chromatograms are presented in two different ways to emphasise the large peaks (top panel) and smaller peaks (bottom panel). The red asterisks indicate where a glycan is no longer present relative to the control and the β-galactosidase has broken this down.

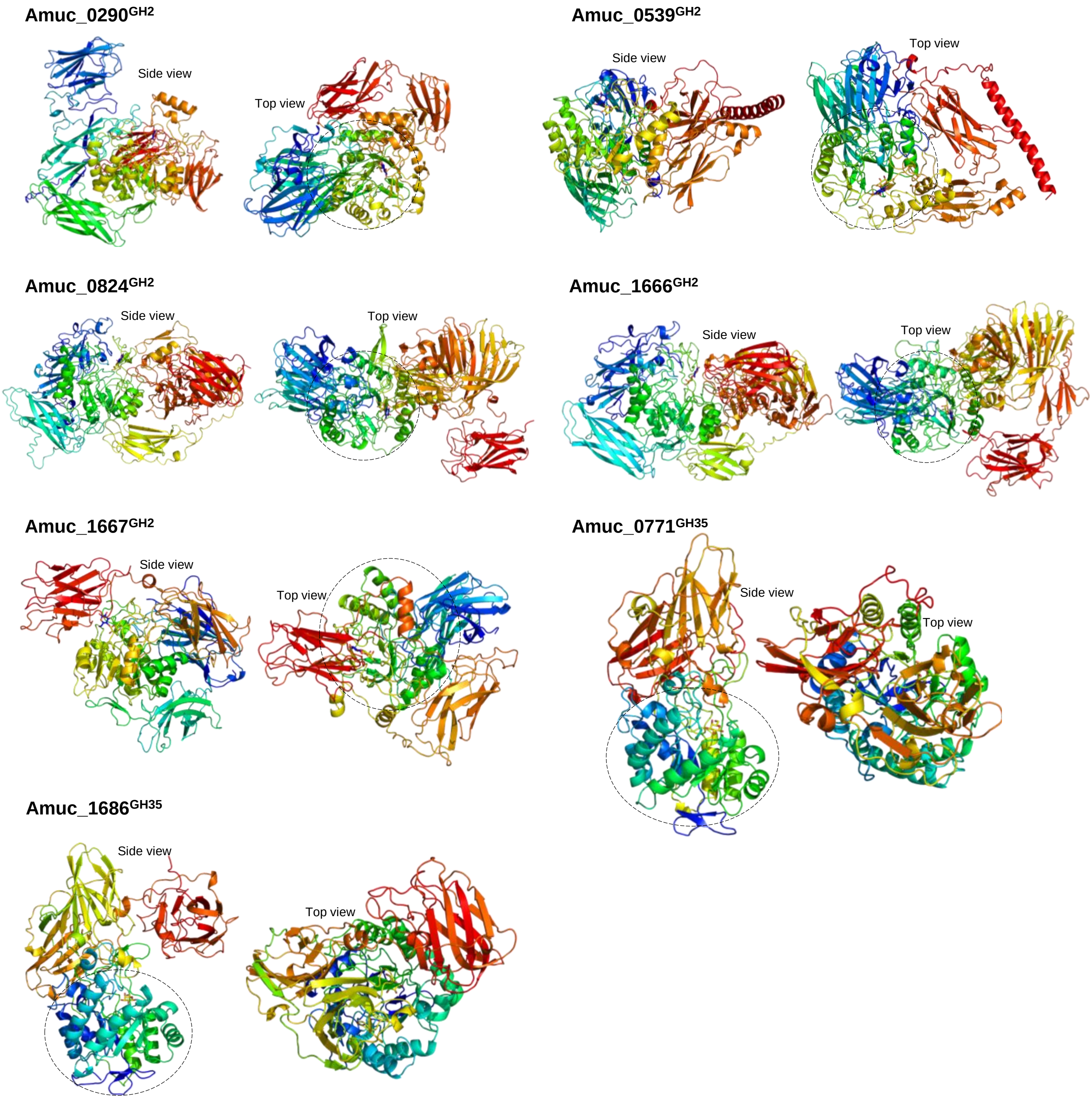

Supplementary Figure 23│Models of the GH2 and GH35 enzymes from *A. muciniphila* BAA-835. Models were built using AlphaFold2 Colab as described in the materials and methods. Lactose from 1JYN was overlaid into the active site of the GH2 enzymes and galactose from 4E8C was overlaid into the active sites of the GH35 enzymes and are shown as sticks. The enzymes are coloured blue to red from the N- to the C-termini, respectively. The catalytic modules are circled with a dotted line to highlight the variety of different accessory modules.

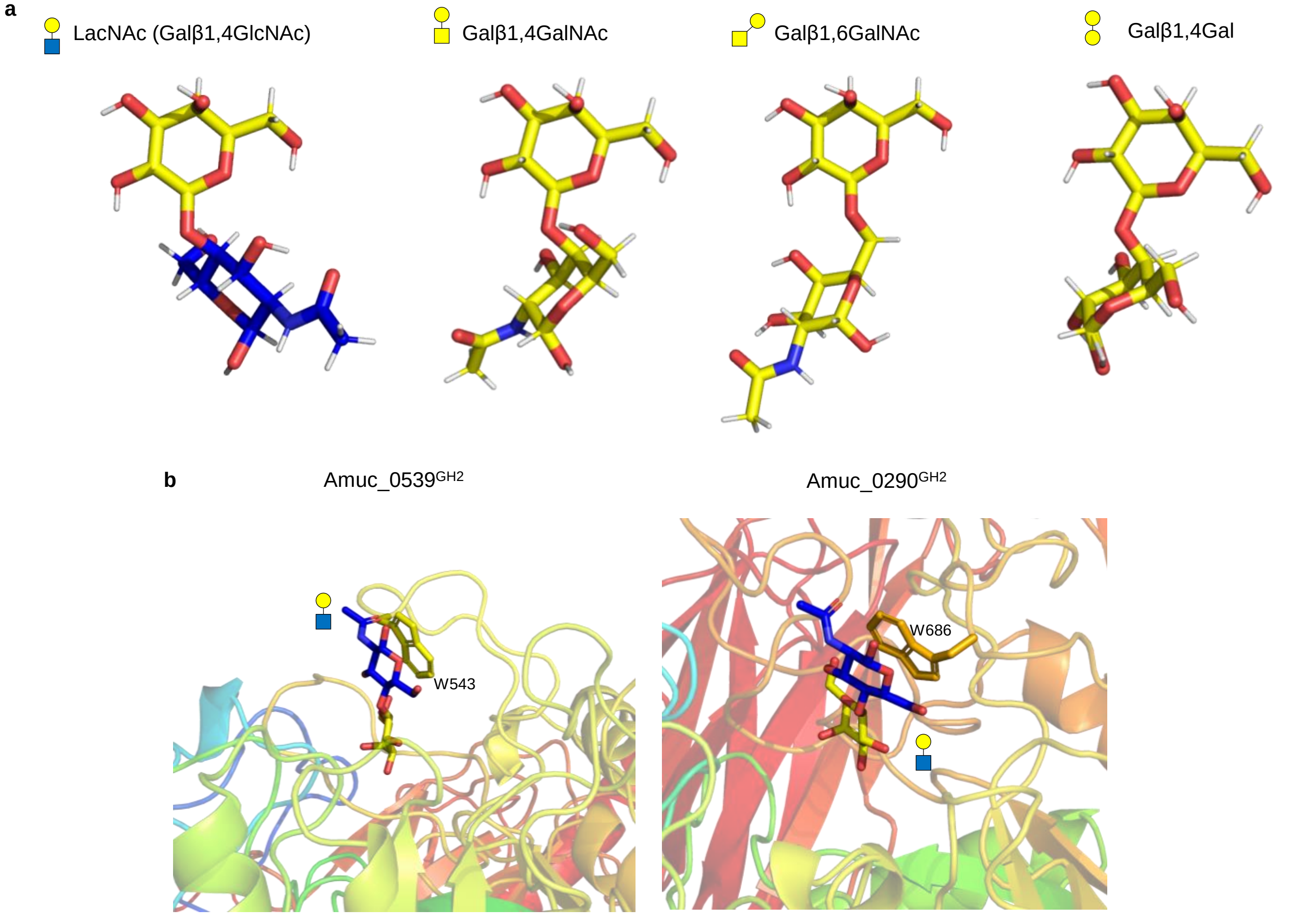

Supplementary Figure 24│Analysis of models of the GH2 enzymes from *A. muciniphila* BAA-835. a, Models of different disaccharides. **b,** Models of two GH2 enzymes with LacNAc overlaid into the active site (from a crystal structure of a *Streptococcus pneumoniae* GH2, 4CUC). Aromatic residues contact the +1 sugar to likely play a role in selectivity and substrate binding.

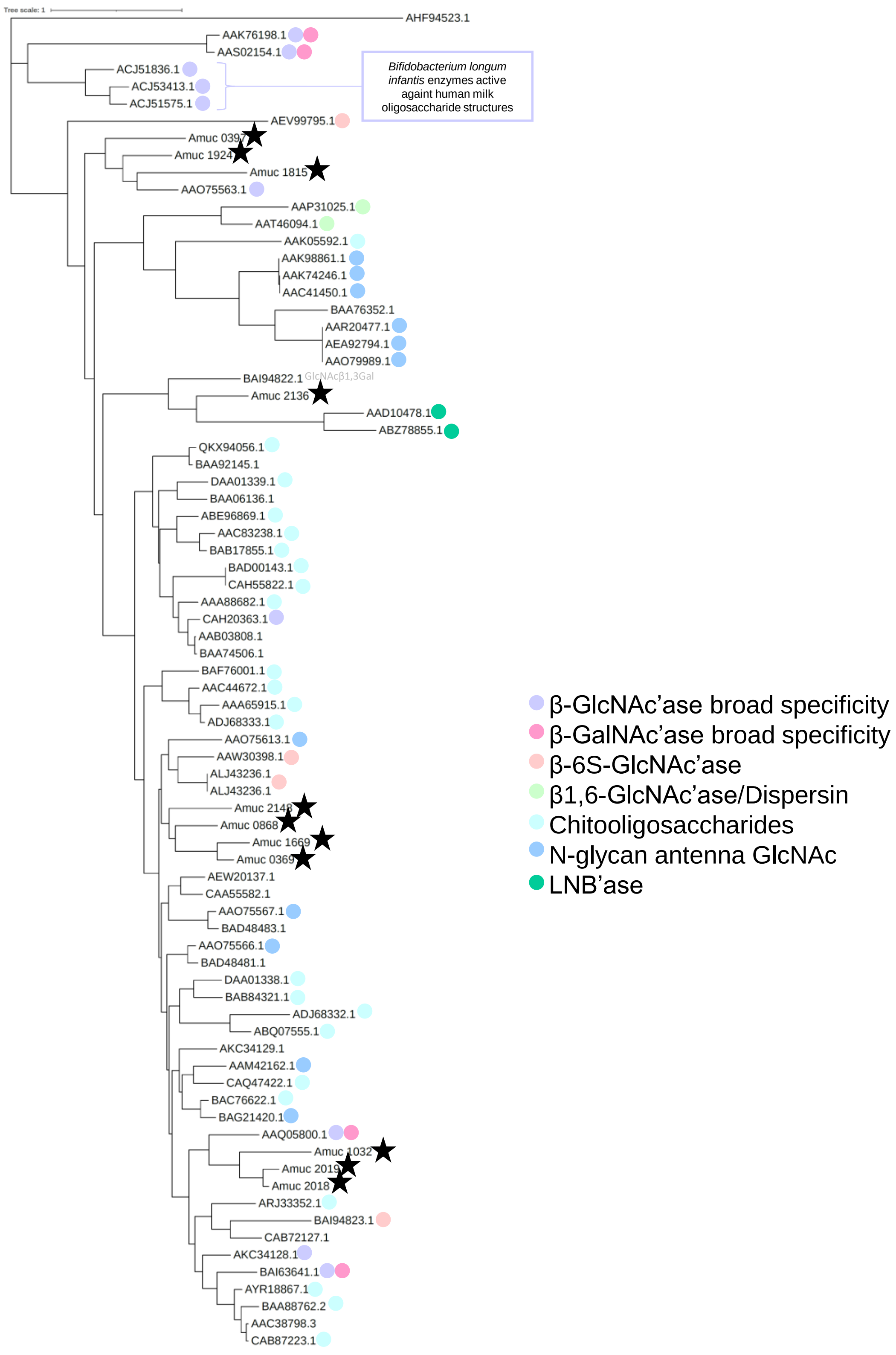

Supplementary Figure 25│Phylogenetic tree of characterised GH20 family members with those from *A. muciniphila* ATCC BAA-835. The sequences of the GH20 family members with reported activities (CAZy database) and the ones from *A. muciniphila* ATCC BAA-835 were compared as described in the methods. The different specificities are indicated by different colours and the *A. muciniphila* ATCC BAA-835 enzymes are highlighted by black stars. Some more specific information about activity is supplied were possible. The enzymes are represented by their accession numbers of locus tags. Different specificities cluster in this analysis and the observed activity of the three *A. muciniphila* ATCC BAA-835 enzymes correlates to where they cluster on the tree.

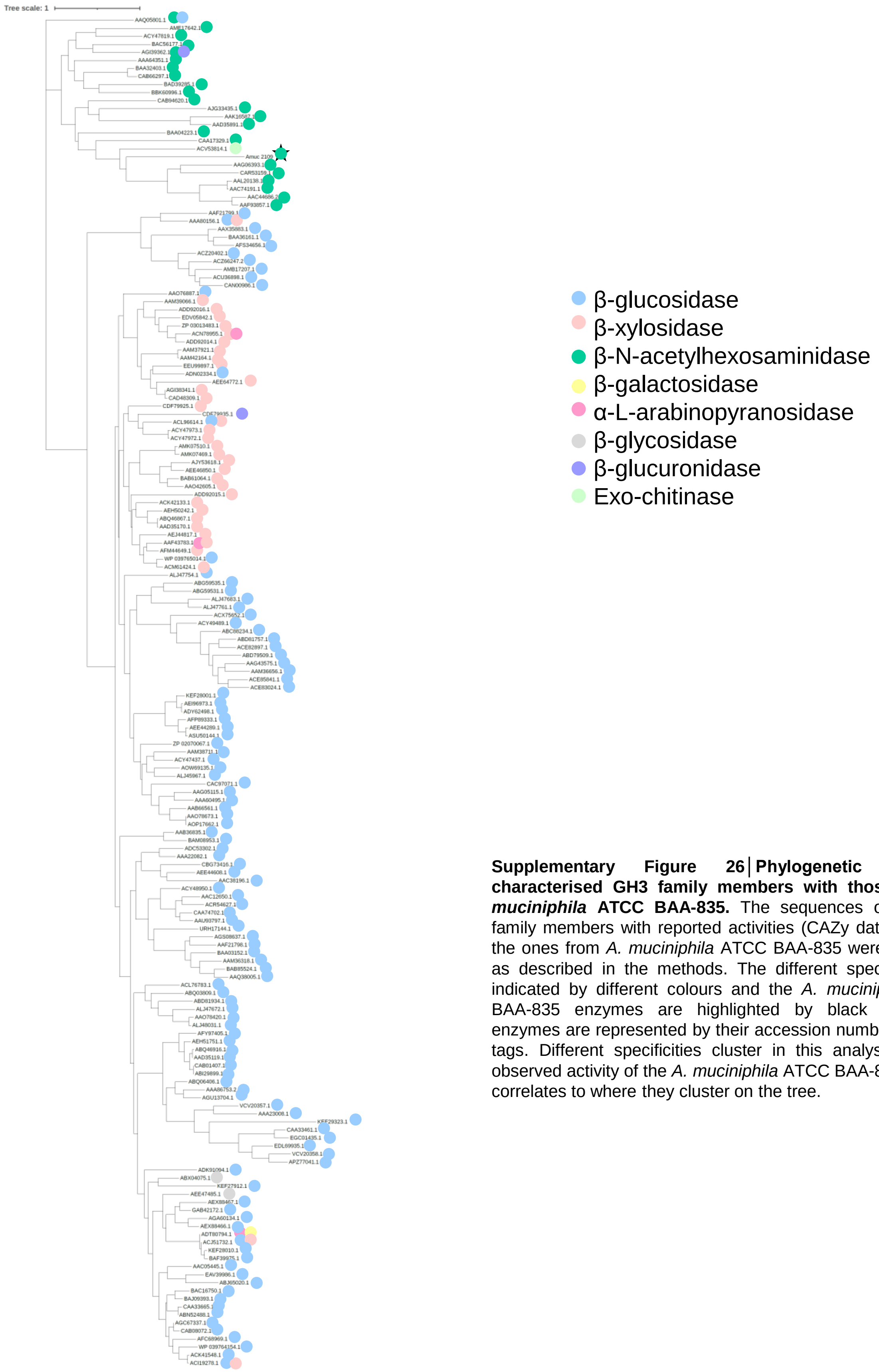

Supplementary Figure 26 | Phylogenetic tree of characterised GH3 family members with those from *A. muciniphila* ATCC BAA-835. The sequences of the GH3 family members with reported activities (CAZy database) and the ones from *A. muciniphila* ATCC BAA-835 were compared as described in the methods. The different specificities are indicated by different colours and the *A. muciniphila* ATCC BAA-835 enzymes are highlighted by black stars. The enzymes are represented by their accession numbers of locus tags. Different specificities cluster in this analysis and the observed activity of the *A. muciniphila* ATCC BAA-835 enzyme correlates to where they cluster on the tree.

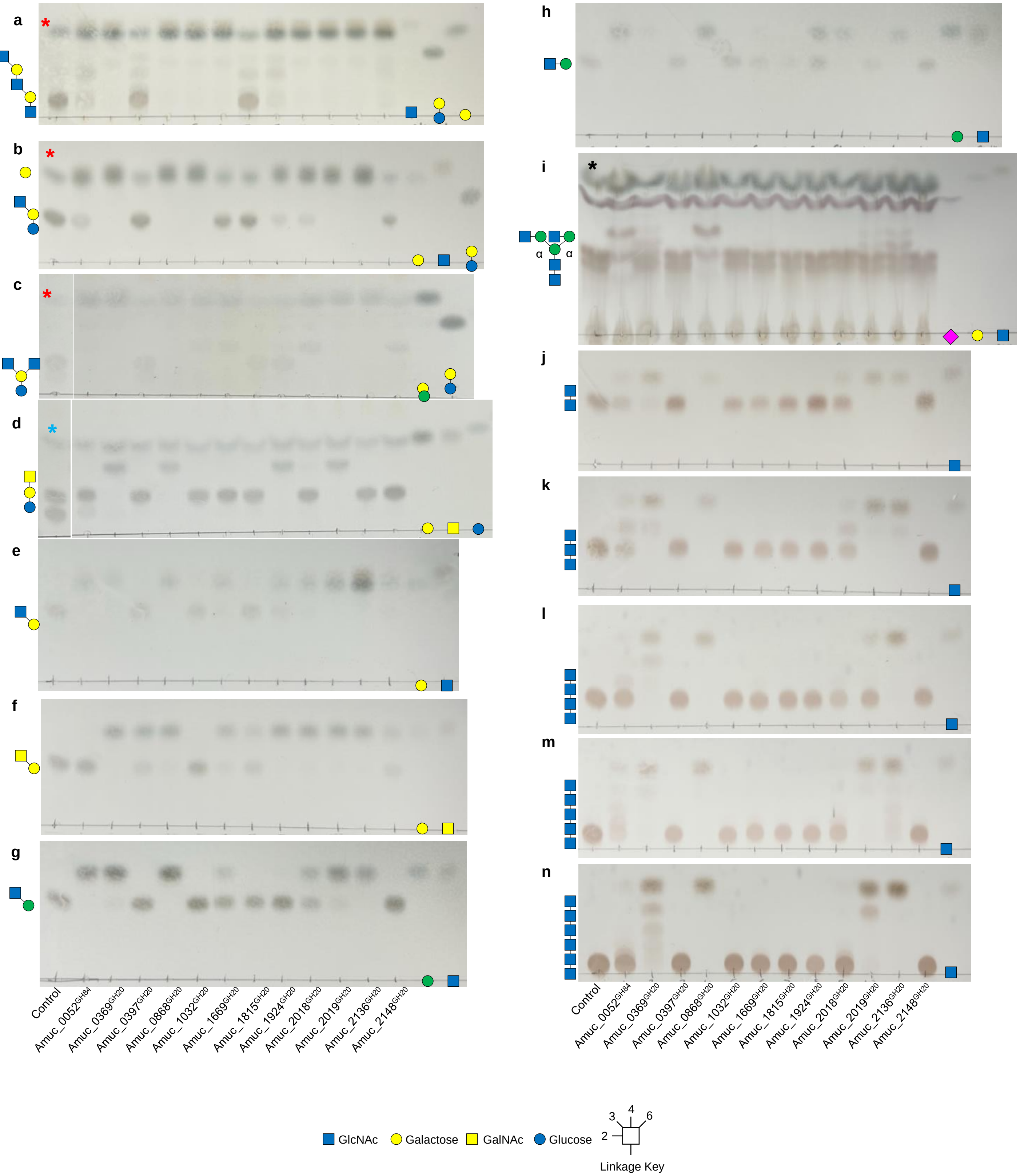

Supplementary Figure 27│Activity of the GH20 and GH84 family members from *A. muciniphila* BAA-835 against defined oligosaccharides. Enzyme assays were carried out at a final substrate concentration of 1 mM, pH 7, 37 °C, overnight, and with 1 μM enzymes. **a,** TriLacNAc. **b**, Lacto-N-neotetraose. **c**, Lacto-N-neohexaose. **d**, Galβ1,3GalNAcβ1,4Galβ1,4Glc. **e**, GlcNAcβ1,3Gal. **f**, GalNAcβ1,3Gal. **g**, GlcNAcβ1,3Man. **h**, GlcNAcβ1,2Man. **i**, $\alpha_1$acidglycoprotein. **j**, Chitobiose. **k**, Chitotriose. **l**, Chitotetraose. **m**, Chitopentaose. **n**, Chitohexaose. Some pre-treatments were required to generate the substrates: Red asterisk - pre-treated with BT0461^{GH2} to remove the non-reducing end β1,4galactose, cyan asterisk - pre-treated with B035DRAFT_00996^{GH2} to remove the non-reducing end β1,3galactose, black asterisk – pre-treated with BT0455^{GH33}, BT0461^{GH2}, and PNGaseL, which are a broad-acting sialidase, β1,4-galactosidase, and remove N-glycans from the protein, respectively. The glycan that the GH20 enzymes are tested on are shown to the right of each TLC and all linkages are beta. Standards have also been included on the left of the TLCs.

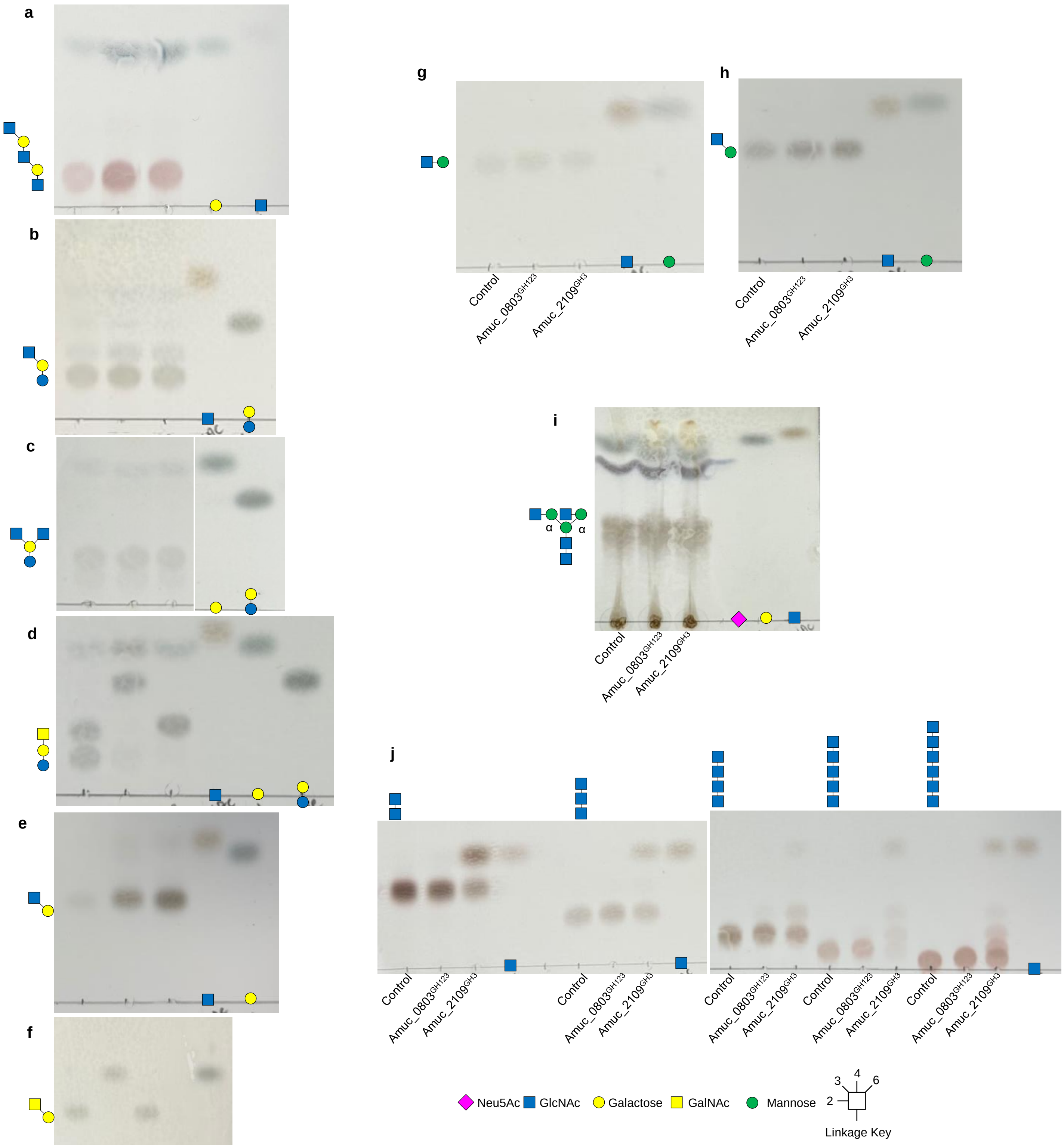

Supplementary Figure 28│Activity of the GH123 and GH3 family members from *A. muciniphila* BAA-835 against defined oligosaccharides. Enzyme assays were carried out at a final substrate concentration of 1 mM, pH 7, 37 °C, overnight, and with 1 μM enzymes. **a,** TriLacNAc. **b**, Lacto-N-neotetraose. **c**, Lacto-N-neohexaose. **d**, Galβ1,3GalNAcβ1,4Galβ1,4Glc. **e**, GlcNAcβ1,3Gal. **f**, GalNAcβ1,3Gal. **g**, GlcNAcβ1,3Man. **h**, GlcNAcβ1,2Man. **i**, $\alpha_1$acidglycoprotein. **j**, Chitobiose-Chitohexaose, left to right. Some pre-treatments were required to generate the substrates: Red asterisk - pre-treated with BT0461^{GH2} to remove the non-reducing end β1,4galactose, cyan asterisk - pre-treated with B035DRAFT_00996^{GH2} to remove the non-reducing end β1,3galactose, black asterisk – pre-treated with BT0455^{GH33}, BT0461^{GH2}, and PNGaseL, which are a broad-acting sialidase, β1,4-galactosidase, and remove N-glycans from the protein, respectively. The glycan that the GH20 enzymes are tested on are shown to the right of each TLC and all linkages are beta. Standards have also been included on the left of the TLCs.

a

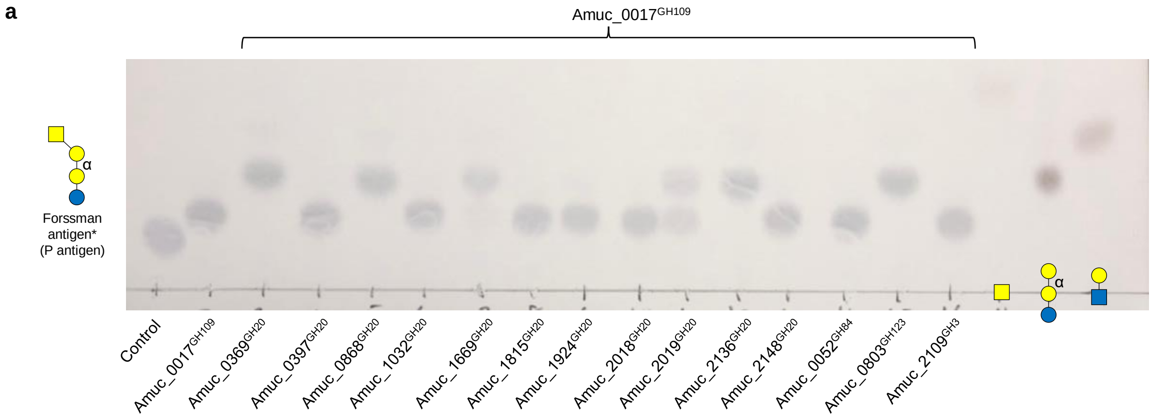

b

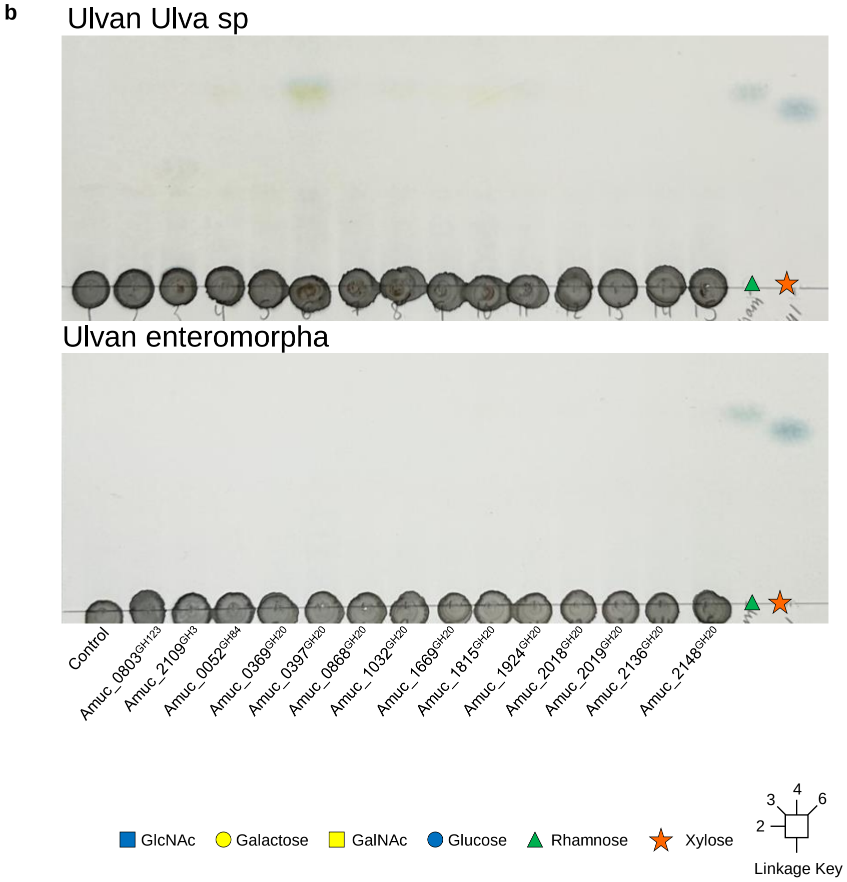

Supplementary Figure 29│Activity of the β-HexNAc'ases from *A. muciniphila* BAA-835 against P antigen and ulvan substrates. Enzyme assays were carried out at pH 7, 37 °C, overnight, and with 1 μM enzymes. Standards have also been included on the left of the TLCs. The Forssman antigen was treated with Amuc_0017^{GH109}

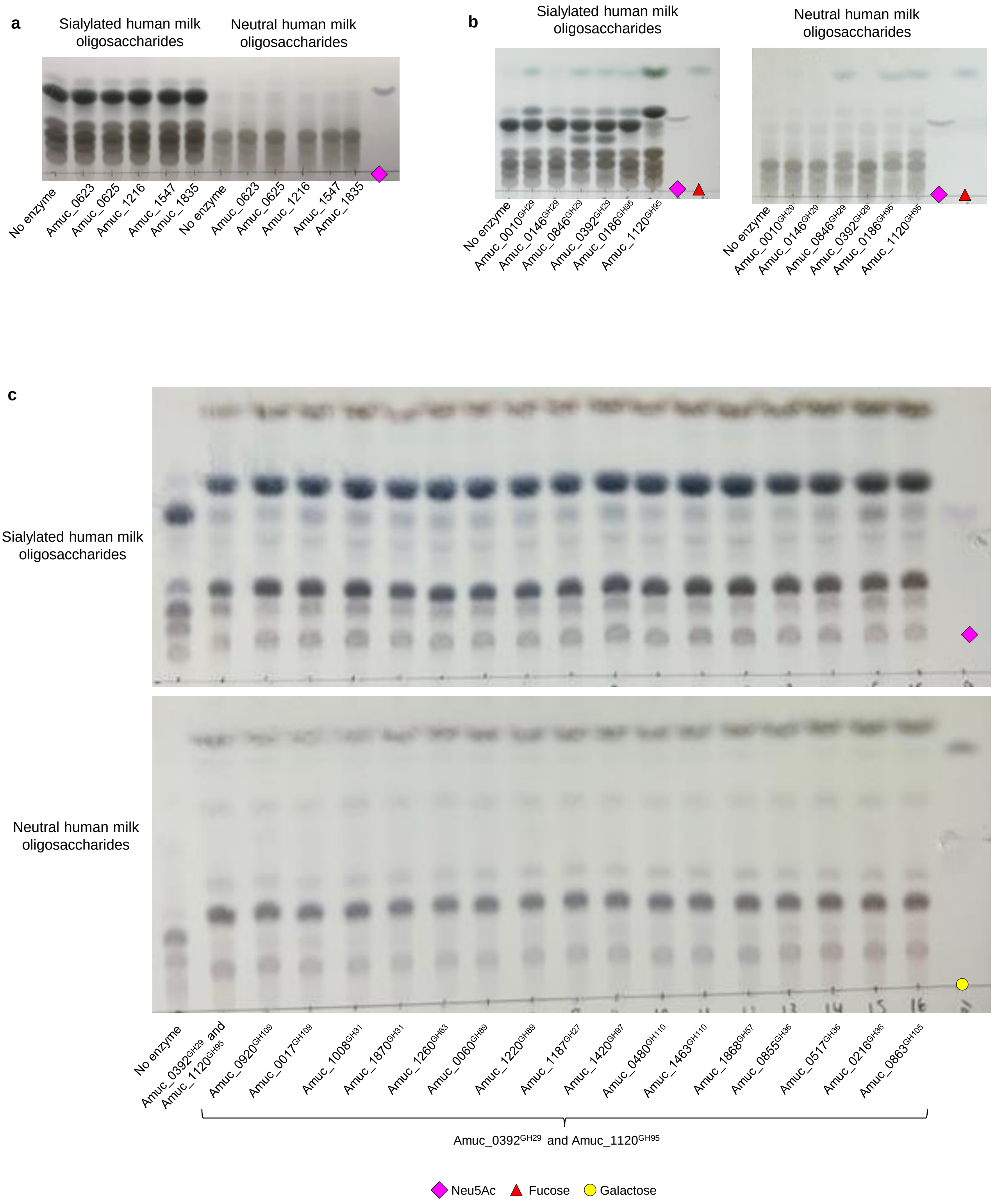

Supplementary Figure 30│Activity of sialidases, fucosidases and other α-linkage acting GH enzymes from AM against human milk oligosaccharides. a, Sialidases and potential sialidases. **b**, Fucosidases. **c**, CAZymes to predicted to act on α-linked monosaccharides and these samples were also pre-treated with two fucosidases. Standards have also been included on the TLCs. Enzyme assays were carried out at a final substrate concentration of 1 mM, pH 7, 37 °C, overnight, and with 1 μM enzyme.

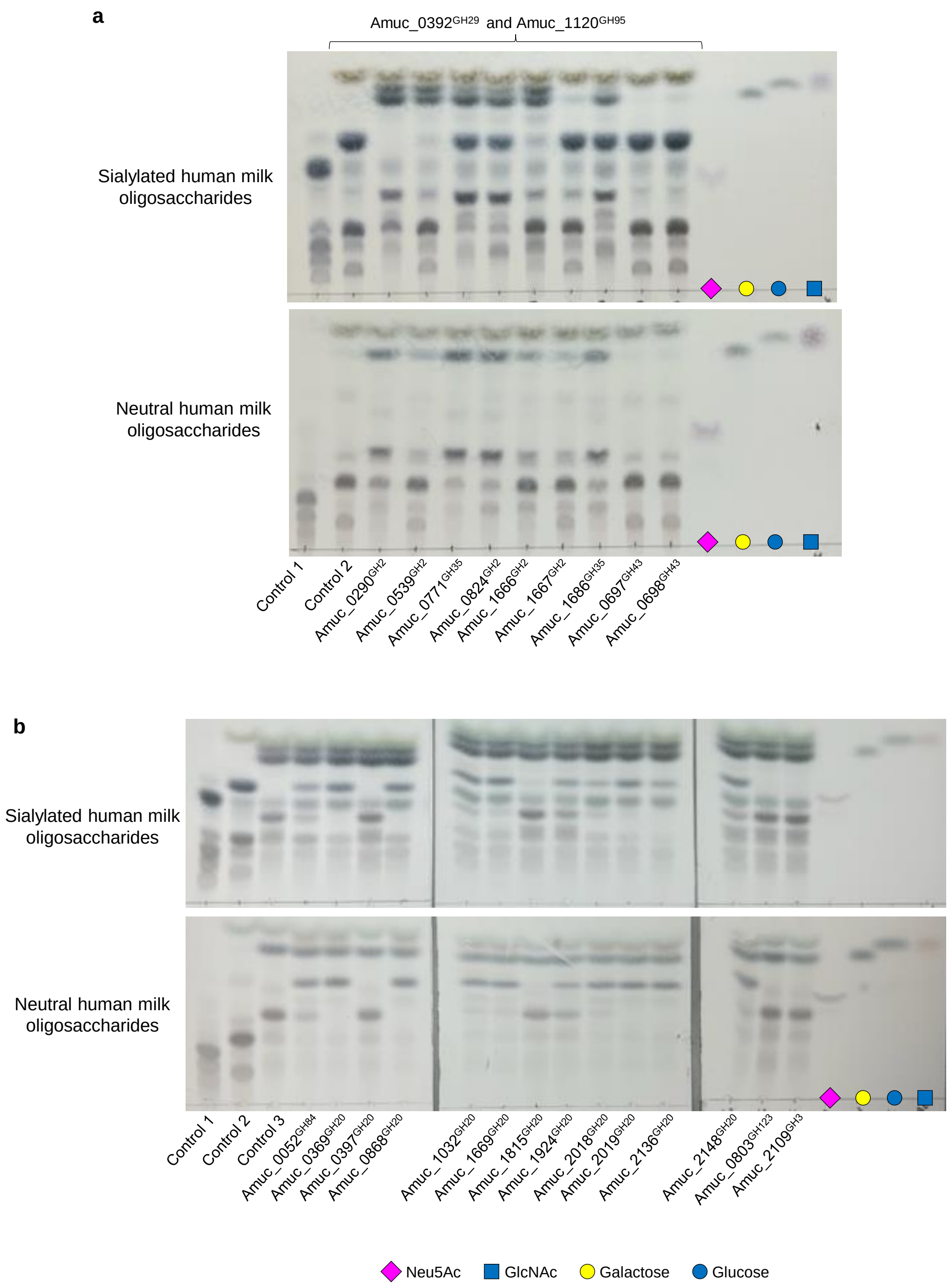

Supplementary Figure 31│Activity of β-galactosidase and β-HexNAc'ase GH enzymes from AM against human milk oligosaccharides. a, β-galactosidases. Top and bottom panels are sialylated and neutral HMO preparations (Biosynth). Control 1 – no enzymes added and Control 2 - Amuc_0392^{GH29} and Amuc_1120^{GH95}. The rest of the lanes are the individual enzymes with the fucosidases are also included in these reactions. **b**, β-HexNAc'ase. Top and bottom panels are sialylated and neutral HMO preparations (Biosynth). Control 1 – no enzymes added and Control 2 - Amuc_0392^{GH29} and Amuc_1120^{GH95}. Control 3 – Top panel: Amuc_0517^{GH36} and Amuc_0290^{GH2}. Bottom panel: Amuc_0216^{GH36} and Amuc_0771^{GH35}. The controls were boiled in between all stages to produce a sequential degradation. The rest of the lanes are the individual β-HexNAc'ases. Standards have also been included on the TLCs. Enzyme assays were carried out at a final substrate concentration of 1 mM, pH 7, 37 °C, overnight, and with 1 μM enzyme.

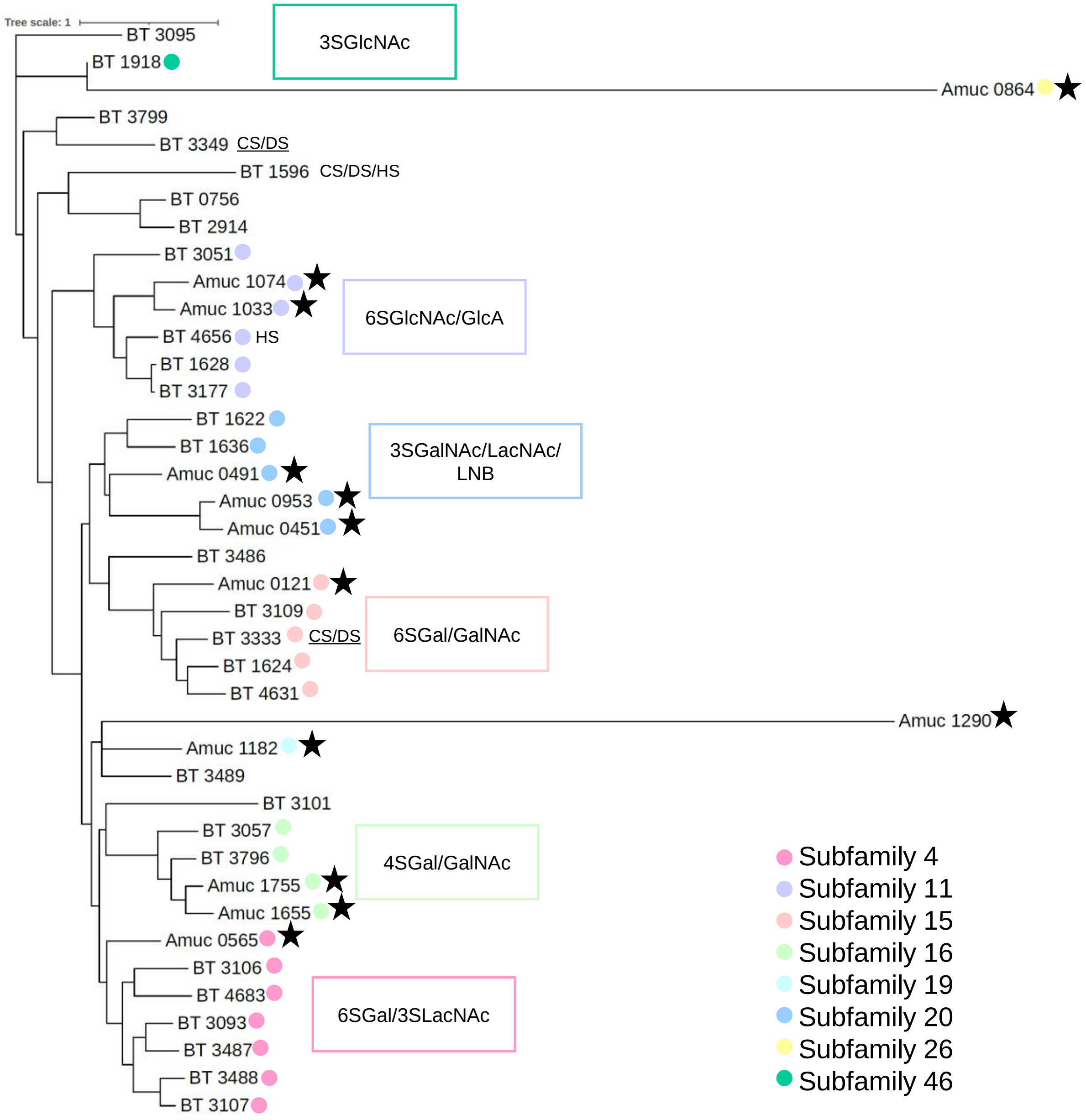

Supplementary Figure 32│Phylogenetic tree of characterised sulfatase family members from *Bacteroides thetaiotaomicron* with those from *A. muciniphila* ATCC BAA-835. The sequences of the sulfatases with reported activities and the ones from *A. muciniphila* ATCC BAA-835 were compared as described in the methods. The different subfamilies are indicated by different colours and the *A. muciniphila* ATCC BAA-835 enzymes are highlighted by black stars. Some more specific information about activity is supplied were possible. When a sulfatase is known to act on a particular GAG, then this is noted and underlined. The enzymes are represented by their locus tags.

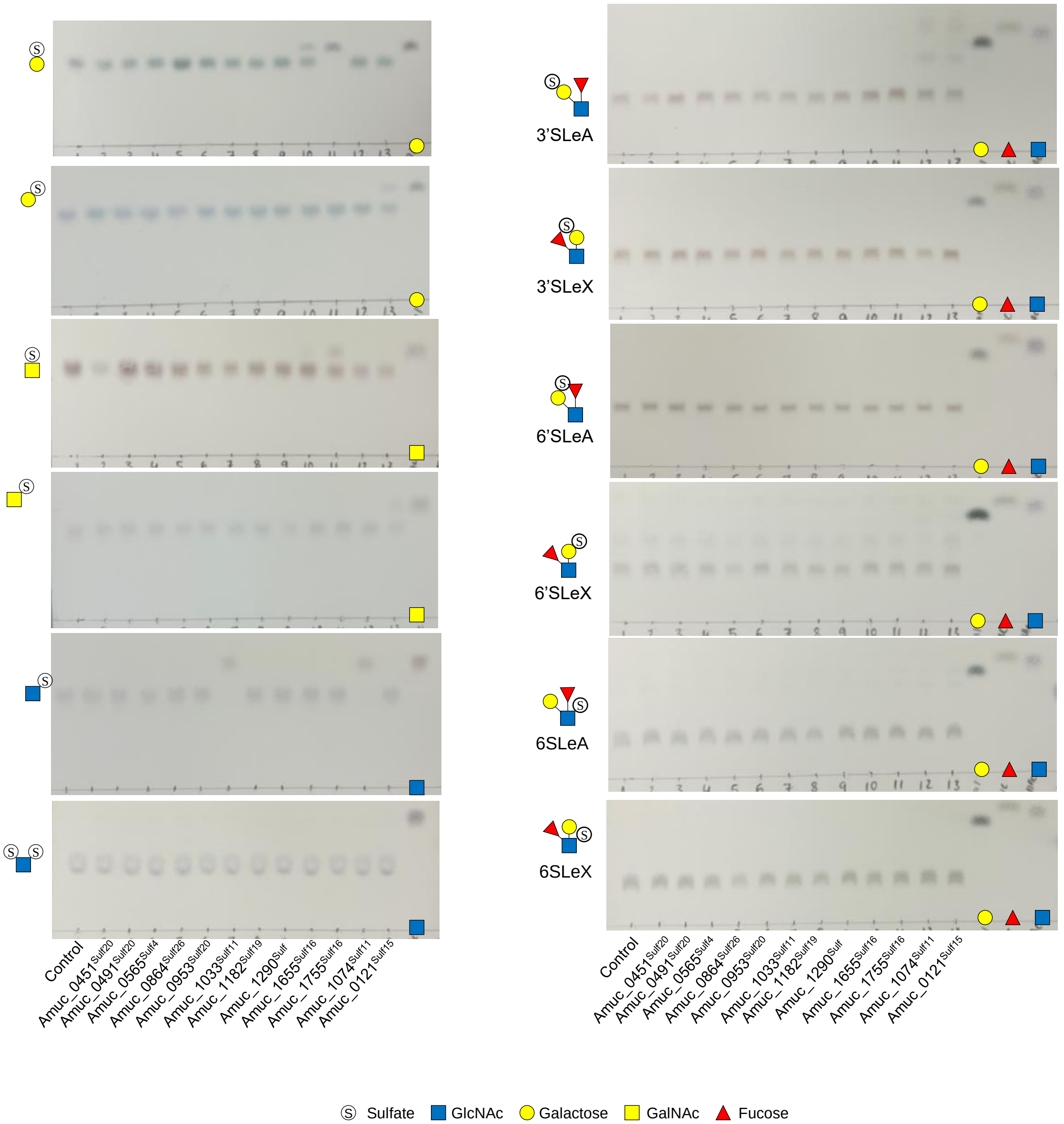

Supplementary Figure 33│Activity of sulfatases from *A. muciniphila* BAA-835 against defined sulfated monosaccharides and Lewis Structures. Standards have also been included on the TLCs. Enzyme assays were carried out at a final substrate concentration of 1 mM, pH 7, 37 °C, overnight, and with 1 µM enzyme.

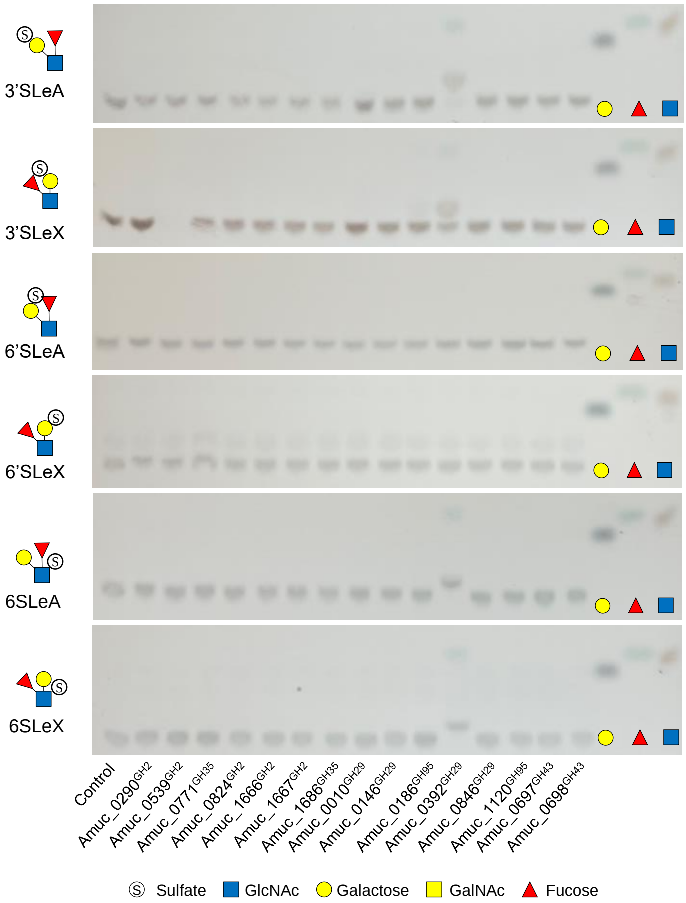

Supplementary Figure 34│Activity of β-galactosidases and α-fucosidases from *A. muciniphila* BAA-835 against sulfated Lewis Structures. Standards have also been included on the TLCs. Enzyme assays were carried out at a final substrate concentration of 1 mM, pH 7, 37 °C, overnight, and with 1 μM enzyme.

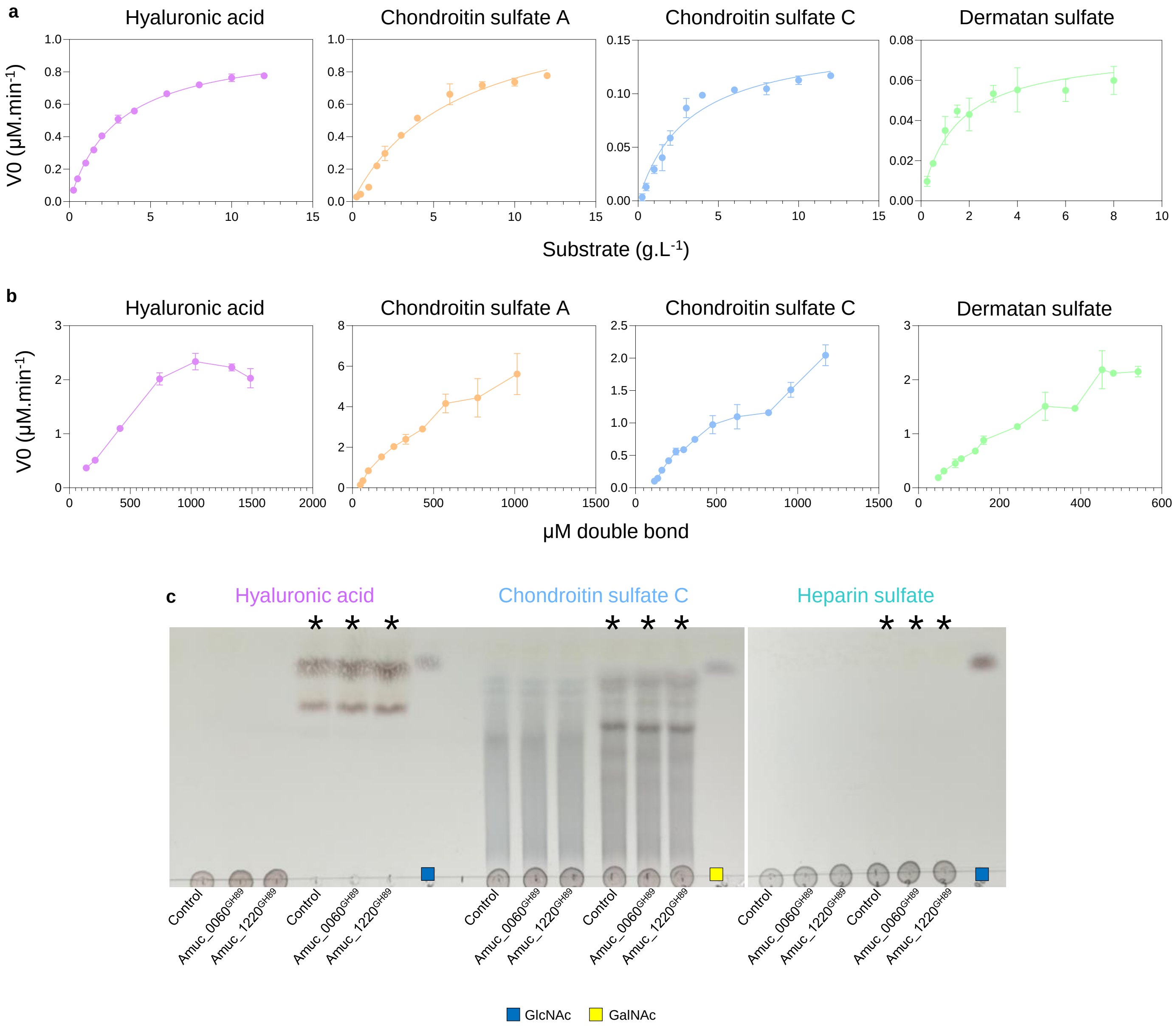

Supplementary Figure 35│Enzyme kinetics for Amuc_0778$^{\text{PL38}}$ and Amuc_0863$^{\text{GH105}}$ against GAG substrates. a, Initial rates of Amuc_0778$^{\text{PL38}}$. with increasing substrate concentrations. The Michaelis-Menten model was fitted in Graph-Prism. Error-bars represents standard deviations from triplicate experiments. **b,** Initial rates of Amuc_0863$^{\text{GH105}}$ for increasing substrate concentrations. The substrate concentrations were obtained directly from the AmPL38 kinetic experiments, and the initial rate defined as loss/converted double-bond per minute. In cases were the initial absorbance of substrate exceeded 2.7 (A235) the data were omitted. No kinetic models could be fitted reliably to any of the datasets. Error-bars represent standard deviations from triplicate experiments. **c,** Testing if the GH89 enzymes have activity against GAGs. Asterisks indicate the presence of Amuc_0778$^{\text{PL38}}$ and Amuc_0863$^{\text{GH105}}$. Standards have also been included on the TLCs. Enzyme assays were carried out at pH 7, 37 °C, overnight, and with 1 µM enzyme. Data are presented as mean values +/- standard deviation and experiments were completed in triplicate.

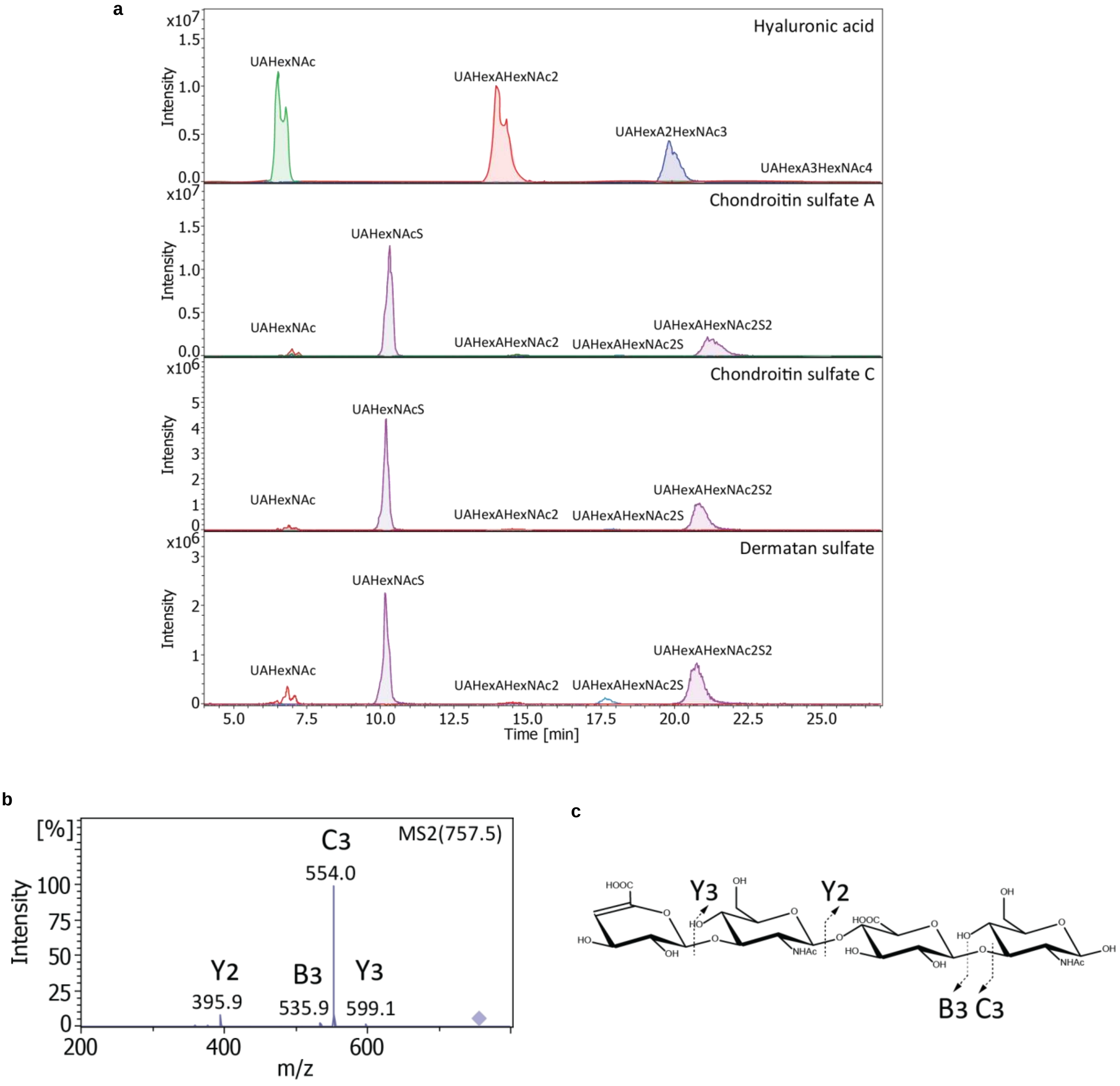

Supplementary Figure 36│Examples of the LC-MS data of Amuc_0778^{PL38} against GAG substrates. a, Extracted ion chromatograms corresponding to the most abundant compounds during Amuc_0778^{PL38} degradation of hyaluronic acid, chondroitin sulfate A, chondroitin sulfate C, and dermatan sulfate. Extracted masses: UAHexNAc2: [M-H]- 378 Da, [2M-H]- 757 Da; UAHexAHexNac2: [M-H]2- 378 Da, [M-H]- 757 Da; UAHexA2HexNac3: [M-H]2- 567.5 Da, [M-H]- 1136.1 Da, UAHexA3HexNac4: [M-H]2- 757 Da, UAHexNAc2S: [M-H]- 458 Da; UAHexAHexNac2S: [M-H]2- 418 Da, [M-H]- 837 Da; UAHexAHexNac2S2: [M-H]2- 458 Da. **b,** Negative mode ESI MS^2 fragmentation of UAHexAHexNAc2, a product of hyaluronic acid degradation by Amuc_0778^{PL38}. Diamond indicates precursor ion at 757.5 Da. Fragments are following the nomenclature of Domon and Costello are indicated on both MS spectrum and structure. **c,** A structure of the glycan corresponding to the fragmentation pattern in 'b'.

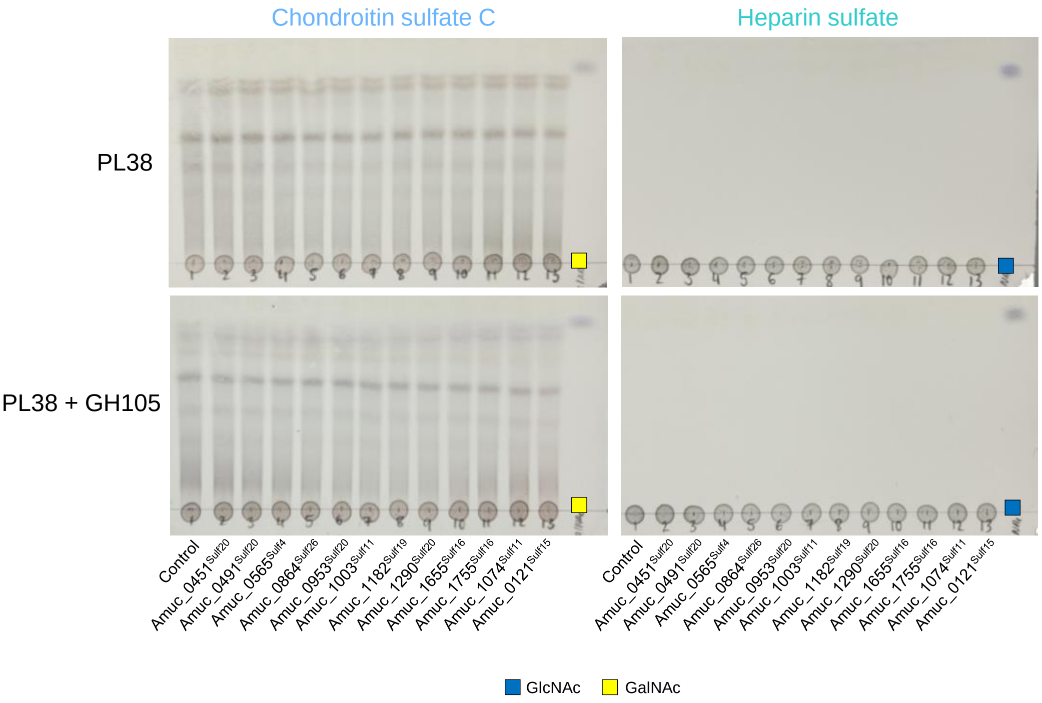

Supplementary Figure 37│Activity of sulfatases from *A. muciniphila* BAA-835 against GAGs. The putative sulfatases were tested against two types of sulfated GAGs in the presence of Amuc_0778^{PL38} and Amuc_0863^{GH105}. Standards have also been included on the TLCs. Enzyme assays were carried out at pH 7, 37 °C, overnight, and with 1 μM enzyme.

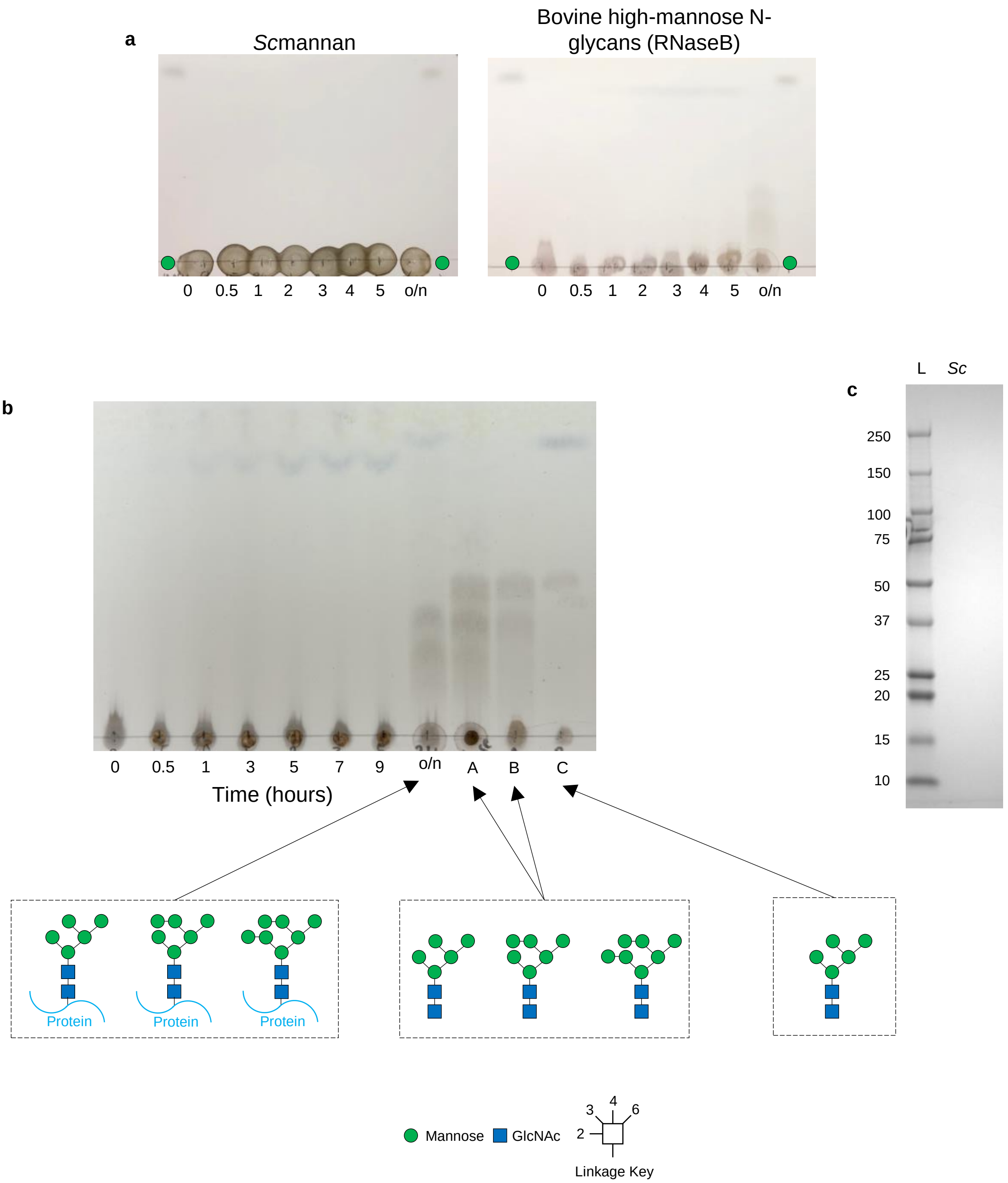

Supplementary Figure 38│Whole cell assay with high-mannose N-glycoprotein and *Sc*mannan. a, The results from the whole cell assay showing that no obvious degradation of the mannan is occurring (left) and a smear appears overnight for the high-mannose N-glycoprotein (right). **b,** TLC is repeated with controls. A: the overnight whole cell assay sample treated with PNGaseL, which is a broad-acting PNGase. B: RNaseB treated with PNGaseL. The smear seen here is a mix of high-mannose N-glycans of between 5 and 7 mannose sugars. C: RNaseB treated with PNGaseL and BT3990, which is an α1,2-mannosidase that trims all the high-mannose N-glycans down to a five-mannose structure. The reaction was carried out at 37 °C, a sample removed at different time points, and boiled to stop enzyme activities. Monosaccharide standards are shown in the right lanes. All mannose linkages are alpha. **c,** SDS-PAGE gel stained for protein using Coomassie Brilliant Blue. L – ladder and *Sc* – *Sc*mannan.

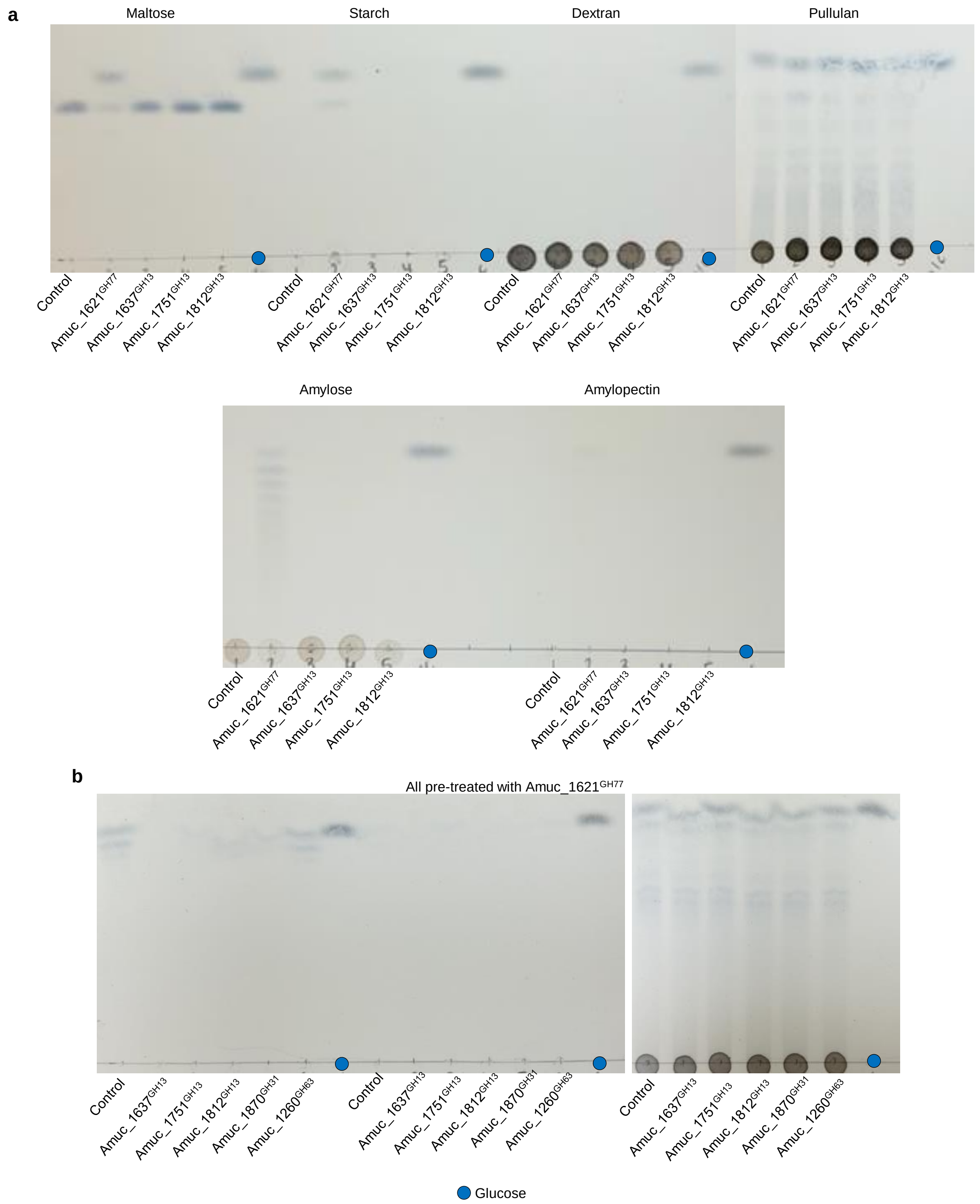

Supplementary Figure 39│Activities of the GH13 and GH77 family members from *A. muciniphila* ATCC BAA-835 against different α-linked glucose polysaccharides. Enzyme assays were carried out at pH 7, 37 °C, overnight, and with 1 μM enzymes. **a,** Assays of the individual enzymes against possible substrates. **b**, Assays where the substrates were treated with Amuc_1621^{GH77} as well as the other enzymes. Standards have also been included on the left of the TLCs.

Supplementary Figure 40│Full disclosure of TLCs and SDS-PAGE gels. Thin layer chromatography results of whole cell assays of PGM III-grown cells against different substrates.

Supplementary Figure 3

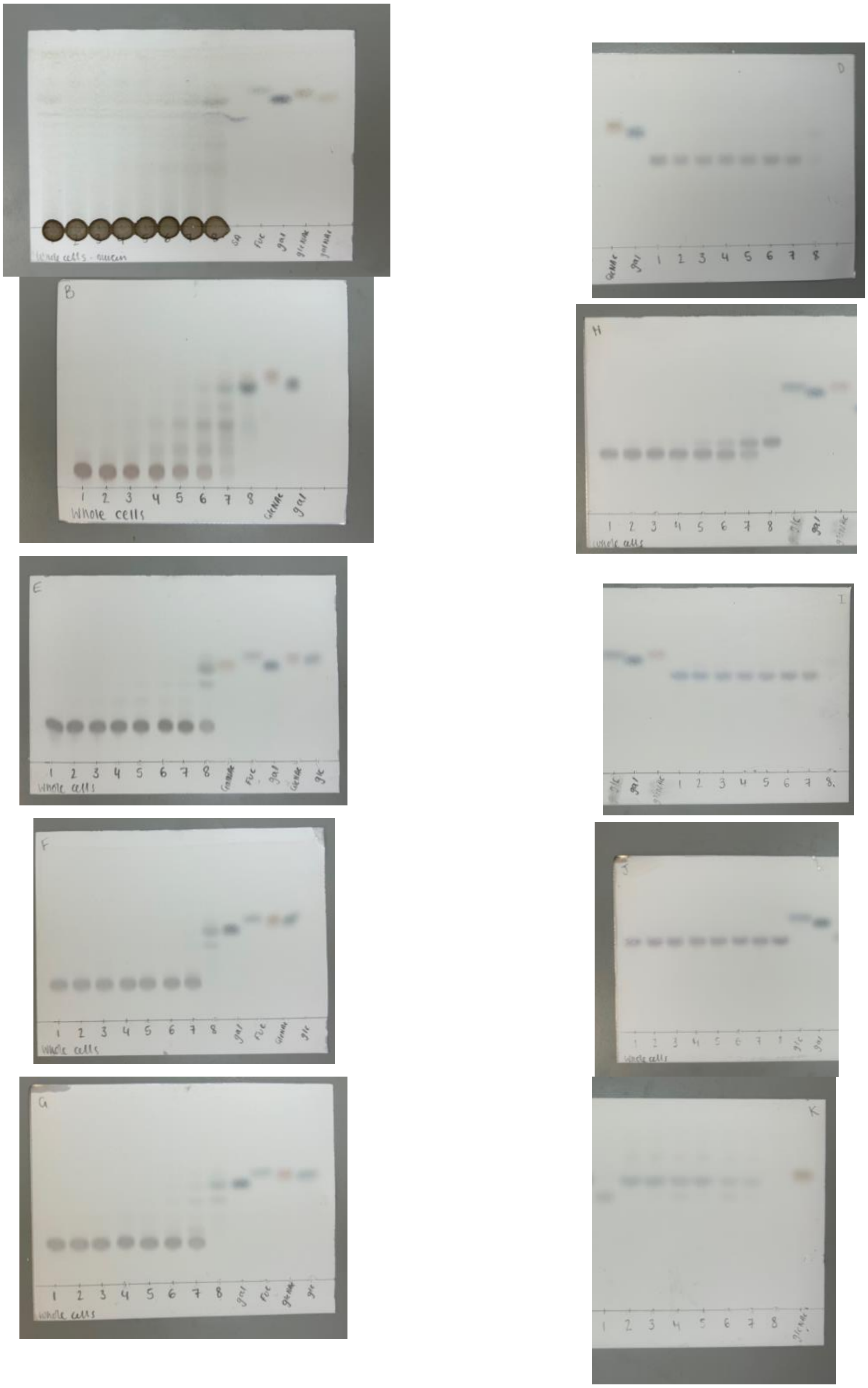

Supplementary Figure 4

a

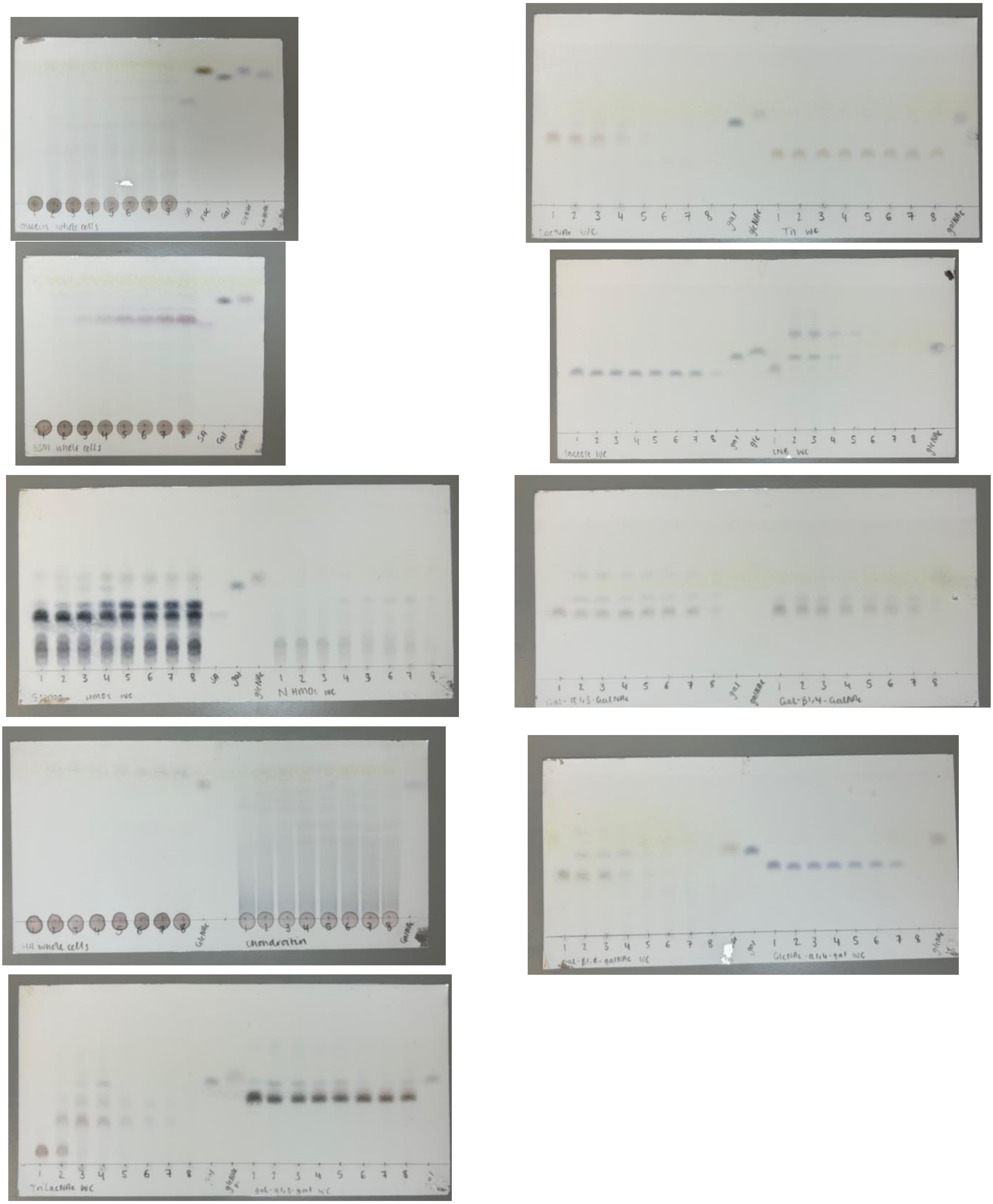

Supplementary Figure 7

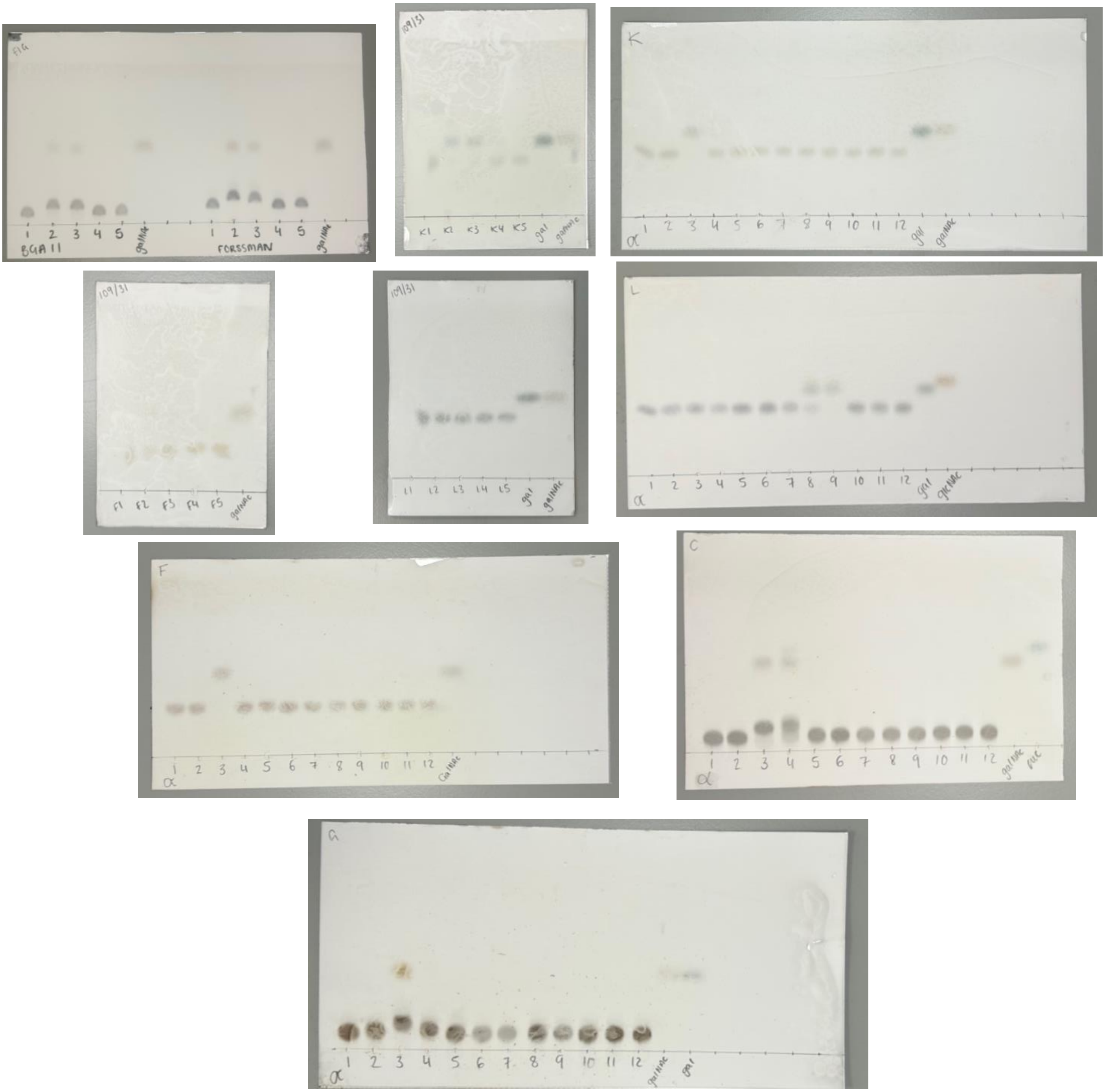

Supplementary Figure 8

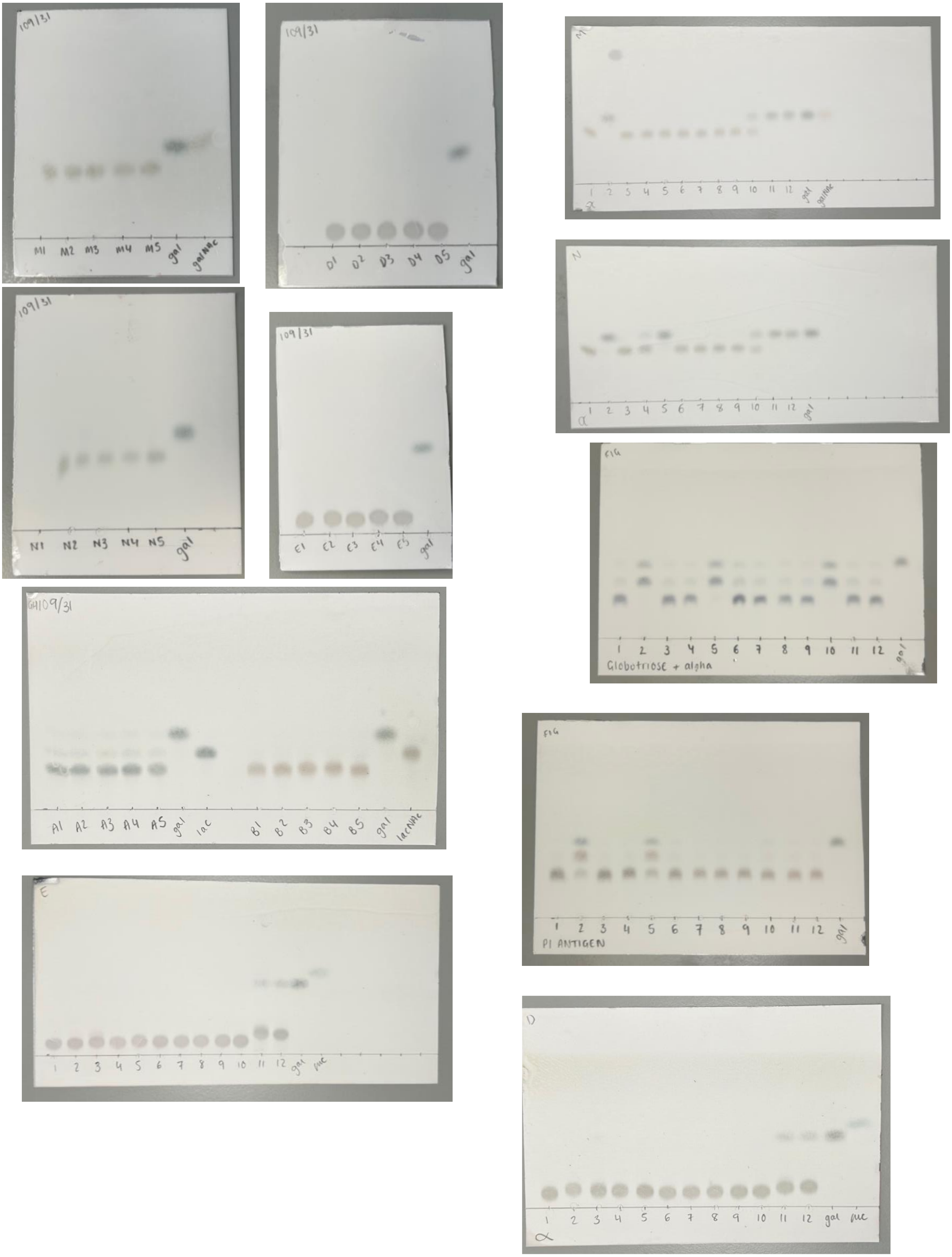

Supplementary Figure 9

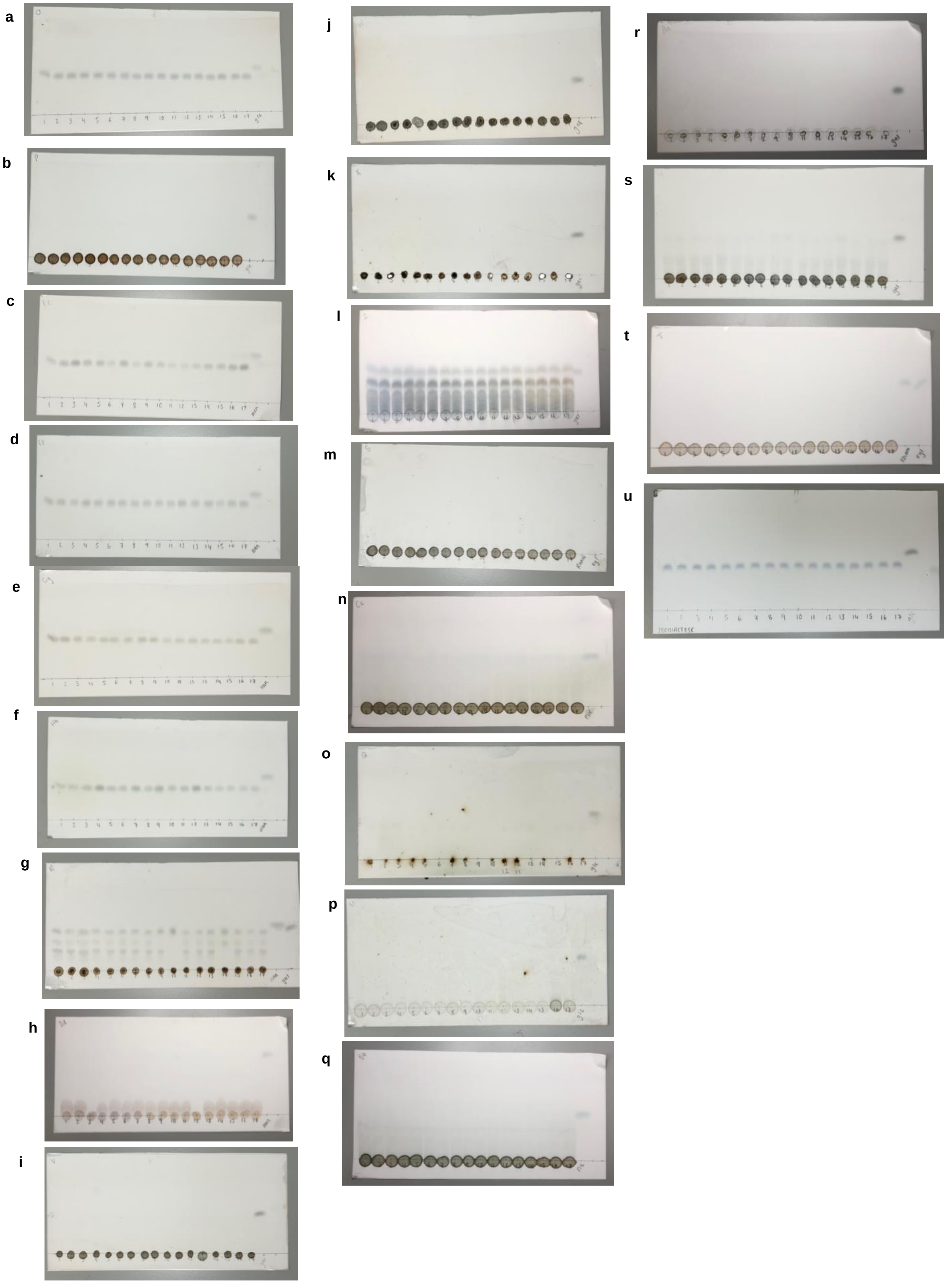

Supplementary Figure 10

f

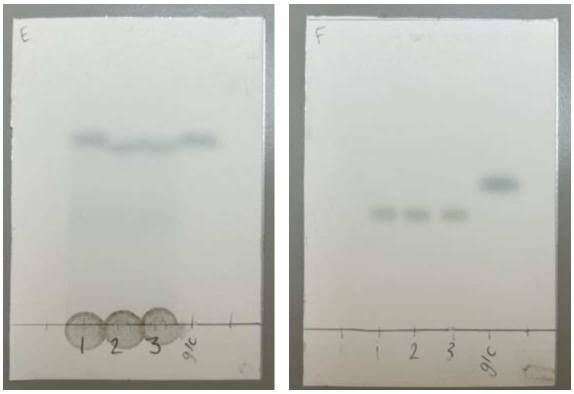

g

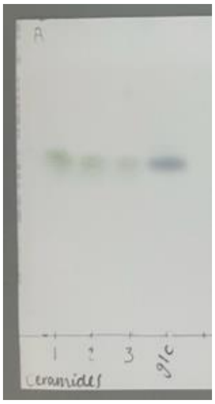

Supplementary Figure 14

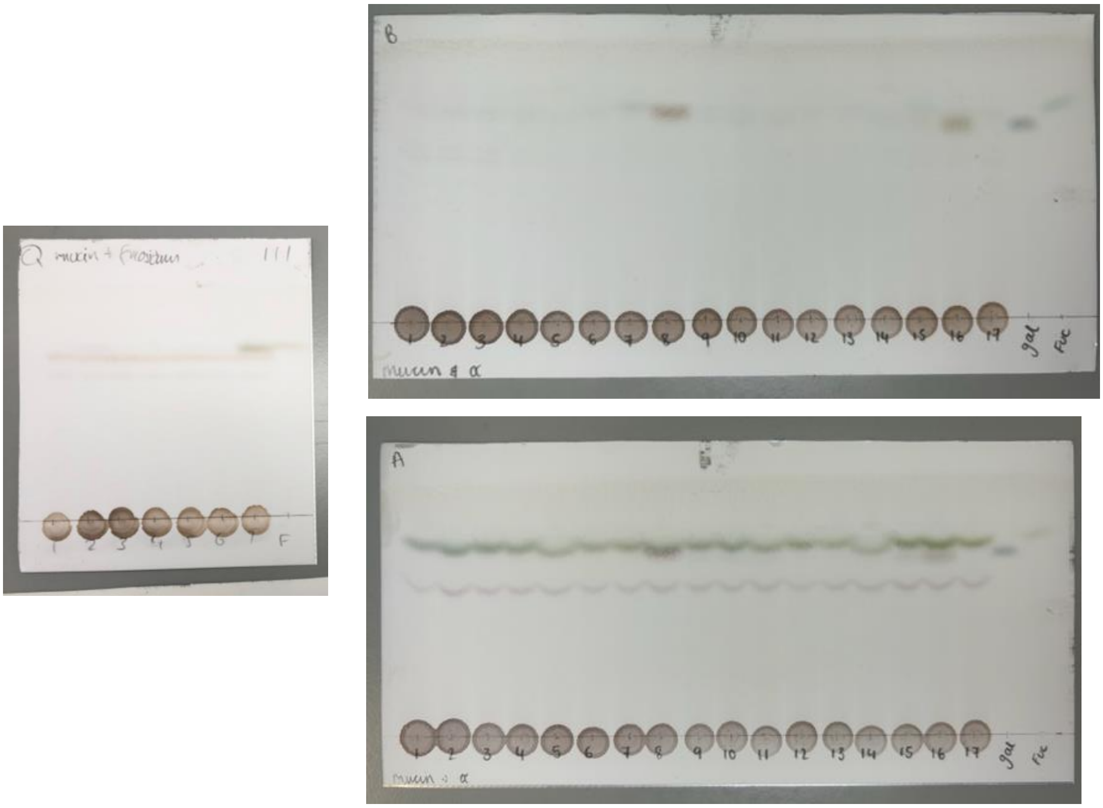

Supplementary Figure 16

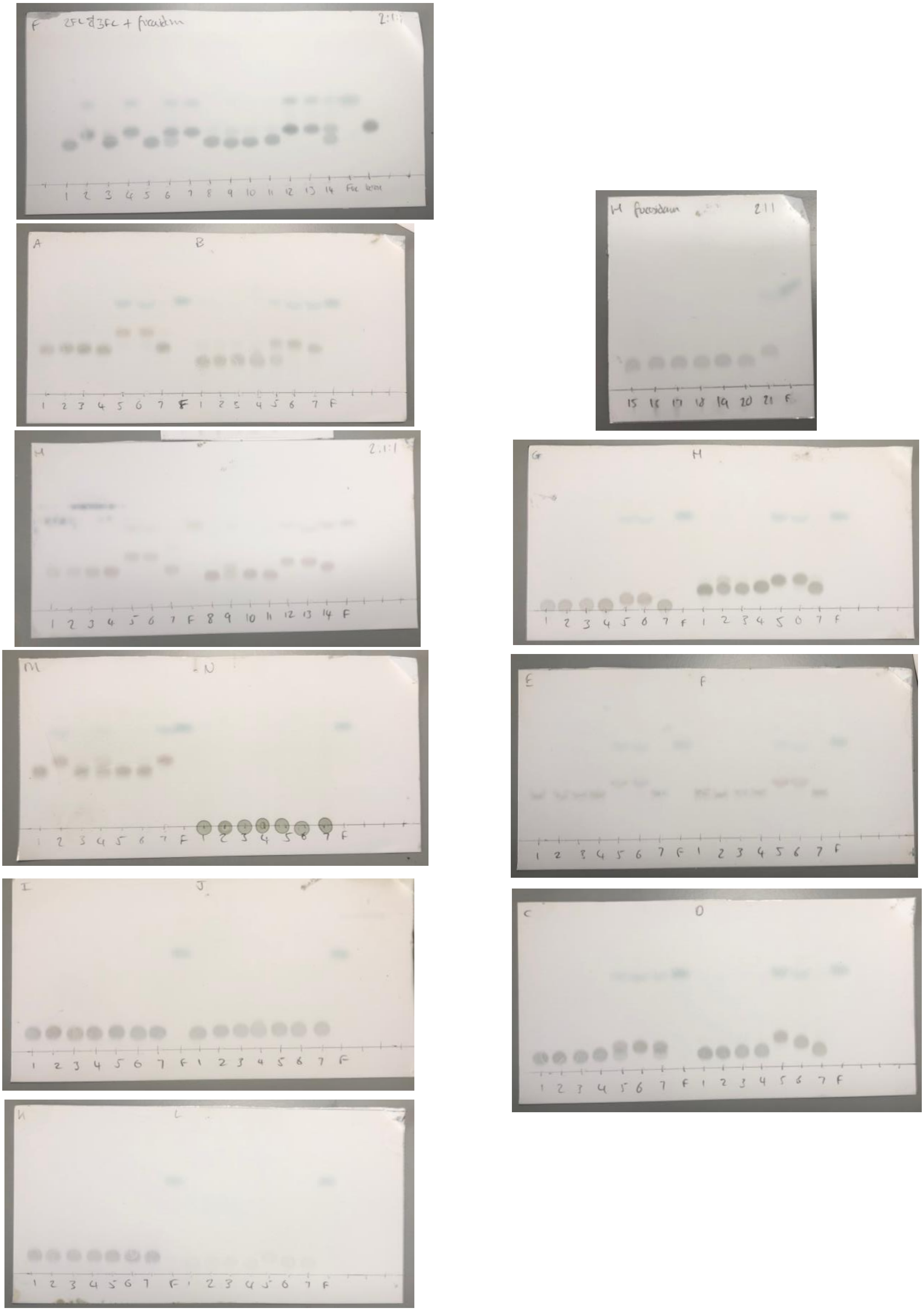

Supplementary Figure 18

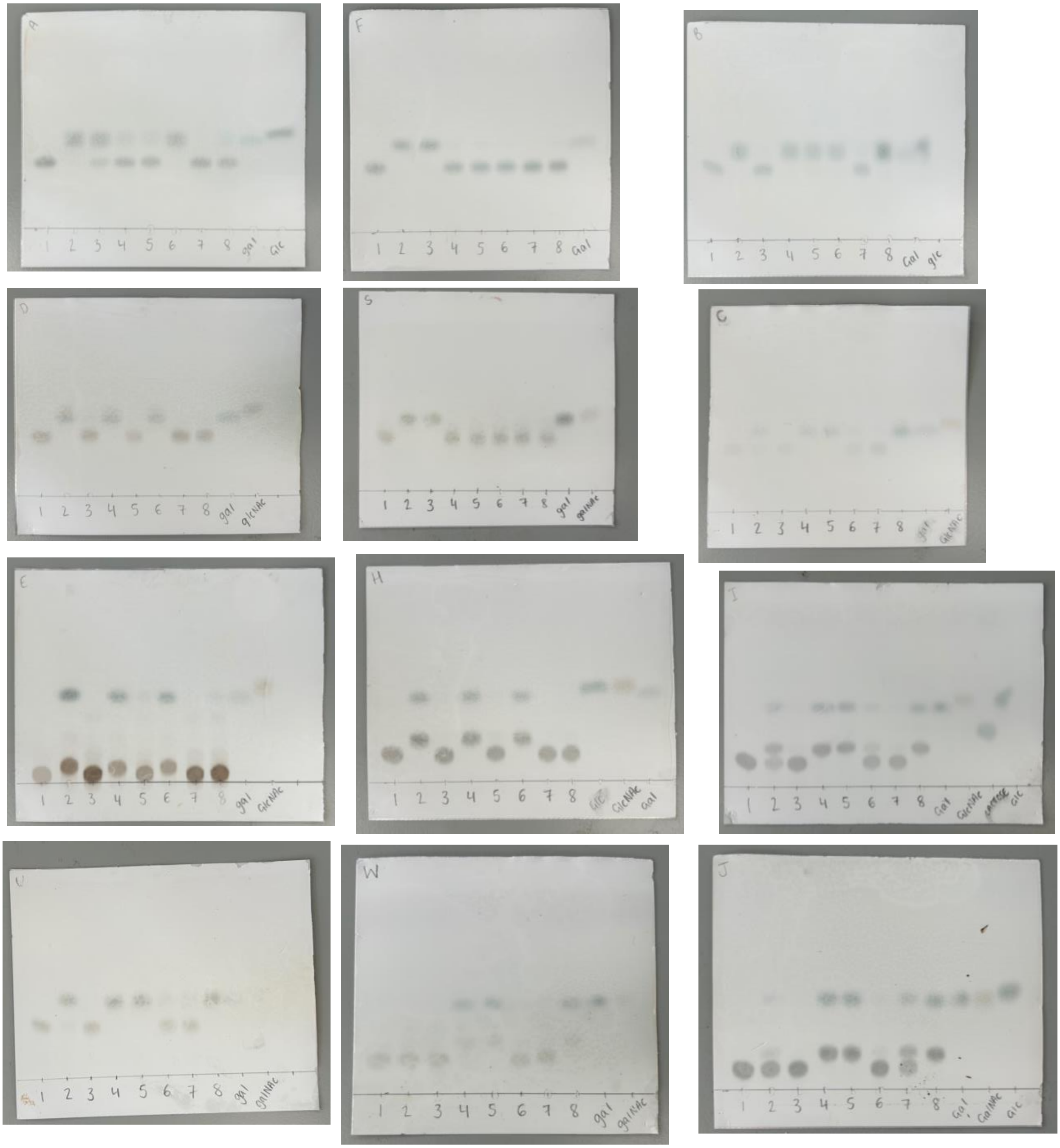

Supplementary Figure 19

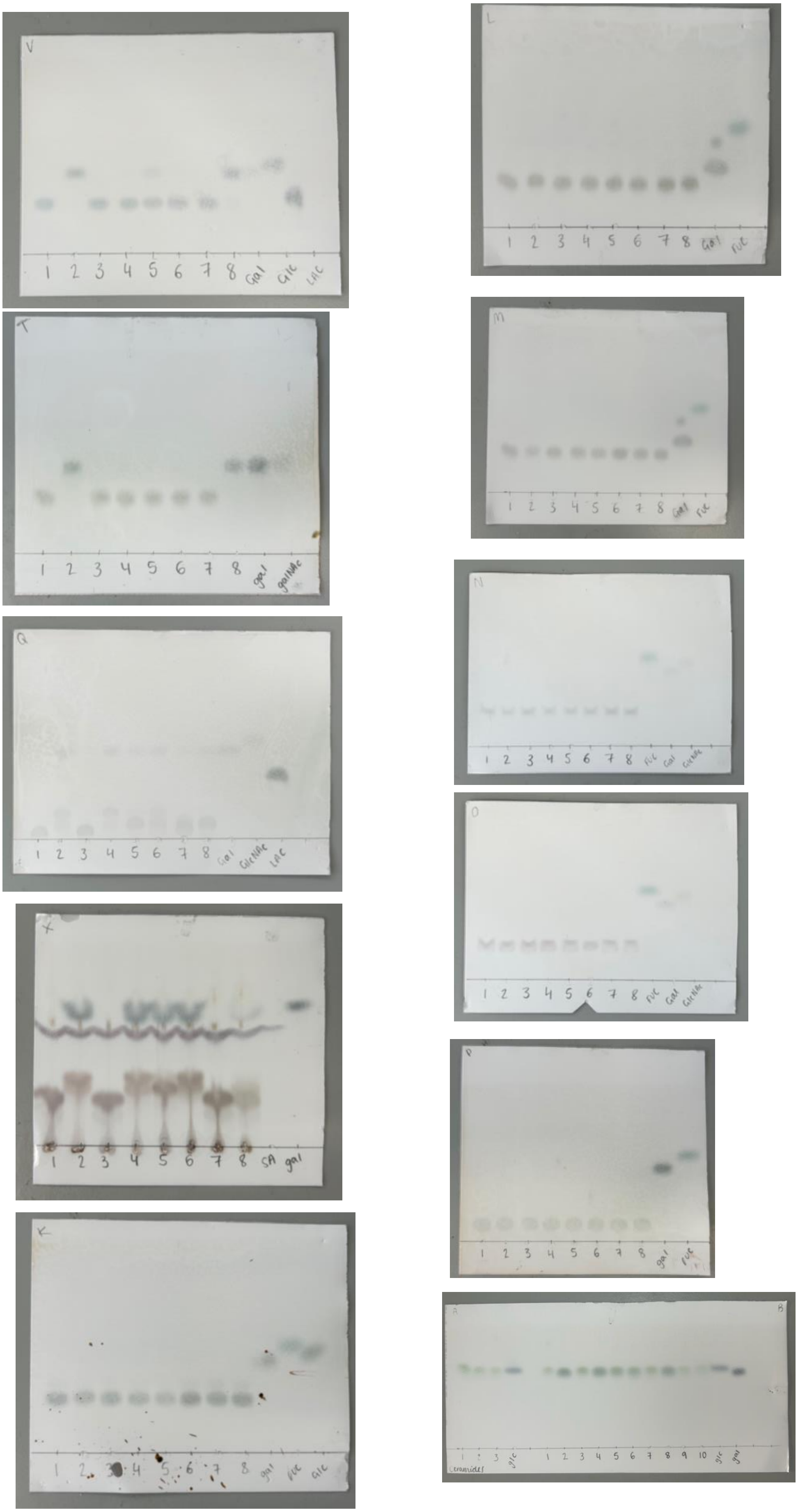

Supplementary Figure 20

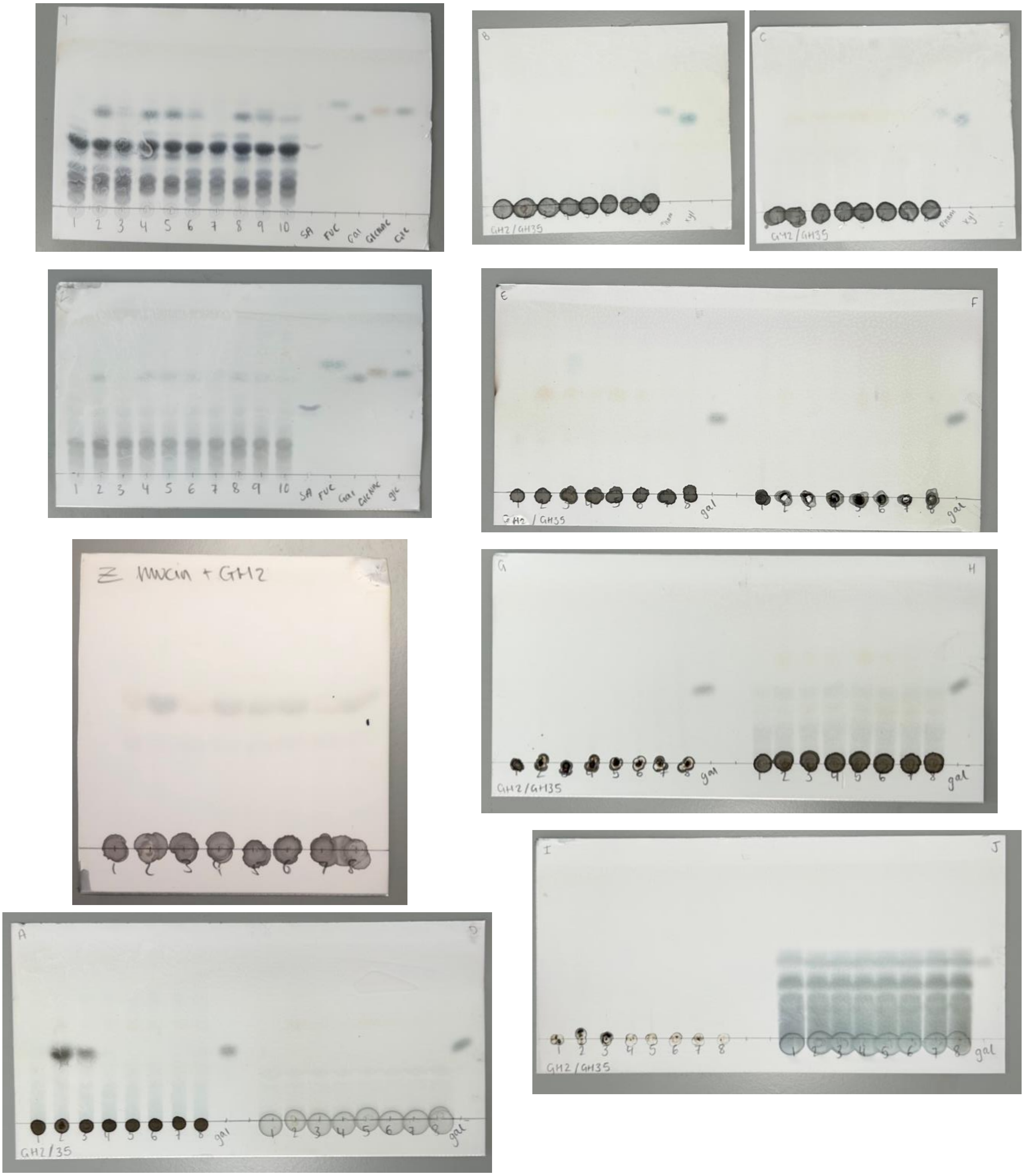

Supplementary Figure 21

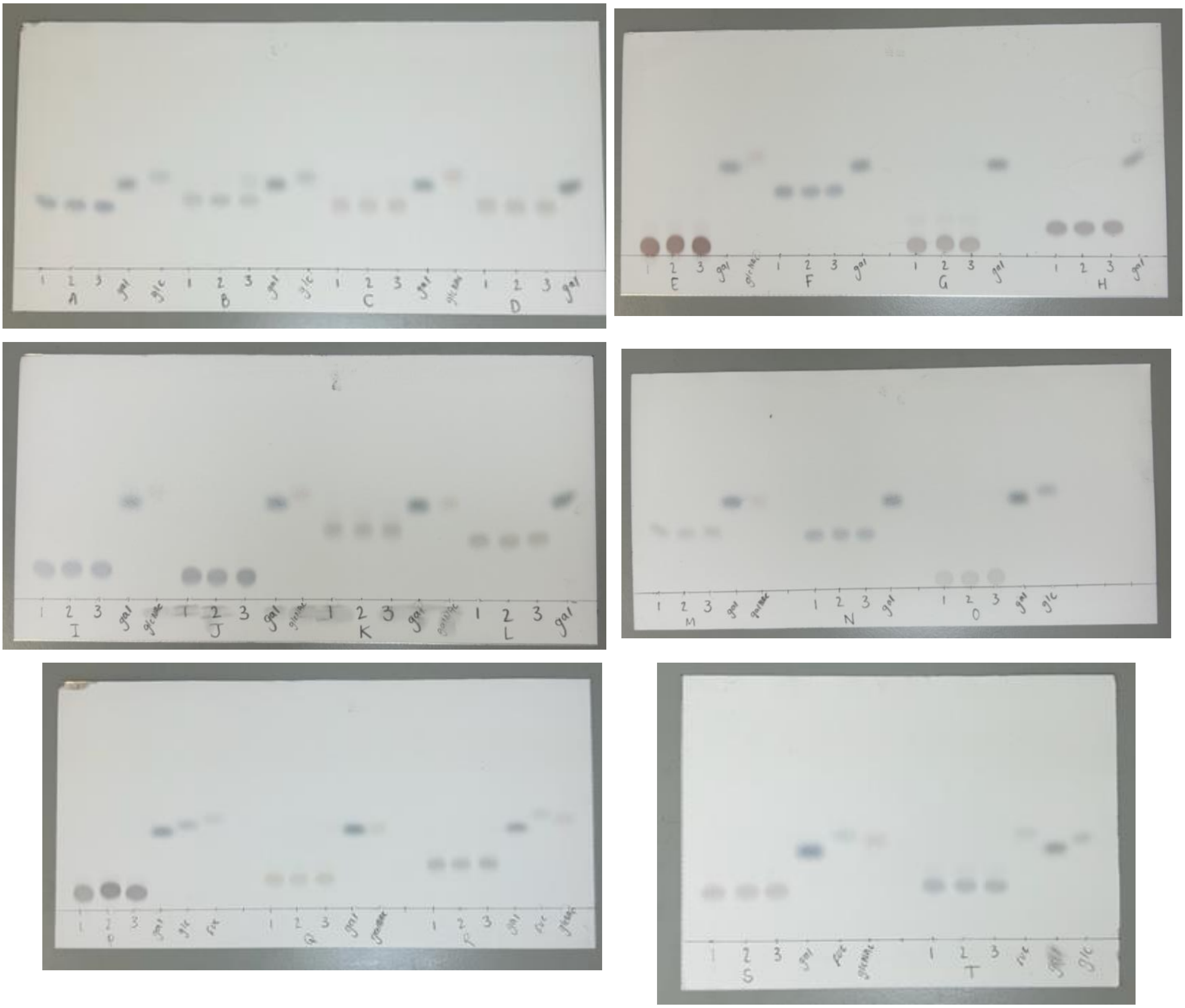

Supplementary Figure 27

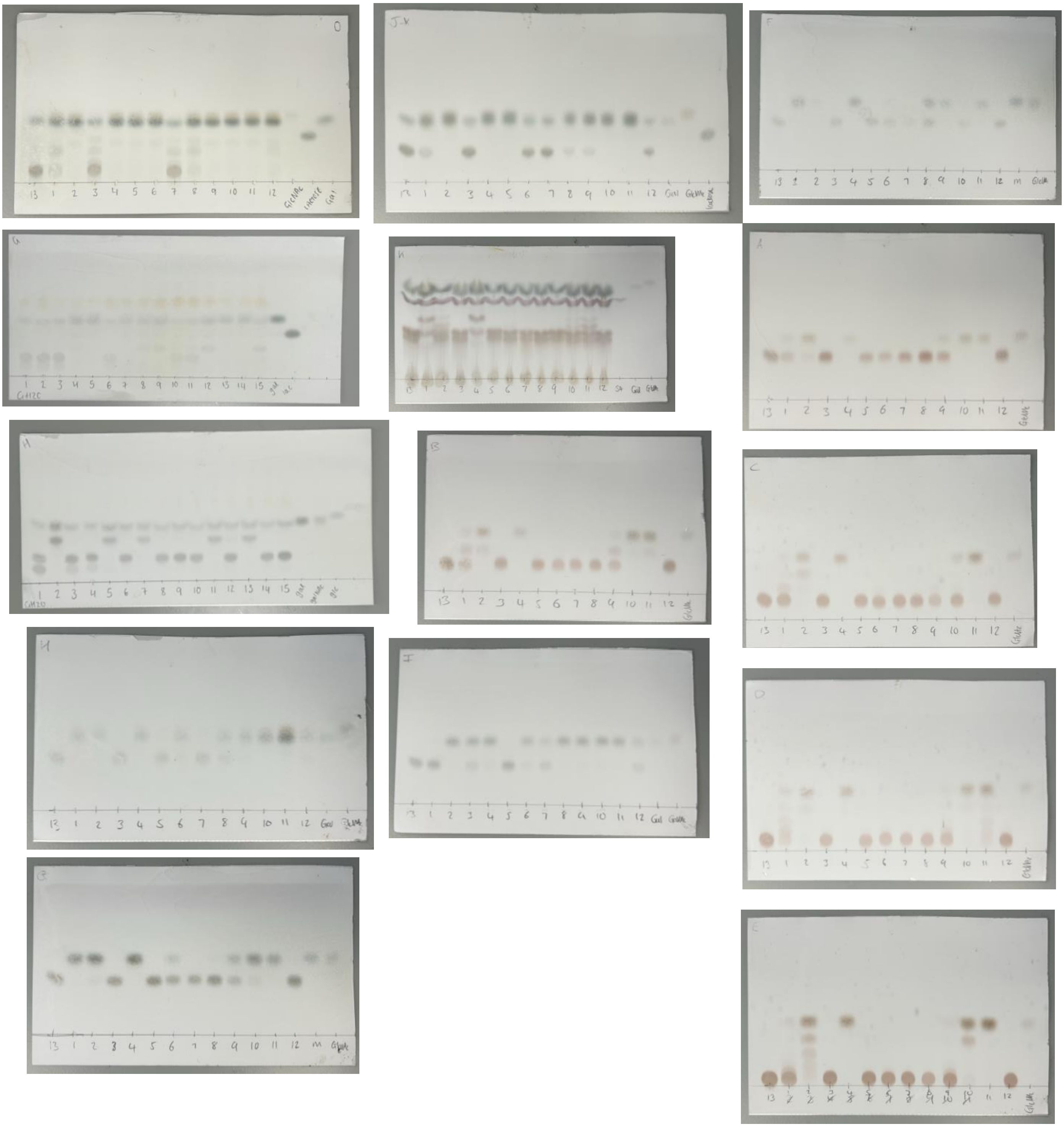

Supplementary Figure 28

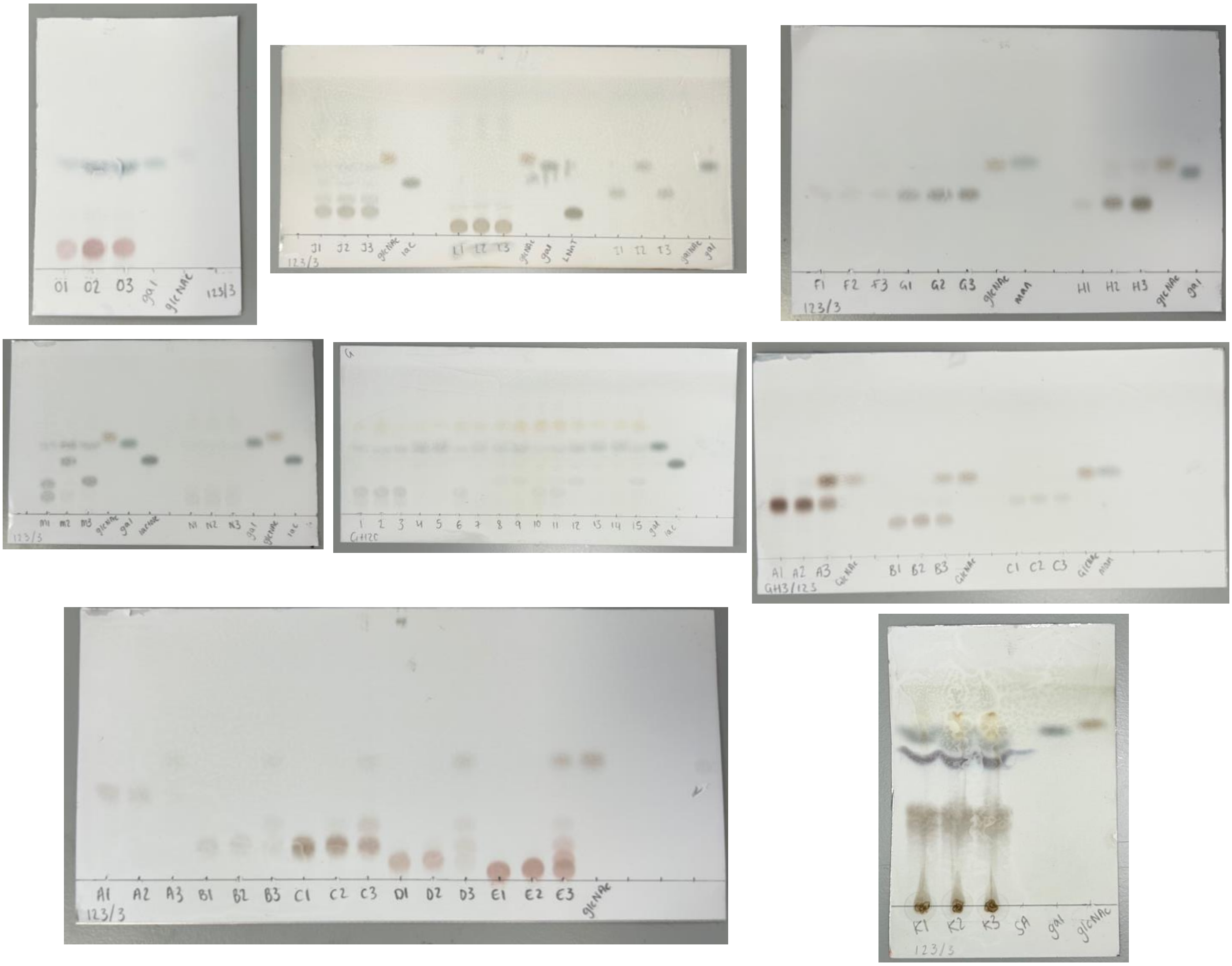

Supplementary Figure 29

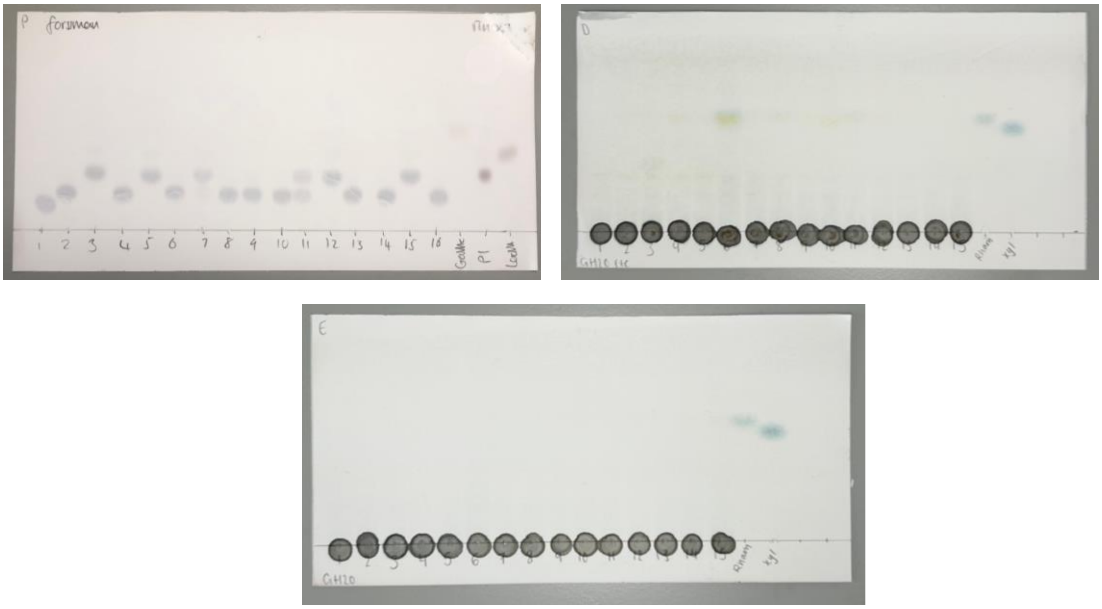

Supplementary Figure 30

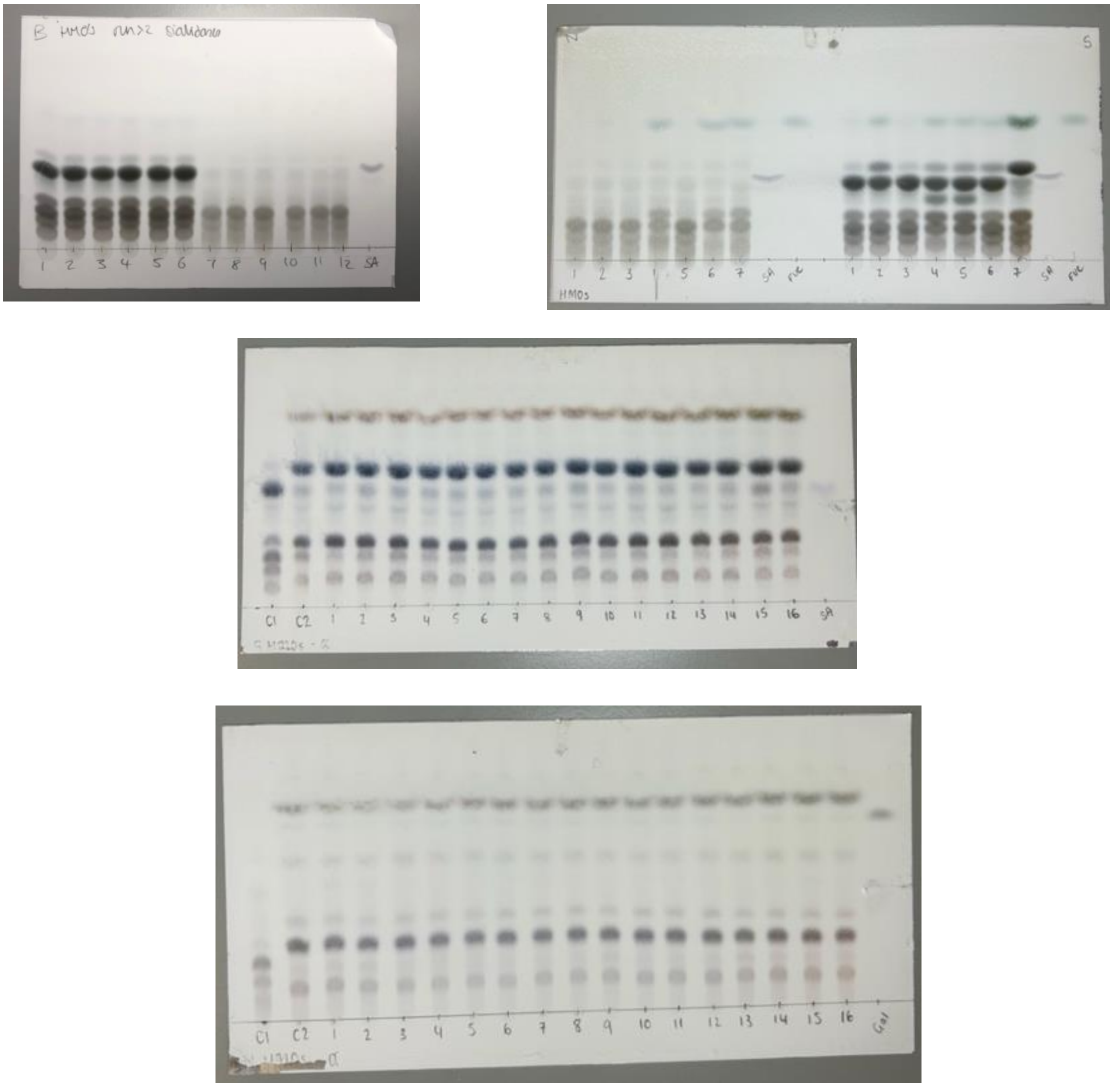

Supplementary Figure 31

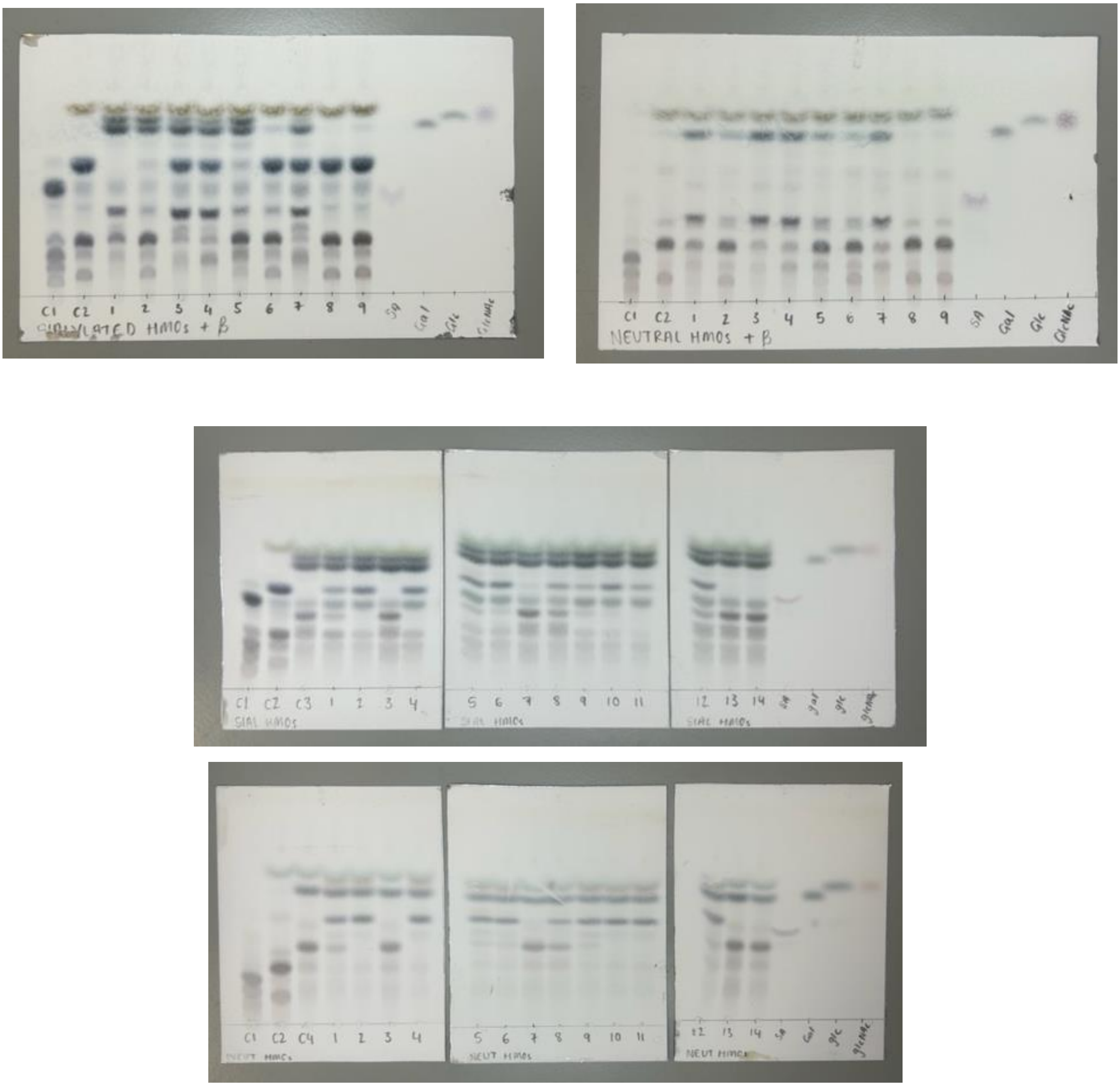

Supplementary Figure 33

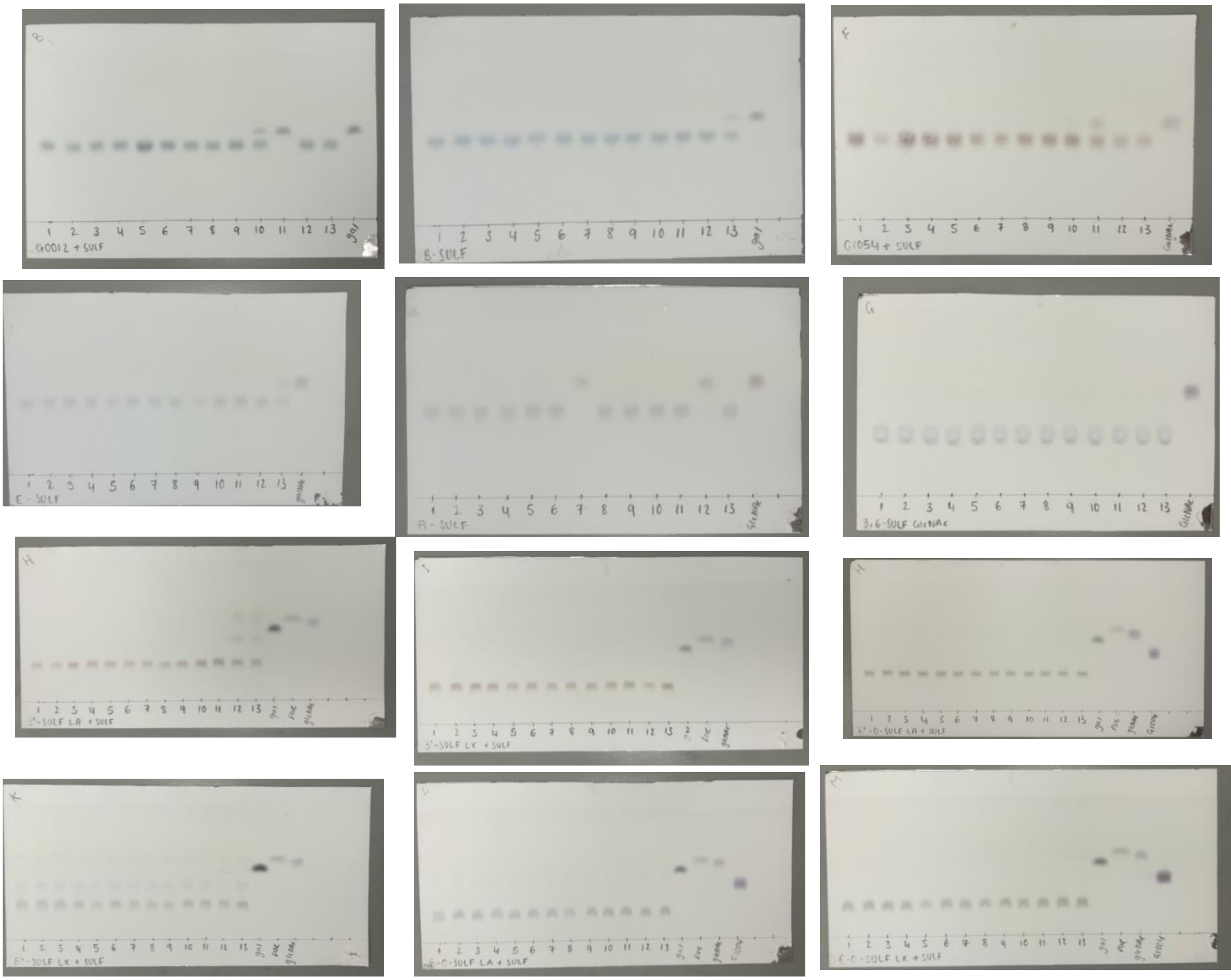

Supplementary Figure 34

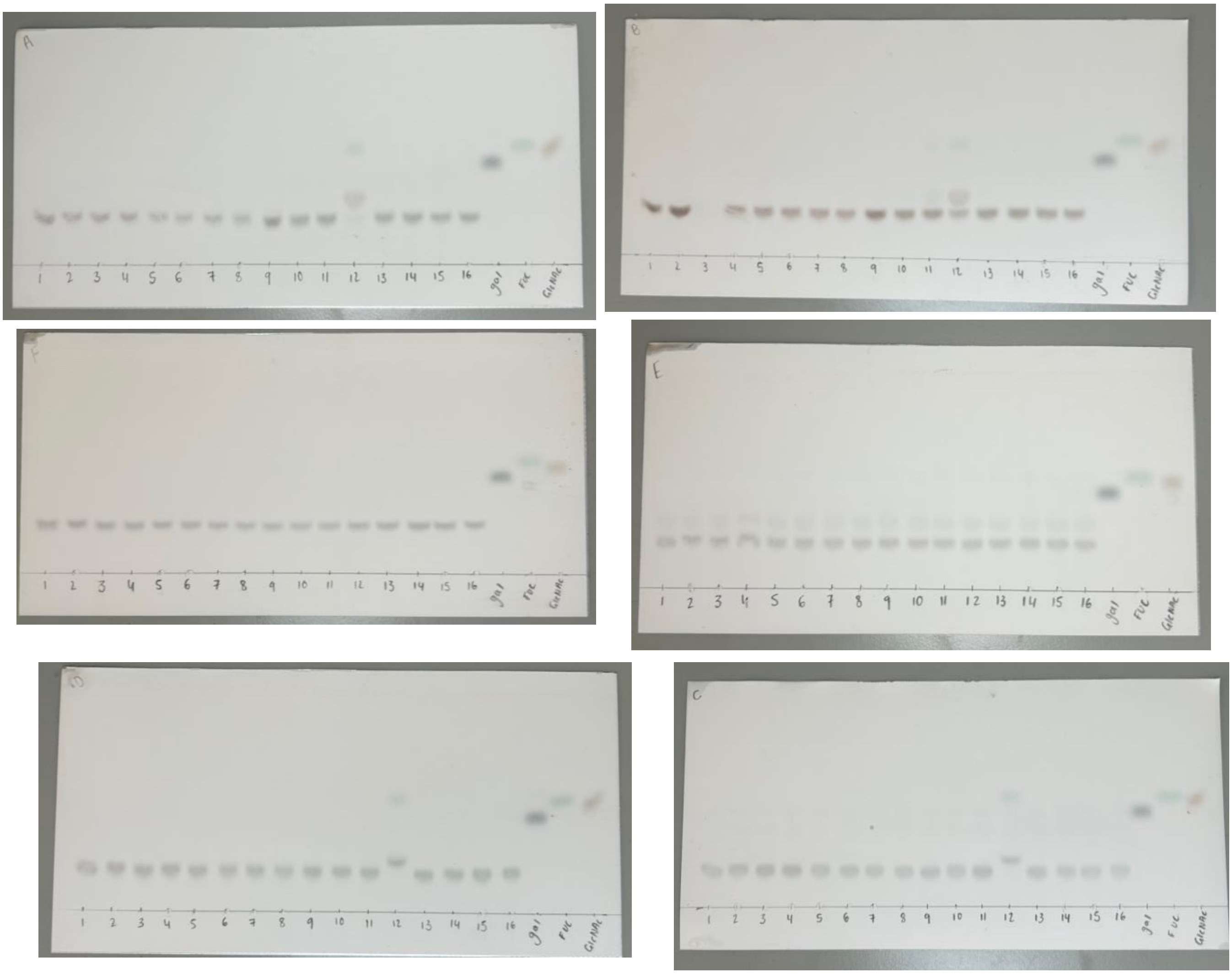

Supplementary Figure 35

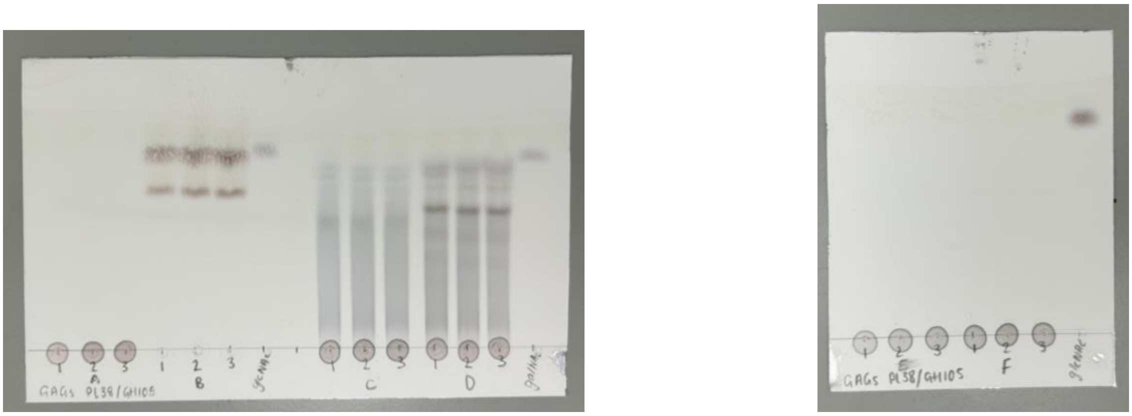

Supplementary Figure 37

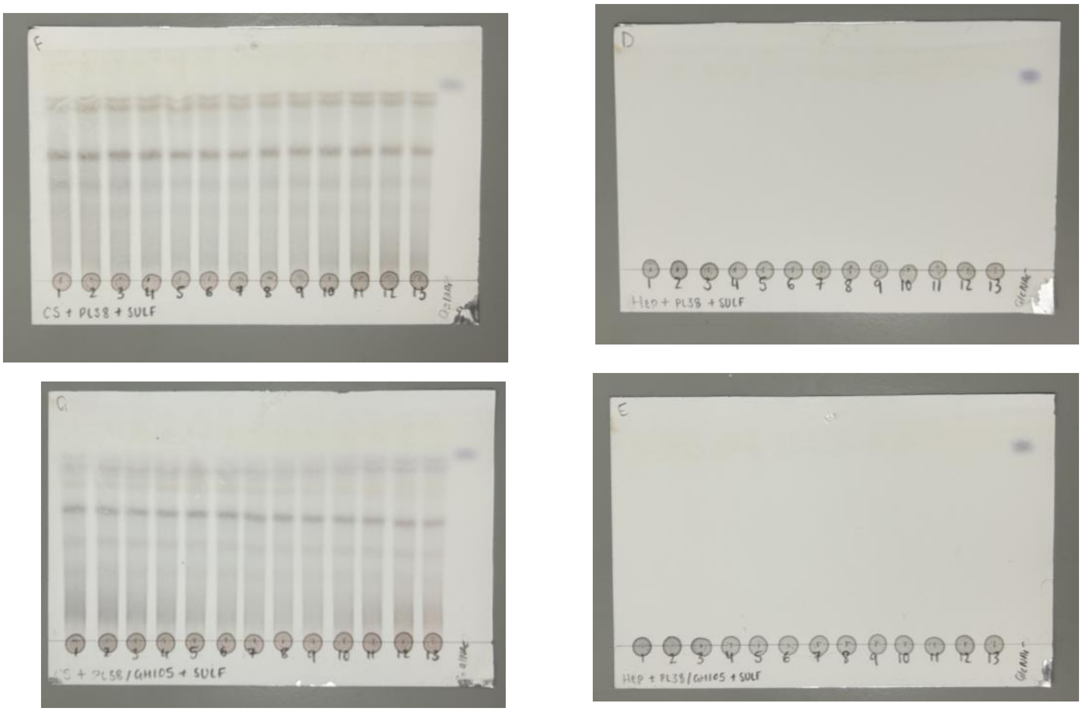

Supplementary Figure 38

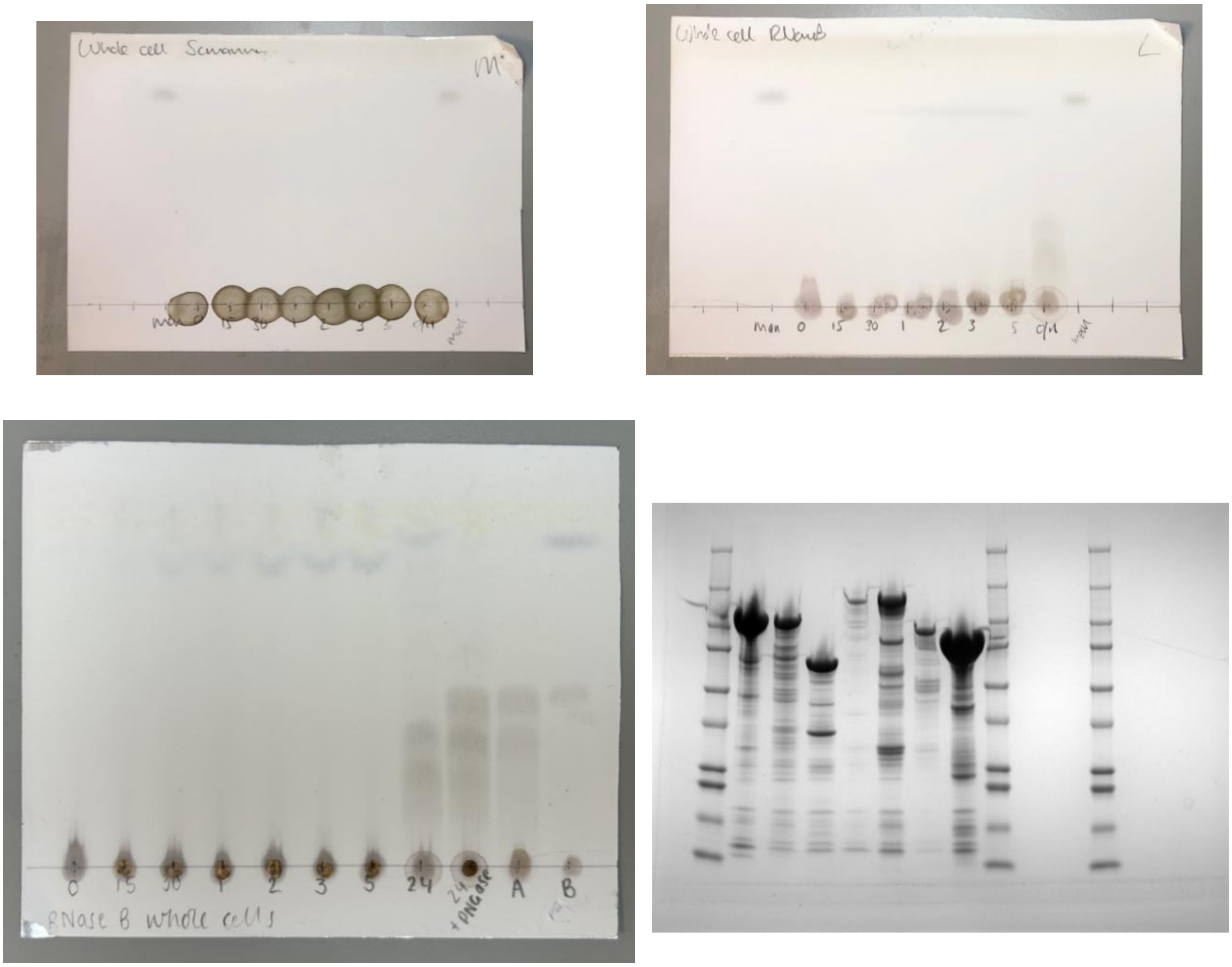

Supplementary Figure 39

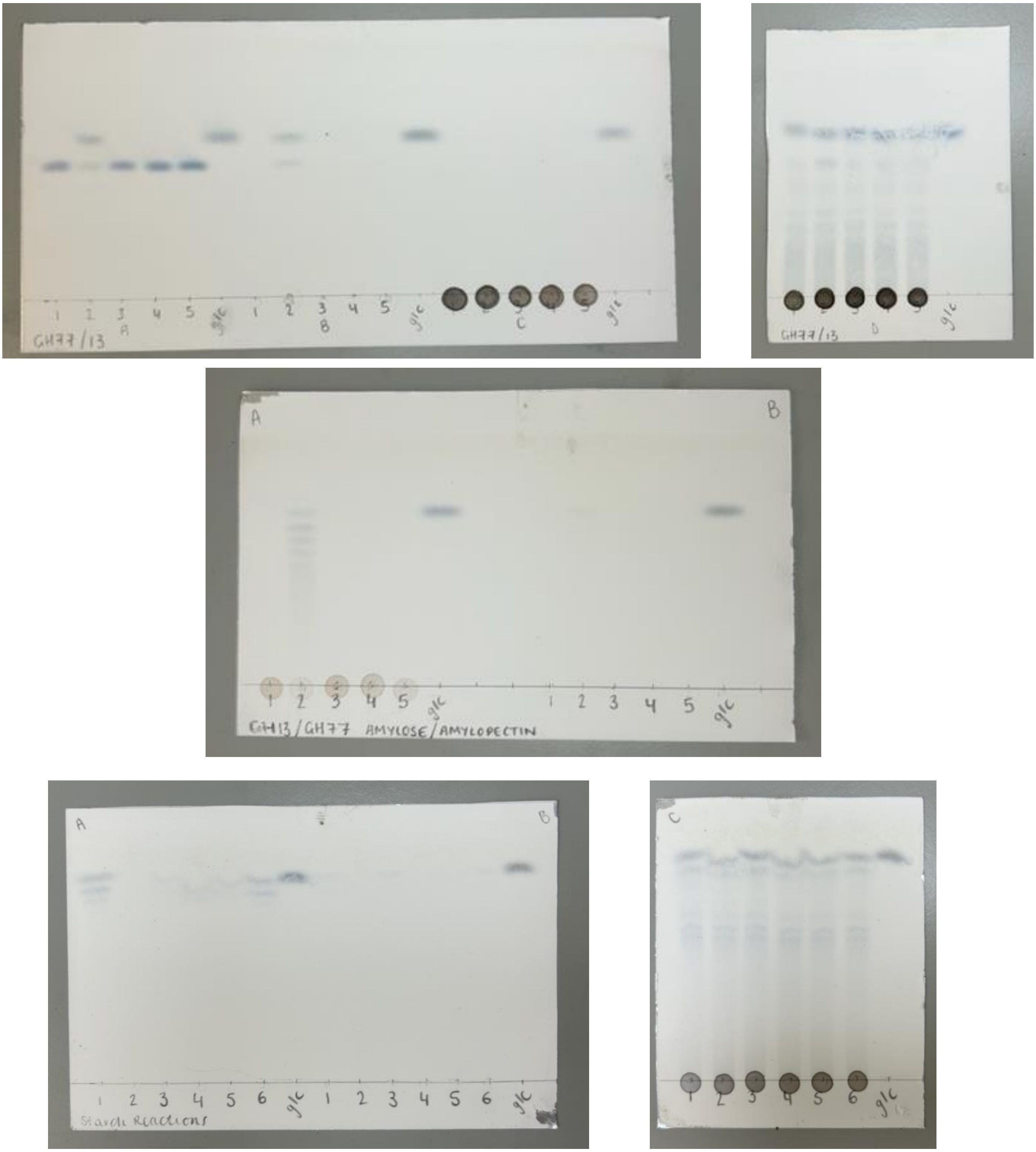

Supplement: Supplementary file 1 — Supplementary Discussion and Supplementary Figs. 1–40. [file 41564_2024_1911_MOESM1_ESM.pdf]
