# Supplementary material for: Carbohydrate-active enzymes from Akkermansia muciniphila break down mucin O-glycans to completion
Source: Nat Microbiol. 2025 Jan 31;10(2):585–98. doi: 10.1038/s41564-024-01911-7 (PMC11790493; doi:10.1038/s41564-024-01911-7)
Supplement: Supplementary file 2 — Reporting Summary [file 41564_2024_1911_MOESM2_ESM.pdf]

nature portfolio

Corresponding author(s): Lucy Crouch

Last updated by author(s): Nov 18, 2024

Reporting Summary

Nature Portfolio wishes to improve the reproducibility of the work that we publish. This form provides structure for consistency and transparency in reporting. For further information on Nature Portfolio policies, see our Editorial Policies and the Editorial Policy Checklist.

Statistics

For all statistical analyses, confirm that the following items are present in the figure legend, table legend, main text, or Methods section.

n/a	Confirmed	
☒	☐	The exact sample size (*n*) for each experimental group/condition, given as a discrete number and unit of measurement
☒	☐	A statement on whether measurements were taken from distinct samples or whether the same sample was measured repeatedly
☒	☐	The statistical test(s) used AND whether they are one- or two-sided<br>*Only common tests should be described solely by name; describe more complex techniques in the Methods section.*
☒	☐	A description of all covariates tested
☒	☐	A description of any assumptions or corrections, such as tests of normality and adjustment for multiple comparisons
☐	☒	A full description of the statistical parameters including central tendency (e.g. means) or other basic estimates (e.g. regression coefficient) AND variation (e.g. standard deviation) or associated estimates of uncertainty (e.g. confidence intervals)
☒	☐	For null hypothesis testing, the test statistic (e.g. *F*, *t*, *r*) with confidence intervals, effect sizes, degrees of freedom and *P* value noted<br>*Give P values as exact values whenever suitable.*
☒	☐	For Bayesian analysis, information on the choice of priors and Markov chain Monte Carlo settings
☒	☐	For hierarchical and complex designs, identification of the appropriate level for tests and full reporting of outcomes
☒	☐	Estimates of effect sizes (e.g. Cohen's *d*, Pearson's *r*), indicating how they were calculated

Our web collection on statistics for biologists contains articles on many of the points above.

Software and code

Policy information about availability of computer code

Data collection	Chromeleon Chromatography Data System Software v7.3, AlphFold2 Collab, SignalP 6.0, UNIPROT, Bio3d from RStudio, Tabulartofasta from GitHub, Bruker Compass HyStar 5.1.8.1 to collect GAG data, Procainamide-labelling data: Thermo Scientific UltiMate 3000 UPLC instrument with a fluorescence detector controlled by HyStar software version 3.2. MS data collection: AmaZon Speed ETD electrospray mass spectrometer (Bruker Daltonics, Bremen, Germany).
Data analysis	Graphpad Prism to produce graphs and heatmaps, ClustalOmega to generate sequence alignments, Pymol to produce structural images, Coot to overlay structures, GLYCAM carbohydrate builder, SeaView to generate phylogeny trees, iTOL to present phylogeny trees, Bruker Compass Data Analysis 6.1 to analyse GAG data, TASQ 2.2 for relative compound quantification of GAGs, ChemDraw 18.1 was used to draw carbohydrate structures, RNA-seq data processing: fastp v.0.23.2, kallisto v.0.46.2, Voom/limma v.3.40.6, Degust v.4.2-dev, Volcano plot produced using R v.4.4.0 and EnhancedVolcano v.1.22.0. Procainamide-labelling data analysis: ESI-MS and MS/MS data analysis was performed using Bruker Compass DataAnalysis V4.1 software.

For manuscripts utilizing custom algorithms or software that are central to the research but not yet described in published literature, software must be made available to editors and reviewers. We strongly encourage code deposition in a community repository (e.g. GitHub). See the Nature Portfolio guidelines for submitting code & software for further information.

Data

Policy information about availability of data

All manuscripts must include a data availability statement. This statement should provide the following information, where applicable:

- Accession codes, unique identifiers, or web links for publicly available datasets
- A description of any restrictions on data availability
- For clinical datasets or third party data, please ensure that the statement adheres to our policy

The full RNA-seq data are provided in Supplementary Data 1 and submitted to https://www.ebi.ac.uk/ena/browser/home with accession number PRJEB76658. The data that support the findings presented in this manuscript are available upon request from the corresponding authors.

Research involving human participants, their data, or biological material

Policy information about studies with human participants or human data. See also policy information about sex, gender (identity/presentation), and sexual orientation and race, ethnicity and racism.

Reporting on sex and gender	N/A
Reporting on race, ethnicity, or other socially relevant groupings	N/A
Population characteristics	N/A
Recruitment	N/A
Ethics oversight	N/A

Note that full information on the approval of the study protocol must also be provided in the manuscript.

Field-specific reporting

Please select the one below that is the best fit for your research. If you are not sure, read the appropriate sections before making your selection.

☒ Life sciences ☐ Behavioural & social sciences ☐ Ecological, evolutionary & environmental sciences

For a reference copy of the document with all sections, see nature.com/documents/nr-reporting-summary-flat.pdf

Life sciences study design

All studies must disclose on these points even when the disclosure is negative.

Sample size	No sample size calculation was performed during this work. Bacterial growth on different glycans/monosaccharides was carried out in triplicate and the experiments repeated at least once (ie. minimum of two biological replicates with triplicate technical replicates each time). Non-kinetic enzyme assays were repeated at least once for each substrate tested with different enzyme preparations. For kinetics against GAGs, the enzymes were produced at least twice and no variability in rates was observed between enzyme preparations.
Data exclusions	A few individual growths curves have been removed at the request of a reviewer.
Replication	Growth assays were generally repeated at least twice. Exceptions to this were for the dialysed Scmannan experiment and the different ratios of mucin:GAG, which were only carried out once. Assays with defined oligosaccharides have been carried out at least twice. The BSM assays to quantify monosaccharide through HPAEC-PAD was carried out in triplicate. The assays using the alpha enzymes against PGM was carried out once, with and without sialidase and fucosidase. Two separate whole cell assays were carried out. HPAEC-PAD data from the BSM whole cell assay was carried out once. LC-ESI-FLD-MS was carried out once as when we get to the stage of procainamide labelling a sample for characterization, we are sure that the experiment is reproducible and this process is very expensive. Comparison between the different LC-ESI-FLD-MS samples also provides confidence in the results. RNA-seq was carried out in triplicate.
Randomization	Randomization was not necessary for any of the experiments carried out during this work. We always have negative controls and, where possible, positive controls
Blinding	True blinding was not necessary for any of the experiments carried out during this work. Throughout the production of enzymes, the locus tags were used, but the researchers were not told to expect particular activities. Substrates were often provided letters and the samples (control/enzyme) with numbers to set up and track assays easily. The identity of substrate and enzyme were then reassigned after the data had been collected. Growth curves in 96-well plates were subject to similar coding, which were again reassigned when looking at the data.

Reporting for specific materials, systems and methods

We require information from authors about some types of materials, experimental systems and methods used in many studies. Here, indicate whether each material, system or method listed is relevant to your study. If you are not sure if a list item applies to your research, read the appropriate section before selecting a response.

Materials & experimental systems

n/a	Involved in the study
☒	☐ Antibodies
☒	☐ Eukaryotic cell lines
☒	☐ Palaeontology and archaeology
☒	☐ Animals and other organisms
☒	☐ Clinical data
☒	☐ Dual use research of concern
☒	☐ Plants

Methods

n/a	Involved in the study
☒	☐ ChIP-seq
☒	☐ Flow cytometry
☒	☐ MRI-based neuroimaging

Plants

Seed stocks	N/A
Novel plant genotypes	N/A
Authentication	N/A
